# Three-component synthesis of highly functionalized aziridines containing a peptide side chain and their one-step transformation into β-functionalized α-ketoamides

**DOI:** 10.3762/bjoc.12.166

**Published:** 2016-08-08

**Authors:** Lena Huck, Juan F González, Elena de la Cuesta, J Carlos Menéndez

**Affiliations:** 1 Departmento de Química Orgánica y Farmacéutica, Facultad de Farmacia, Universidad Complutense, 28040 Madrid, Spain

**Keywords:** α-ketoamides, aziridines, multicomponent reactions, nitrogen heterocycles, one-pot reactions, peptide mimics, vicinal tricarbonyl compounds

## Abstract

A sequential three-component process is described, starting from 3-arylmethylene-2,5-piperazinediones and involving a one-pot sequence of reactions achieving regioselective opening of the 2,5-diketopiperazine ring and diastereoselective generation of an aziridine ring. This method allows the preparation of *N*-unprotected, trisubstituted aziridines bearing a peptide side chain under mild conditions. Their transformation into β-trifluoroacetamido-α-ketoamide and α,β-diketoamide frameworks was also achieved in a single step.

## Introduction

Aziridine moieties occur in many natural products and are also encountered in unnatural biologically active compounds including drugs in clinical use, especially in the field of cancer treatment [1]. In the areas of medicinal chemistry and chemical biology, aziridines are of current interest as starting materials for the preparation of peptides [2–3] and the synthesis of peptide-like compounds comprising the aziridine motif [4–5]. Furthermore, the incorporation of an electrophilic aziridine moiety in peptide or peptidomimetic frameworks is an interesting strategy for the design of active site-directed drugs, especially in the field of chemotherapy [6–7].

Aziridines also have an important role in the preparation of further types of nitrogen-containing compounds [8–10]. As a consequence, the preparation of aziridines is of great importance [11–13]. The primary synthetic approaches involve either the direct aziridination of an olefin [14] or the reaction of an imine with diazoalkanes. Alternatively, the reaction of imines with various nucleophiles, including aza-Darzens mechanisms, can lead to aziridine products [15]. However, few known methods allow the synthesis of C-trisubstituted aziridines, particularly for the case of *N*-unprotected systems [10,16–17].

**Scheme 1:**
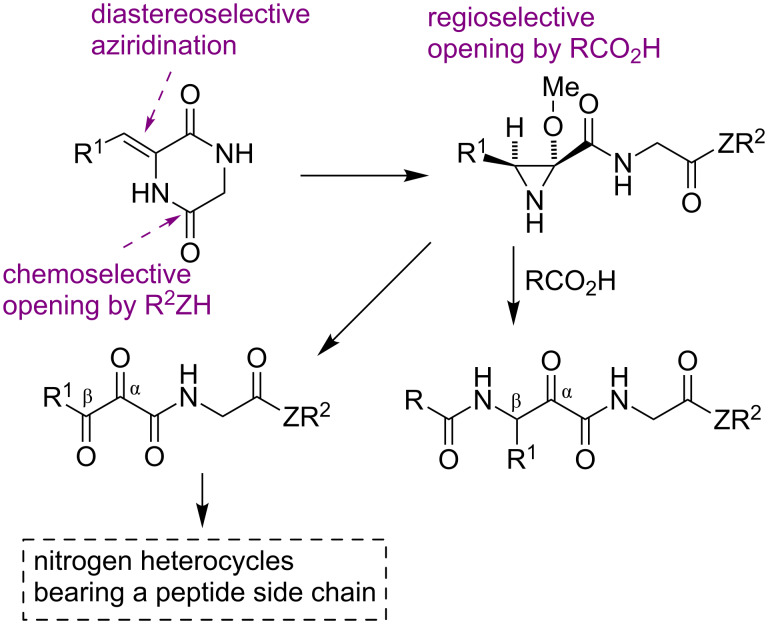
Summary of the work described in this paper.

2,5-Diketopiperazines (DKPs) are readily accessible from amino acids and are versatile synthetic scaffolds [18–19]. Consequently, there is considerable interest in the development of synthetic methodology based on the cleavage of suitably functionalized DKP derivatives [20–22]. In this context, we describe here an efficient regio- and diastereoselective one-pot three-component assembly of trisubstituted aziridines possessing a free NH group and containing a peptidic or peptide-like side chain. Furthermore, we describe the conversion of these compounds into functionalized β-amino-α-ketoamides. This is an important structural fragment that is used in the design of reversible covalent enzyme inhibitors, including the anti-hepatitis C drugs boceprevir and telaprevir, because the electrophilic keto group is able to react reversibly with mercapto or hydroxy groups at the active sites of a variety of enzymes. Existing synthetic approaches to this moiety are normally quite complex and do not easily allow the presence of amino acid side chains on the amide nitrogen [23]. Finally, in order to further underscore the synthetic usefulness of these aziridine scaffolds, we examined their one-pot transformation into α,β-diketoamides and the transformation of the latter compounds into nitrogen heterocycles bearing a peptide side chain (Scheme 1).

## Results and Discussion

**Scheme 2:**
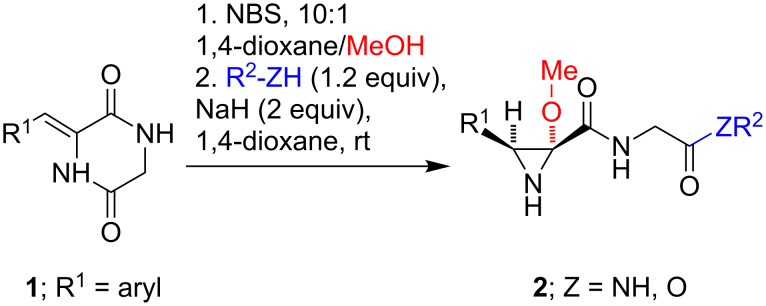
Synthesis of aziridines **2**.

The starting 3-arylmethylene-2,5-piperazinediones **1** were readily available by aldol condensation of 1,4-diacetyl-2,5-piperazinedione with aldehydes in the presence of potassium *tert*-butoxide, followed by deacetylation with hydrazine hydrate (see the Supporting Information File 1 for further details). As shown in Scheme 2, treatment of these materials with *N*-bromosuccinimide and methanol in dioxane followed by the addition of 1.2 equivalents of a primary or secondary amine or alcohol in the presence of 2 equivalents of sodium hydride afforded the highly substituted and functionalized aziridine derivatives **2**. Under these conditions, it can be assumed that methoxide is the predominant base. The optimal reagent concentration was determined to be 0.2 M, since higher concentrations led to the precipitation of reaction intermediates and lower yields.

**Figure 1:**
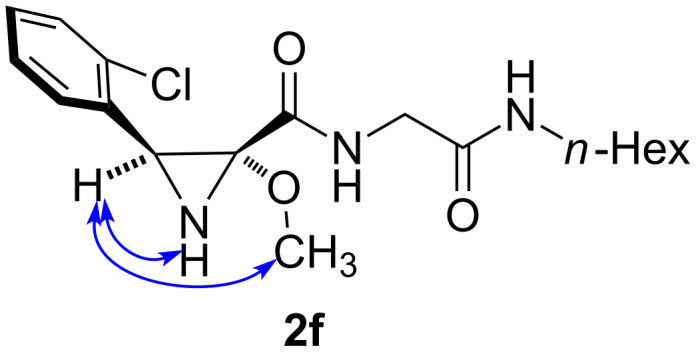
NOE effects in compound **2f**.

The scope of the reaction was investigated and the results are summarized in Table 1. Good yields were obtained independently from the electronic nature of the aryl ring. Bulky R^1^ aryl groups were well tolerated, as shown by the presence of *ortho*- substituents in many of the examples (Table 1, entries 3–11). Both primary (Table 1, entries 1, 3, 4, 5, 6, 9, and 11) and secondary amines (Table 1, entries 2 and 7) could be employed as nucleophiles, and the use of oxygen nucleophiles (methanol) was also possible (Table 1, entries 8 and 10). Nevertheless, steric limitations existed in the latter case, since the reaction did not tolerate the use of bulkier alcohols such as ethanol or propanol, which led only to recovered starting materials. In all cases, the reaction proceeded with full diastereoselectivity, affording the product with a *cis* relationship between the aryl substituent and the peptide side chain as shown by NOE studies, which are summarized in Figure 1 for compound **2f**. One example of a reaction leading to an alkyl substituent at the aziridine C-3 position (R^1^ = Et) was attempted, but the yield was poor (15%) and the resulting aziridine derivative could not be completely purified.

**Table 1:** Results obtained in the aziridine synthesis.

Entry	Compound	R^1^	ZR^2^	Yield, %

1	**2a**	C_6_H_5_	*n-*BuNH	79
2	**2b**	C_6_H_5_	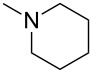	58
3	**2c**	(2,5-MeO)_2_C_6_H_3_	*n-*BuNH	75
4	**2d**	(2,5-MeO)_2_C_6_H_3_	*n-*HexNH	69
5	**2e**	2-ClC_6_H_4_	*n-*BuNH	92
6	**2f**	2-ClC_6_H_4_	*n-*HexNH	73
7	**2g**	2-ClC_6_H_4_	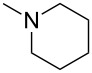	61
8	**2h**	2-ClC_6_H_4_	OMe	76
9	**2i**	2-NO_2_C_6_H_4_	*n-*BuNH	71
10	**2j**	2-NO_2_C_6_H_4_	OMe	57
11	**2k**	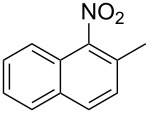	*n-*BuNH	50

**Scheme 3:**
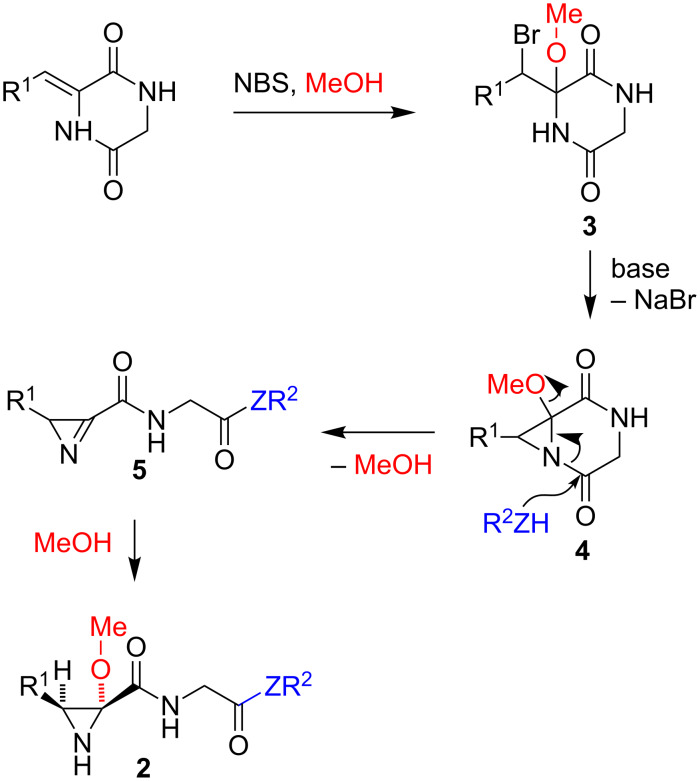
Mechanistic proposal accounting for the chemo- and diastereoselective formation of aziridines **2**.

Mechanistically, the reaction is proposed to proceed by the pathway summarized in Scheme 3. An initial bromomethoxylation of the starting materials **1** is expected to afford intermediates **3**. Although **3** may be formed as a mixture of diastereomers because of the epimerization of the initial adduct, as described in a literature precedent on a similar reaction [24], this is of no consequence for the outcome of the reaction. The next step would involve the base-promoted formation of the bicyclic aziridine **4**, where nitrogen conjugation to the adjacent carbonyl would be geometrically constrained. The associated increase in reactivity for this carbonyl would explain the complete selectivity of the diketopiperazine ring opening, with concomitant loss of a molecule of methanol, to furnish azirine **5**. Finally, addition of the methanol released in the previous step to the highly strained intermediate **5** would take place *anti* to the R^1^ substituent, explaining the observed diastereoselection. This proposal was supported by the isolation of one of its intermediates from the reaction between 3-(2,5-dimethoxybenzylidene)-2,5-piperazinedione and NBS in dioxane containing methanol at room temperature. This reaction led to the corresponding compound **3** in 75% yield, with full regioselectivity but as a 1.7:1 mixture of diastereomers. When this mixture was treated with butylamine and sodium hydride under our standard conditions, compound **2c** was isolated in 85% yield.

**Scheme 4:**
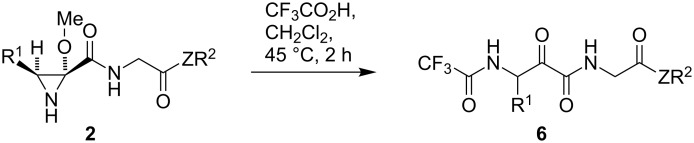
Transformation of aziridines **2** into β-trifluoroacetamido-α-ketoamides **6**.

With compounds **2** in hand, we next examined their transformation into β-amino-α-ketoamide derivatives. While the synthetic applications of aziridines have received much attention [11,25–29] there is little precedent of their opening with weak nucleophiles such as carboxylate anions [30]. However, we expected that the presence of the 2-methoxy substituent would facilitate the desired transformation. The initial study was carried out on compound **2e**, but we found that a variety of acidic conditions either failed to give any reaction at room temperature or afforded complex mixtures when heated. After some experimentation, we finally discovered that treatment of compound **2e** with trifluoroacetic acid in dichloromethane at 45 °C gave an excellent yield of the β-trifluoroacetamido-α-ketoamide **6d**. The method was then applied to a range of representative aziridines **2**, allowing the synthesis of the corresponding compounds **6** in excellent yields (Scheme 4 and Table 2).

**Table 2:** Results of the synthesis of β-trifluoroacetamido-α-ketoamides **6**.

Entry	Compound	R^1^	ZR^2^	Yield, %

1	**6a**	C_6_H_5_	*n-*BuNH	90
2	**6b**	(2,5-MeO)_2_C_6_H_3_	*n-*BuNH	87
3	**6c**	(2,5-MeO)_2_C_6_H_3_	*n-*HexNH	92
4	**6d**	2-ClC_6_H_4_	*n-*BuNH	97
5	**6e**	2-ClC_6_H_4_	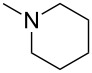	93
6	**6f**	2-ClC_6_H_4_	OMe	98

**Scheme 5:**
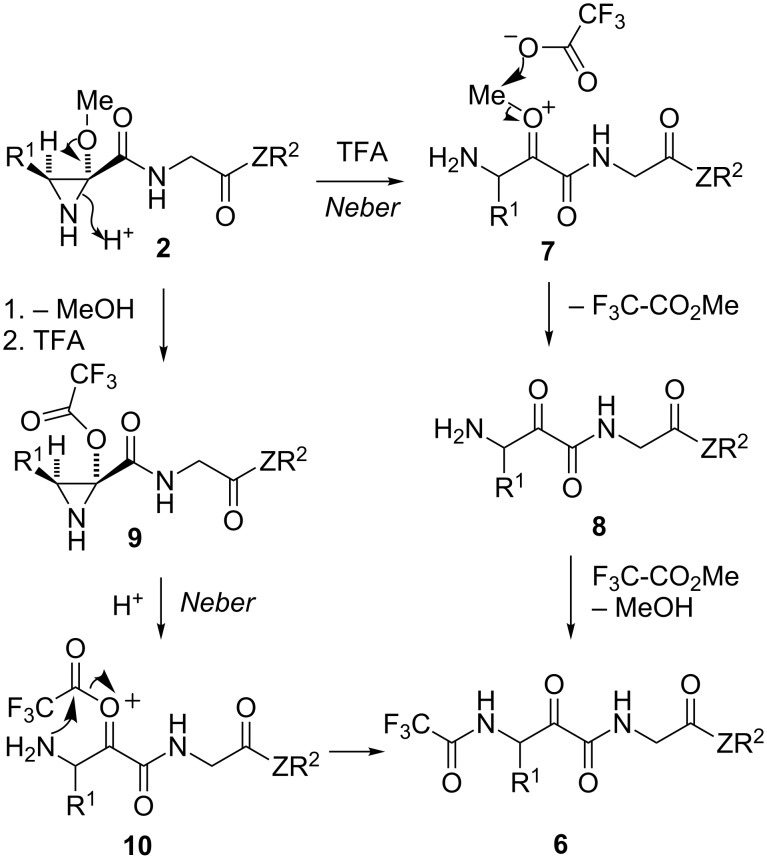
Two mechanistic proposals explaining the formation of compounds **6**.

The formation of **6** can be explained by the mechanism summarized in Scheme 5. The acid-promoted opening of the hemiaminal ether function existent in aziridine derivatives **2** because of the presence of the 2-methoxy substituent would furnish oxonium species **7** via a Neber reaction. Its O-demethylation by the trifluoroacetate anion would yield compound **8**, whose amino group would finally be trifluoroacetylated by the methyl trifluoroacetate liberated in the previous step, to give **6**. Alternatively, loss of a molecule of methanol from starting compound **2** would lead to intermediate **5** (see Scheme 3), whose reaction with trifluoroacetic acid would furnish **9**. Neber-type chemistry would afford the oxonium species **10**, which would finally be transformed into the observed product **6** by intramolecular transfer of the trifluoroacetyl group.

**Scheme 6:**
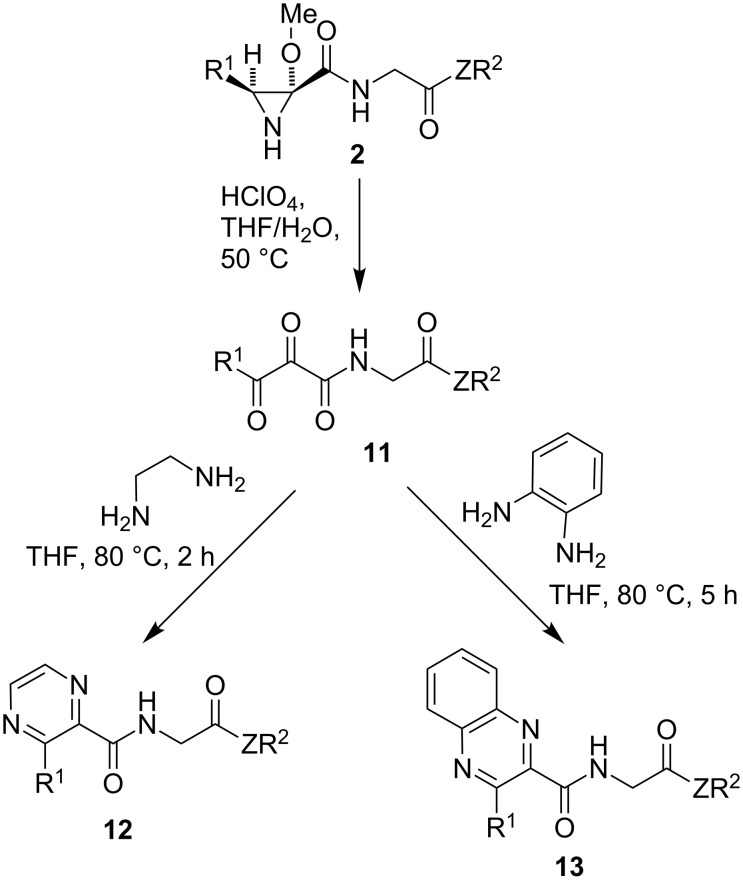
Transformation of aziridines **2** into vicinal tricarbonyl compounds **11** and transformation of the latter into nitrogen heterocycles **12** and **13**, containing a peptide side chain.

Finally, bearing in mind the interest of 1,2,3-tricarbonyl compounds (vicinal tricarbonyl compounds, VCTs) both as structural fragments of protease inhibitors [31] and synthetic intermediates [32–33], we also explored briefly their direct preparation from compounds **2**, together with their application to the synthesis of nitrogen heterocycles bearing a peptide side chain. We discovered that treatment of representative aziridines **2** with perchloric acid in a THF–water reaction medium led to the isolation of the corresponding compounds **11**, which were then transformed into pyrazines **12** and quinoxalines **13** via straightforward cyclocondensation reactions with ethylenediamine and *o*-phenylenediamine, respectively (Scheme 6 and Table 3).

**Table 3:** Results of the synthesis of vicinal tricarbonyl compounds **11** and the nitrogen heterocycles **12** and **13** arising from them.

Entry	Compound	R^1^	ZR^2^	Yield, %

1	**11a**	2-ClC_6_H_4_	*n-*BuNH	67
2	**11b**	(2,5-MeO)_2_C_6_H_3_	*n-*BuNH	64^a^
3	**12a**	2-ClC_6_H_4_	*n-*BuNH	64
4	**12b**	(2,5-MeO)_2_C_6_H_3_	*n-*BuNH	67
5	**13a**	2-ClC_6_H_4_	*n-*BuNH	61
6	**13b**	(2,5-MeO)_2_C_6_H_3_	*n-*BuNH	74
7	**13c**	2-ClC_6_H_4_	OMe	74

^a^Two equivalents of perchloric acid were required in this case. The normal reaction conditions (1 equiv HClO_4_) led to 13% yield of **11b** and 52% of compound **14b** (see below the mechanistic discussion associated to Scheme 7).

**Scheme 7:**
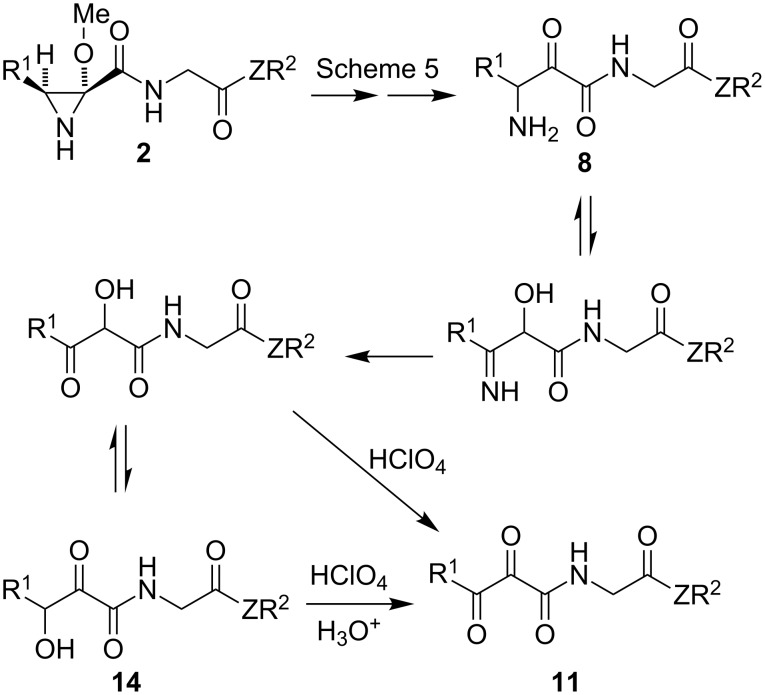
Mechanistic proposal for the transformation of aziridines **2** into compounds **11**.

The transformation of **2** into **11** can be explained by the mechanism summarized in Scheme 7, starting with the formation of the previously mentioned intermediate **8**, followed by hydrolysis of its imino tautomer to give **14** and a final oxidation by perchloric acid. One of the proposed intermediates (compound **14b**) was isolated by lowering the amount of perchloric acid, and was transformed into **11b** under the general conditions employed for the preparation of **11** from **2**.

## Conclusion

We have developed an efficient, mechanistically novel entry into polysubstituted, functionalized aziridines. Our method is based on a sequential three-component reaction initiated by bromomethoxylation of the exocyclic double bond in readily available 3-arylmethylene-2,5-piperazinediones, followed by a domino process involving three consecutive nucleophilic attacks that achieve regioselective opening of the diketopiperazine ring and diastereoselective generation of the aziridine ring. The protocol described here proceeds under mild and functional group-tolerant conditions, allowing the synthesis of *N*-unprotected, trisubstituted aziridines bearing a peptide side chain. The synthetic application of these aziridines was demonstrated by their one-pot transformation into β-trifluoroacetamido-α-ketoamides, thus providing a fast and efficient access to an important pharmacophore in the design of enzyme inhibitors. Furthermore, the aziridines could also be transformed into α,β-diketoamides, which were suitable precursors for nitrogen heterocycles bearing a peptide side chain.

## Supporting Information

File 1 Experimental section, copies of ^1^H NMR, ^13^C NMR and ESIMS spectra of all new compounds.
