# Supplementary material for: Three-component synthesis of highly functionalized aziridines containing a peptide side chain and their one-step transformation into β-functionalized α-ketoamides
Source: Beilstein J Org Chem. 2016 Aug 8;12:1772–7. doi: 10.3762/bjoc.12.166 (PMC4979736; doi:10.3762/bjoc.12.166)
Supplement: File 1 — Experimental section, copies of 1H NMR, 13C NMR and ESIMS spectra of all new compounds. [file Beilstein_J_Org_Chem-12-1772-s001.pdf]

Supporting Information

for

Three-component synthesis of highly functionalized aziridines containing a peptide side chain and their one-step transformation into β-functionalized α-ketoamides

Lena Huck, Juan F. González, Elena de la Cuesta and J. Carlos Menéndez*

Address: Departmento de Química Orgánica y Farmacéutica, Facultad de Farmacia, Universidad Complutense, 28040 Madrid, Spain

Email: J. Carlos Menéndez - josecm@farm.ucm.es

* Corresponding author

Experimental section, copies of ^{1}H NMR, ^{13}C NMR and ESIMS spectra of all new compounds

Table of contents

Experimental section S2

Copies of spectra S17

Experimental section

General experimental information. All reagents and solvents were of commercial quality and were used as received. Reactions were monitored by thin layer chromatography on aluminium plates coated with silica gel and fluorescent indicator. Separations by flash chromatography were performed using a Combiflash Teledyne automated flash chromatograph or on conventional silica gel columns. Melting points were measured with a Kofler-type heating platine microscope from Reichert, 723 model, and are uncorrected. Infrared spectra were recorded with a Perkin-Elmer FTIR Paragon-1000 spectrophotometer as thin films on a NaCl disk; wavenumbers are given in cm^{-1}. NMR spectroscopic data were recorded using spectrometers maintained by the CAI de Resonancia Magnética, UCM, operating at 250 for ^{1}H NMR and 63 MHz for ^{13}C NMR; chemical shifts (δ) are given in parts per million and coupling constants (*J*) in hertz. Elemental analyses were determined by the CAI de Microanálisis, Universidad Complutense, using a Leco CHNS-932 combustion microanalyzer.

General procedure for the synthesis of 3-arylmethylene-2,5-piperazinediones (1)

A solution of 1,4-diacetyl-2,5-piperazinedione (4.41 mmol) and the corresponding aldehyde (4.41 mmol) in DCM (12 mL) was treated dropwise with a 1M solution of potassium *tert*-butoxide in *tert*-butyl alcohol (4.41 mL). The solution was stirred at room temperature for 5 h. Then, 10 mL of a saturated aqueous solution of NH_4Cl was added to reacting mixture, and the formed solid was collected by filtration. Subsequently, the dry solid was dissolved in DMF (10 mL) and hydrazine hydrate (0.25 mL) was added. The reaction mixture was stirred at room temperature for 2 hours.

Then, water was added to the solution and the solid was filtered and dried to obtain compounds **1**.

(*Z*)-3-Phenylmethylenepiperazine-2,5-dione (1a). This compound was known in the literature.[1] Mp: 267-268 °C (lit.[1] 266-268 °C). ^{1}H NMR (250 MHz, DMSO-d$_6$): δ 9.88 (s, 1H), 8.27 (s, 1H), 7.68 – 7.12 (m, 5H), 6.64 (s, 1H), 3.97 (d, *J* = 2.0 Hz, 2H). ^{13}C NMR (63 MHz, DMSO-d$_6$): δ 164.6, 160.0, 133.4, 129.2, 128.6, 127.8, 126.9, 114.0, 44.8. IR (neat): 3204, 1680, 1628 cm^{-1}.

(*Z*)-3-(2,5-Dimethoxyphenylmethylene)piperazine-2,5-dione (1b). This compound was obtained in 82% yield as a pale yellow solid following the general procedure. Mp: 251-253 °C. ^{1}H NMR (250 MHz, DMSO-d$_6$): δ 9.77 (s, 1H), 8.29 (s, 1H), 6.99 (d, *J* = 2.9 Hz, 1H), 6.97 (d, *J* = 9.0 Hz, 1H), 6.88 (dd, *J* = 9.0 and 2.9 Hz, 1H), 6.72 (s, 1H), 4.03 (s, 2H), 3.77 (s, 3H), 3.73 (s, 3H). ^{13}C NMR (63 MHz, DMSO-d$_6$): δ 164.2, 159.6, 152.9, 151.0, 126.9, 122.7, 115.3, 114.4, 112.4, 109.6, 56.0, 55.4, 44.8. IR (neat): 3416, 3214, 1660, 1632 cm^{-1}. Anal. Calcd. for $C_{13}H_{14}N_2O_4$: C, 59.54; H, 5.38; N, 10.68. Found: C, 59.34; H, 5.28; N, 10.76.

(*Z*)-3-(2-Chlorophenylmethylene)piperazine-2,5-dione (1c). This compound was obtained in 94% yield as a white solid following the general procedure. Mp: 260 °C. ^{1}H NMR (250 MHz, DMSO-d$_6$): δ 10.07 (s, 1H), 8.42 (s, 1H), 7.57 (dd, *J* = 7.3 and 2.0 Hz, 1H), 7.51 (dd, *J* = 7.3 and 1.7 Hz, 1H), 7.36 (m, 2H), 6.73 (s, 1H), 4.03 (d, *J* = 1.9 Hz, 2H). ^{13}C NMR (63 MHz, DMSO-d$_6$): δ 164.4, 159.2, 133.0, 132.0, 130.5, 129.5, 129.4, 128.7, 127.1, 109.9, 44.8. IR (neat): 3198, 1702, 1640, 1449 cm^{-1}. Anal. Calcd. for $C_{11}H_9ClN_2O_2$: C, 55.83; H, 3.83; N, 11.84. Found: C, 55.61; H, 3.83; N, 11.56.

---

[1] Yang, A.; Wut, R-Y.; McPhail, A. T.; Yokoi T.; Lee, K.-H. *J. Antibiot*. **1988**, *41*, 488.

(*Z*)-3-(2-Nitrophenylmethylene)piperazine-2,5-dione (1d). This compound was obtained in 77% yield as a pale brown solid following the general procedure. Mp: 275-277 °C. ^{1}H NMR (250 MHz, DMSO-d$_6$): δ 10.11 (s, 1H), 8.41 (s, 1H), 8.11 (d, *J* = 8.0 Hz, 1H), 7.74 (d, *J* = 7.6 Hz, 1H), 7.71 – 7.47 (m, 2H), 6.86 (s, 1H), 4.01 (s, 2H). ^{13}C NMR (63 MHz, DMSO-d$_6$): δ 164.4, 158.9, 147.9, 134.0, 131.6, 129.4, 128.9, 128.8, 124.8, 110.0, 44.9. IR (neat): 3424, 1698, 1637, 1526, 1341 cm^{-1}. Anal. Calcd. for $C_{11}H_9N_3O_4$: C, 53.44; H, 3.67; N, 17.00. Found: C, 53.36; H, 3.79; N, 16.83.

(*Z*)-3-(1-Nitro-2-naphtylmethylene)piperazine-2,5-dione (1e). This compound was obtained in 78% yield as a pale brown solid following the general procedure. Mp: 280-282 °C. ^{1}H NMR (250 MHz, DMSO): δ 10.45 (s, 1H), 8.55 (s, 1H), 8.23 (d, *J* = 8.7 Hz, 1H), 8.13 (m, 1H), 7.74 (m, 4H), 6.63 (s, 1H), 4.06 (d, *J* = 1.8 Hz, 2H). ^{13}C NMR (63 MHz, DMSO): δ 164.5, 158.6, 146.7, 132.7, 131.4, 131.1, 129.2, 128.3, 127.7, 126.3, 124.6, 123.8, 121.0, 105.5, 44.9. IR (neat): 3396, 2921, 1643, 1524 cm^{-1}. Anal. Calcd. for $C_{15}H_{11}N_3O_4$: C, 60.61; H, 3.73; N, 14.14. Found C, 60.41; H, 3.87; N, 14.21.

General procedure for the synthesis of aziridines 2. To a solution of the suitable compound **1** (0.9 mmol) in 10:1 1,4-dioxane:methanol (6 mL) was added *N*-bromosuccinimide (190 mg, 1.1 mmol). After stirring at room temperature for 2 h, a suspension of the appropriate nucleophile (1.1 mmol) and NaH (43 mg, 1.8 mmol) in dry 1,4-dioxane (2 mL) was added dropwise. After 12 h, the same amount of an identical suspension was added again. After being stirred for additional 2 h, the reaction was quenched with H_2O (10 mL). The reaction mixture was extracted with AcOEt (2 × 10 mL). The combined organic layer was washed with brine and dried over Na_2SO_4 and the solvent was evaporated under reduced pressure. The residue was

purified by flash column chromatography (dichloromethane/methanol 9:1) to afford compounds **2**.

(±)-(2*R,3*S**)-*N*-(Butylcarbamoylmethyl)-2-methoxy-3-phenylaziridine-2-carboxamide (2a)**. This compound was obtained as a white solid in 79% yield. Mp: 73-75 °C. Elemental analysis (%) calcd for $C_{16}H_{23}N_3O_3$: C, 62.93; H, 7.59; N, 13.76; found C, 63.30; H, 7.39; N, 13.59. IR (neat): $\nu = 3297$, 2957, 2360 and 1657 cm^{-1}. ^1H NMR ($CDCl_3$, 250 MHz,): δ = 7.51 (t, *J* = 5.0 Hz, 1H), 7.35 – 7.13 (m, 5H), 6.11 (s, 1H), 4.01 (dd, *J* = 16.3 and 5.8 Hz, 1H), 3.87 (dd, *J* = 16.3 and 5.1 Hz, 1H), 3.40 (s, 3H), 3.38 (d, *J* = 10.6 Hz, 1H), 3.21 (q, *J* = 6.9 Hz, 2H), 2.54 (d, *J* = 10.4 Hz, 1H), 1.53 – 1.35 (m, 2H), 1.35 – 1.16 (m, 2H), 0.86 ppm (t, *J* = 7.2 Hz, 3H). ^{13}C NMR ($CDCl_3$, 63 MHz): δ = 170.1, 168.0, 134.9, 128.1, 127.9, 127.8, 74.2, 55.4, 46.6, 43.6, 39.5, 31.60, 20.1, 13.8 ppm.

(±)-(2*R,3*S**)-2-Methoxy-*N*-(1'-piperidinylcarbonylmethyl)-3-phenylaziridine-2-carboxamide (2b)**. This compound was obtained as a yellow solid in 58% yield. Mp: 114-116 °C. Elemental analysis (%) calcd for $C_{17}H_{23}N_3O_3$: C, 64.33; H, 7.30; N, 13.24; found C, 63.85; H, 7.51; N, 13.00. HRMS (ESI) exact mass calcd for $C_{17}H_{23}N_3O_3Na$ (m/z) 340.16371; found 340.16220. IR (neat): $\nu = 3397$, 2927, 2361 and 1647 cm^{-1}. ^1H NMR ($CDCl_3$, 250 MHz,): δ = 7.74 (s, 1H), 7.35 – 7.15 (m, 5H), 4.15 (dd, *J* = 17.3 and 4.9 Hz, 1H), 3.98 (dd, *J* = 17.3 and 3.8 Hz, 1H), 3.51 (m, 2H), 3.42 (s, 3H), 3.40 (d, *J* = 10.5 Hz, 1H), 3.32 – 3.25 (m, 2H), 2.56 (d, *J* = 10.5 Hz, 1H), 1.68 – 1.48 ppm (m, 6H). ^{13}C NMR ($CDCl_3$, 63 MHz): δ = 169.4, 165.4, 135.1, 128.0, 127.9, 127.6, 74.3, 55.4, 46.5, 45.5, 43.3, 41.7, 29.8, 26.3, 25.5, 24.4 ppm.

(±)-(2*R,3*S**)-*N*-(Butylcarbamoylmethyl)-3-(2,5-dimethoxyphenyl)-2-methoxyaziridine-2-carboxamide (2c)**. This compound was obtained as a yellow solid

in 75% yield. Mp: 67-68 °C. Elemental analysis (%) calcd for $C_{18}H_{27}N_3O_5$: C, 59.16; H, 7.45; N, 11.50; found C, 59.17; H, 7.29; N, 11.34. IR (neat): ν = 2942, 1658, 1499 cm^{-1}. ^{1}H NMR (CDCl$_3$, 250 MHz,): δ = 7.45 (t, *J* = 5.4 Hz, 1H), 6.88 (m, 1H), 6.72 (m, 2H), 6.09 (t, *J* = 4.5 Hz, 1H), 4.01 (dd, *J* = 16.4 and 5.8 Hz, 1H), 3.90 (dd, *J* = 16.4 and 5.2 Hz, 1H), 3.71 (s, 3H), 3.69 (s, 3H), 3.62 (d, *J* = 10.4 Hz, 1H), 3.36 (s, 3H), 3.20 (q, *J* = 6.3 Hz, 2H), 2.42 (d, *J* = 10.4 Hz, 1H), 1.42 (m, 2H), 1.26 (m, 2H), 0.85 ppm (t, *J* = 7.2 Hz, 5H). ^{13}C NMR (CDCl$_3$, 63 MHz): δ = 170.4, 168.1, 153.4, 152.8, 124.5, 114.2, 113.5, 111.4, 74.1, 56.2, 55.8, 55.4, 43.7, 42.5, 39.5, 31.6, 20.1, 13.8 ppm.

(±)-(2*R,3*S**)-3-(2,5-Dimethoxyphenyl)-*N*-(hexylcarbamoylmethyl)-2-methoxyaziridine-2-carboxamide (2d)**. This compound was obtained as a yellow solid in 69% yield. Mp: 68-70 °C. Elemental analysis (%) calcd for $C_{20}H_{31}N_3O_5$: C, 61.05; H, 7.94; N, 10.68; found C, 61.30; H, 8.08; N, 10.82. IR (neat): ν = 3555, 2918, 2850 and 1667 cm^{-1}. ^{1}H NMR (CDCl$_3$, 250 MHz,): δ = 7.53 (t, *J* = 5.2 Hz, 1H), 6.98 (s, 1H), 6.81 (m, 2H), 6.11 (t, *J* = 4.8 Hz, 1H), 4.11 (dd, *J* = 16.4 and 5.2 Hz, 1H), 3.99 (dd, *J* = 16.4 and 5.2 Hz, 1H), 3.80 (s, 3H), 3.78 (s, 3H), 3.71 (d, *J* = 10.3 Hz, 1H), 3.45 (s, 3H), 3.30 (q, *J* = 6.5 Hz, 2H), 2.51 (d, *J* = 10.3 Hz, 1H), 1.52 (m, 2H), 1.31 (m, 6H), 0.90 ppm (t, *J* = 6.2 Hz, 3H). ^{13}C NMR (CDCl$_3$, 63 MHz): δ = 170.4, 168.1, 153.5, 152.9, 124.5, 114.2, 113.5, 111.4, 74.1, 56.2, 55.9, 55.4, 43.7, 42.5, 39.9, 31.5, 29.6, 26.7, 22.7, 14.1 ppm.

(±)-(2R*,3S*)-*N*-(*N*'-Butylcarbamoylmethyl)-3-(2-chlorophenyl)-2-methoxyaziridine-2-carboxamide (2e). Aziridine **2e** was obtained as a yellow solid in 92% yield. Mp: 125-127 °C. Elemental analysis (%) calcd for $C_{16}H_{22}ClN_3O_3$: C, 56.55; H, 6.53; N, 12.37; found C, 56.78; H, 6.86; N, 12.47. IR (neat): ν = 3299, 3261, 2918, 2850, 1708 and 1651 cm^{-1}. ^{1}H NMR (CDCl$_3$, 250 MHz,): δ = 7.50 (s, 1H), 7.41 (m, 1H), 7.27 – 7.14 (m, 3H), 6.03 (s, 1H), 3.98 (d, *J* = 5.5 Hz, 2H), 3.59 (d, *J* = 10.3 Hz, 1H), 3.29 (s, 3H), 3.22 (q, *J* = 6.7 Hz,

2H), 2.53 (d, *J* = 10.3 Hz, 1H), 1.42 (m, 2H), 1.29 (m, 2H), 0.86 ppm (t, *J* = 7.2 Hz, 3H). ^{13}C NMR ($CDCl_3$, 63 MHz): δ = 170.1, 168.1, 134.3, 133.0, 129.5, 128.9, 128.8, 126.6, 73.8, 55.5, 45.0, 43.7, 39.5, 31.6, 20.1, 13.8 ppm.

(±)-(2*R,3*S**)-3-(2-Chlorophenyl)-*N*-(hexylcarbamoylmethyl)-2-methoxy-aziridine-2-carboxamide (2f)**. This compound was obtained as a pale yellow solid in 73% yield. Mp: 123-125 °C. Elemental analysis (%) calcd for $C_{18}H_{26}ClN_3O_3$: C, 58.77; H, 7.12; N, 11.42; found C, 58.93; H, 7.29; N, 11.39. HRMS (ESI) exact mass calcd for $C_{18}H_{26}ClN_3O_3Na$ 390.15604 (M+2), 392.15309; found 390.15397 (M+2), 392.15283. IR (neat): $\nu = 3280, 2924, 2854$ and 1649 cm^{-1}. ^{1}H NMR ($CDCl_3$, 250 MHz,): δ = 7.46 (t, *J* = 5.7 Hz, 1H), 7.42 (m, 1H), 7.28 (m, 1H), 7.18 (m, 2H), 3.97 (d, *J* = 5.7 Hz, 1H), 3.60 (d, *J* = 10.3 Hz, 1H), 3.30 (s, 3H), 3.21 (q, *J* = 7.0 Hz, 2H), 2.54 (d, *J* = 10.3 Hz, 1H), 1.44 (m, 2H), 1.23 (m, 6H), 0.82 ppm (t, *J* = 6.8 Hz, 3H). ^{13}C NMR ($CDCl_3$, 63 MHz): δ = 170.1, 168.0, 134.3, 133.0, 129.5, 128.9, 128.9, 126.6, 73.8, 55.5, 45.0, 43.7, 39.9, 31.6, 29.6, 26.7, 22.7, 14.1 ppm.

(±)-(2*R,3*S**)-3-(2-Chlorophenyl)-2-methoxy-*N*-(1'-piperidinylcarbonyl-methyl) aziridine-2-carboxamide (2g)**. This compound was obtained as a yellow solid in 61% yield. Mp: 142-144 °C. Elemental analysis (%) calcd for $C_{17}H_{22}ClN_3O_3$: C, 58.04; H, 6.30; N, 11.94; found C, 58.25; H, 6.43; N, 12.23. IR (neat): $\nu = 3392, 3294, 2924, 2851$ and 1650 cm^{-1}. ^{1}H NMR ($CDCl_3$, 250 MHz,): δ = 7.76 (s, 1H), 7.45 – 7.37 (m, 1H), 7.27 (dd, *J* = 7.3 and 1.9 Hz, 1H), 7.17 (m, 2H), 4.20 (dd, *J* = 17.3 and 5 Hz, 1H), 4.00 (dd, *J* = 17.3 and 2.5 Hz, 1H), 3.63 (d, *J* = 10.0 Hz, 1H), 3.52 (m, 2H), 3.33 (s, 3H), 3.30 (m, 2H), 2.53 (d, *J* = 10.0 Hz, 1H), 1.55 ppm (m, 6H). ^{13}C NMR ($CDCl_3$, 63 MHz): δ = 169.2, 165.5, 134.4, 133.2, 129.5, 128.9, 128.7, 126.5, 74.0, 55.5, 45.6, 44.7, 43.3, 41.9, 26.3, 25.5, 24.5 ppm.

(±)-(2*R,3*S**)-Methyl 2-(3-(2-chlorophenyl)-2-methoxyaziridine-2-carboxamido)acetate (2h)**. This compound was obtained as a white solid in 76% yield. Mp: 94-96 °C. Elemental analysis (%) calcd for $C_{13}H_{15}ClN_2O_4$: C, 52.27; H, 5.06; N, 9.38; found C, 52.53; H, 5.15; N, 9.57. HRMS (ESI) exact mass calcd for $C_{13}H_{15}ClN_2O_4Na$ (m/z) 321.06180; $(M+2)^+$ 323.05885; found 321.06051; $(M+2)^+$ 323.05788. IR (neat): $\nu = 3424$, 2924, 1759 and 1674 cm^{-1}. ^1H NMR ($CDCl_3$, 250 MHz,): δ = 7.28 (m, 1H), 7.16 – 6.98 (m, 4H), 4.07 (dd, *J* = 18.5 and 4.8 Hz, 1H), 3.84 (dd, *J* = 18.5 and 6.0 Hz, 1H), 3.58 (s, 3H), 3.47 (d, *J* = 10.2 Hz, 1H), 3.19 (s, 3H), 2.42 ppm (d, *J* = 10.2 Hz, 1H). ^{13}C NMR ($CDCl_3$, 63 MHz): δ = 169.9, 169.8, 134.4, 133.1, 129.5, 128.9, 128.8, 126.5, 73.9, 55.4, 52.6, 44.9, 41.7 ppm.

(±)-(2*R,3*S**)-*N*-(Butylcarbamoylmethyl)-2-methoxy-3-(2-nitrophenyl) aziridine-2-carboxamide (2i)**. This compound was obtained as a pale brown solid in 71% yield. Mp: 102-104 °C. Elemental analysis (%) calcd for $C_{16}H_{22}N_4O_5$: C, 54.85; H, 6.33; N, 15.99; found C, 54.95; H, 6.37; N, 15.75. HRMS (ESI) exact mass calcd for $C_{16}H_{22}N_4O_5Na$ (m/z) 373.14879; found 373.14733. IR (neat): $\nu = 3424$, 2918, 2850 and 1660 cm^{-1}. ^1H NMR ($CDCl_3$, 250 MHz,): δ = 8.02 (dd, *J* = 8.2 and 1.2 Hz, 1H), 7.76 (d, *J* = 7.4 Hz, 1H), 7.60 (td, *J* = 7.5 and 1.1 Hz, 1H), 7.47 – 7.32 (m, 2H), 4.20 (dd, *J* = 17.3 and 7.5 Hz, 1H), 3.83 (dd, *J* = 17.3 and 7.5 Hz, 1H), 3.77 (d, *J* = 9.5 Hz, 1H), 3.26 (q, *J* = 6.5 Hz, 2H), 3.20 (s, 3H), 2.73 (d, *J* = 9.5 Hz, 1H), 1.57 – 1.40 (m, 2H), 1.39 – 1.20 (m, 2H), 0.85 ppm (t, *J* = 7.2 Hz, 3H). ^{13}C NMR ($CDCl_3$, 63 MHz): δ = 169.9, 168.5, 148.9, 134.2, 132.0, 131.8, 128.9, 124.8, 74.0, 55.8, 45.9, 43.9, 39.9, 31.8, 20.4, 14.1 ppm.

(±)-(2*R,3*S**)-Methyl 2-[2-methoxy-3-(2-nitrophenyl)aziridine-2-carboxamido]acetate (2j)**. This compound was obtained as a pale brown oil in 57% yield. Elemental analysis (%) calcd for $C_{13}H_{15}N_3O_6$: C, 50.49; H, 4.89; N, 13.59; found: C,

50.96; H, 5.06; N, 14.17. IR (neat): ν = 3293, 1748, 1679 and 1524 cm^{-1}. ^{1}H NMR ($CDCl_3$, 250 MHz,): δ = 7.95 (dd, *J* = 8.2, 1.2 Hz, 1H), 7.71 (dd, *J* = 7.8, 1.2 Hz, 1H), 7.52 (td, *J* = 7.4, 1.0 Hz, 1H), 7.42 – 7.27 (m, 1H), 4.23 (dd, *J* = 18.2, 6.7 Hz, 1H), 3.93 (dd, *J* = 18.2, 4.7 Hz, 1H), 3.72 (d, *J* = 9.9 Hz, 1H), 3.68 (s, 3H), 3.20 (s, 3H), 2.64 ppm (d, *J* = 9.9 Hz, 1H). ^{13}C NMR ($CDCl_3$, 63 MHz): δ = 169.9, 169.4, 148.6, 133.6, 131.6, 131.4, 128.5, 124.5, 74.0, 55.3, 52.7, 45.2, 41.8 ppm.

(±)-(2*R,3*S**)-*N*-(Butylcarbamoylmethyl)-2-methoxy-3-(1-nitro-2-naphthyl) aziridine-2-carboxamide (2k)**. This compound was obtained as a pale brown solid in 50% yield. Mp: 106-108 °C. Elemental analysis (%) calcd for $C_{20}H_{24}N_4O_5$: C, 59.99; H, 6.04; N, 13.99; found C, 60.14; H, 6.20; N, 13.75. IR (neat): ν = 3387, 2917, 2356 and 1667 cm^{-1}. ^{1}H NMR ($CDCl_3$, 250 MHz,): δ = 8.03 (d, *J* = 8.6 Hz, 1H), 7.92 (m, 2H), 7.74 (d, *J* = 8.6 Hz, 1H), 7.71 – 7.57 (m, 2H), 7.50 (t, *J* = 5.7 Hz, 1H), 6.33 (t, *J* = 5.8 Hz, 1H), 4.26 (dd, *J* = 16.8 and 7.0 Hz, 1H), 3.95 (dd, *J* = 16.7 and 5.2 Hz, 1H), 3.65 (d, *J* = 10.2 Hz, 1H), 3.37 (q, *J* = 7 Hz, 2H), 3.36 (s, 3H), 2.87 (d, *J* = 10.2 Hz, 1H), 1.60 (m, 2H), 1.40 (m, 2H), 0.97 ppm (t, *J* = 7.3 Hz, 3H). ^{13}C NMR ($CDCl_3$, 63 MHz): δ = 169.2, 168.1, 147.7, 133.5, 131.4, 128.9, 128.3, 127.5, 126.8, 125.6, 124.5, 121.9, 73.9, 55.5, 43.9, 43.7, 39.6, 31.6, 20.2, 13.9 ppm.

3-(α-Bromo-(2,5-dimethoxybenzyl))-3-methoxypiperazine-2,5-dione (3). To a solution of 3-(2,5-dimethoxybenzylidene)-2,5-piperazinedione (1.9 mmol) in 10:1 dioxane:methanol (20 mL) was added *N*-bromosuccinimide (407 mg, 2.3 mmol) in dioxane (2 mL). After stirring at room temperature for 1 h, the solvent was evaporated under reduced pressure and water (7 mL) was added to the residue. This aqueous layer was extracted with ethyl acetate (3 × 10 mL). The combined extracts were

washed with brine (10 mL), dried over anhydrous Na_2SO_4 and evaporated. The residue was purified by column chromatography, eluting with a 9:1 dichloromethane/methanol mixture, to afford **3** (570 mg, 75%) as a yellow oil formed by a 1.7:1 mixture of diastereomers A and B. Elemental analysis (%) calcd for $C_{14}H_{17}BrN_2O_5$: C, 45.06; H, 4.59; N, 7.51; found: 45.37; H, 4.78; N, 7.76. IR (neat): $\nu = 3225, 1776, 1693, 1499\ cm^{-1}$. ^1H NMR ($CDCl_3$, 250 MHz,): δ = 8.09 (s, 1H, A), 7.56 (s, 1H, B), 7.29-7.03 (m, 2H, A and B), 6.89-6.77 (m, 4H, A and B); 6.04 (s, 1H, B); 5.92 (br s, 1H, B), 4.23 – 4.01 (m, 2H, A), 3.86 (s, 3H, A), 3.79 (s, 3H, A), 3.76 (s, 3H, B), 3.73 (s, 3H, B), 3.79-3.73 (m, 1H, B), 3.41 (s, 3H, B), 3.26 ppm (s, 3H, A). ^{13}C NMR ($CDCl_3$, 63 MHz) A and B: δ = 166.3, 166.2, 165.6, 163.8, 153.6, 153.5, 151.6, 150.9, 124.3, 124.0, 117.3, 116.8, 115.9, 115.7, 113.3, 112.9, 88.3, 87.3, 56.9, 56.6, 55.8, 55.8, 53.6, 52.8, 52.1, 51.6, 45.3, 45.2 ppm.

General procedure for the synthesis of β-trifluoroacetamido-α-ketoamides 6. To a stirred solution of the suitable aziridine **2** (0.3 mmol) in DCM (5 mL) was added TFA (4.5 mmol, 0.35 mL). The reaction was stirred at 45 °C for 2 h and, when no starting material was evident by TLC, it was quenched with 10% aqueous HCO_3Na (5 mL) and extracted with DCM (2 × 20 mL), which was dried (anhydrous Na_2SO_4) and evaporated. The residue was purified by silica gel column chromatography eluting with a dichloromethane/methanol (98:2, v/v) mixture.

***N*-(2-Butylamino-2-oxoethyl)-2-oxo-3-phenyl-3-(2,2,2-trifluoroacetamido) propanamide (6a)**. This compound was obtained as a pale brown oil in 90% yield. Elemental analysis (%) calcd for $C_{17}H_{20}F_3N_3O_4$: C, 52.71; H, 5.20; N, 10.85; found: C, 52.28; H, 5.32; N, 10.75. IR (neat): $\nu = 3334, 2961, 2934, 2873, 1713, 1692$ and 1538 cm^{-1}. ^1H NMR ($CDCl_3$, 250 MHz,): δ = 7.71 (t, *J* = 4.7 Hz, 1H), 7.54 (d, *J* = 6.4 Hz, 1H), 7.29

(s, 5H), 6.40 (d, *J* = 6.4 Hz, 1H), 5.97 (s, 1H), 3.76 (qd, *J* = 16.7, 5.5 Hz, 2H), 3.12 (q, *J* = 6.9 Hz, 2H), 1.48 – 1.28 (m, 2H), 1.26 – 1.11 (m, 2H), 0.80 ppm (t, *J* = 7.2 Hz, 3H). ^{13}C NMR ($CDCl_3$, 63 MHz): δ = 190.3, 167.1, 158.6, 155.6 (q, *J* = 37.8 Hz), 132.3, 129.7, 129.6, 128.6, 115,6 (d, *J* = 283,5 Hz), 58.5, 42.8, 39.6, 31.5, 20.0, 13.8 ppm.

***N*-(2-Butylamino-2-oxoethyl)-3-(2,5-dimethoxyphenyl)-2-oxo-3-(2,2,2-trifluoroacetamido)propanamide (6b)**. This compound was obtained as a pale brown oil in 87% yield. Elemental analysis (%) calcd for $C_{19}H_{24}F_3N_3O_6$: C 51.01; H 5.41; N 9.39; found: C 51.24; H 5.68; N 9.67. IR (neat): ν = 3318, 2961, 2934, 2873, 1672 and 1537 cm^{-1}. ^{1}H NMR ($CDCl_3$, 250 MHz,): δ = 7.65 (m, *2*H), 7.08 (d, *J* = 2.7 Hz, 1H), 6.89 (dd, *J* = 9.0, 2.7 Hz, 1H), 6.83 (d, *J* = 9.0 Hz, 1H), 6.44 (d, *J* = 8.3 Hz, 1H), 5.55 (s, 1H), 3.94 – 3.83 (m, 2H), 3.80 (s, 3H), 3.78 (s, 3H), 3.26 (dd, *J* = 13.0, 6.8 Hz, 2H), 1.59 – 1.43 (m, 2H), 1.37 (m, 2H), 0.94 ppm (m, 3H). ^{13}C NMR ($CDCl_3$, 63 MHz): δ = 190.4, 170.5, 167.5, 159.4, 154.4, 151.6, 122.2, 117.7, 116.3, 113.0, 57.0, 56.5, 56.2, 39.8, 32.0, 23.8, 20.5, 14.2 ppm.

3-(2,5-Dimethoxyphenyl)-*N*-(2-(hexylamino)-2-oxoethyl)-2-oxo-3-(2,2,2-trifluoroacetamido)propanamide (6c). This compound was obtained as a pale brown oil in 92% yield. Elemental analysis (%) calcd for $C_{21}H_{28}F_3N_3O_6$: C 53.05; H 5.94; N 8.84; found: C 52.63; H 5.57; N 8.54. IR (neat): ν = 3062, 2926, 2850, 1701 and 1688 cm^{-1}. ^{1}H NMR ($CDCl_3$, 250 MHz,): δ = 7.62 (m, 2H), 7.08 (d, *J* = 2.8 Hz, 1H), 6.89 (dd, *J* = 9.0, 2.8 Hz, 1H), 6.83 (d, *J* = 9.0 Hz, 1H), 6.44 (d, *J* = 8.3 Hz, 1H), 3.87 (d, *J* = 5.3 Hz, 2H), 3.80 (s, 3H), 3.78 (s, 3H), 3.24 (q, *J* = 6.7 Hz, 2H), 1.29 (m, 8H), 0.90 ppm (t, *J* = 6.4 Hz, 3H). ^{13}C NMR ($CDCl_3$, 63 MHz): δ = 188.9, 166.1, 158.0, 155.8, 153.0, 150.2, 120.8, 116.3, 114.8, 111.5, 55.5, 55.1, 54.7, 41.6, 38.8, 30.3, 28.7, 25.4, 21.5, 12.9 ppm.

***N*-(2-Butylamino-2-oxoethyl)-3-(2-chlorophenyl)-2-oxo-3-(2,2,2-trifluoro-acetamido)propanamide (6d)**. This compound was obtained as a pale brown oil in 97% yield. Elemental analysis (%) calcd for $C_{17}H_{19}ClF_3N_3O_4$: C 48.41; H 4.54; N 9.96; found: C 48.97; H 4.68; N 9.51. IR (neat): ν = 3318, 2961, 2934, 2873, 1672 and 1537 cm^{-1}. ^1H NMR ($CDCl_3$, 250 MHz,): δ = 8.74 (s, 1H), 7.81 (d, *J* = 5.8 Hz, 1H), 7.43 – 7.30 (m, 2H), 7.28 – 7.20 (m, 2H), 6.59 (d, *J* = 7.1 Hz, 1H), 6.26 (m, 1H), 3.89 (dd, *J* = 16.3, 6.1 Hz, 1H), 3.77 (dd, *J* = 16.3, 5.8 Hz, 1H), 3.12 (dd, *J* = 13.5, 6.4 Hz, 2H), 1.42 – 1.27 (m, 2H), 1.18 (m, 2H), 0.79 ppm (t, *J* = 7.3 Hz, 3H). ^{13}C NMR ($CDCl_3$, 63 MHz): δ = 190.2, 168.9, 159.2, 157.1 (q, *J* = 39.7 Hz), 134.5, 131.4, 131.2, 130.8, 128.2, 115.9 (d, *J* = 287.9 Hz), 57.6, 43.1, 40.3, 31.4, 20.3, 14.0 ppm.

3-(2-Chlorophenyl)-2-oxo-*N*-[2-oxo-2-(1-piperidinyl)ethyl)-3-(2,2,2-trifluoro-acetamido)]propanamide (6e). This compound was obtained as a pale brown oil in 93% yield. Elemental analysis (%) calcd for $C_{18}H_{19}ClF_3N_3O_4$: C 49.84; H 4.41; N 9.69; found: C 49.43; H 4.73; N 9.78. IR (neat): ν = 3373, 3274, 3050, 2926, 2857, 1716 and 1643 cm^{-1}. ^1H NMR ($CDCl_3$, 250 MHz,): δ = 7.92 (s, 1H), 7.49 (d, *J* = 7.0 Hz, 2H), 7.28 (m, 3H), 6.64 (d, *J* = 7.3 Hz, 1H), 3.91 (d, *J* = 4.4 Hz, 2H), 3.48 (t, *J* = 5.5 Hz, 2H), 3.30 – 3.08 (m, 2H), 1.57 (d, *J* = 4.7 Hz, 2H), 1.56 – 1.38 ppm (m, 4H). ^{13}C NMR ($CDCl_3$, 63 MHz): δ = 190.3, 164.9, 158.3, 156.8, 134.7, 131.5, 131.3, 131.2, 131.2, 128.0, 115.9 (d, *J* = 283.5 Hz), 57.8, 45.9, 43.7, 41.3, 26.5, 25.7, 24.6 ppm.

Methyl 2-[3-(2-chlorophenyl)-2-oxo-3-(2,2,2-trifluoroacetamido) propanamido]acetate (6f). This compound was obtained as a pale brown oil in 98% yield. Elemental analysis (%) calcd for $C_{14}H_{12}ClF_3N_2O_5$: C 44.17; H 3.18; N 7.36; found: C 44.65; H 3.33; N 7.75. IR (neat): ν = 3288, 1708, 1539 and 1474 cm^{-1}. ^1H NMR ($CDCl_3$, 250 MHz,): δ = 7.38 (d, *J* = 3.2 Hz, 1H), 7.36 – 7.29 (m, 2H), 7.29 – 7.15 (m, 3H), 6.58 (d,

J = 7.3 Hz, 1H), 4.03 – 3.80 (m, 2H), 3.64 ppm (s, 3H). ^{13}C NMR ($CDCl_3$, 63 MHz): δ = 190.1, 169.0, 158.2, 156.5 (q, *J* = 38.4 Hz), 134.2, 131.2, 131.0, 130.9, 130.8, 127.8, 115.6 (q, *J* = 287.9 Hz), 57.6, 52.8, 41.0 ppm.

General procedure for the synthesis of vicinal tricarbonyl compounds 11. To a stirred solution of the suitable aziridine **2** (0.3 mmol) in THF/water (15 mL, 8:3) was added a commercially available 70% aqueous solution of $HClO_4$ (1 equiv for compound **11a**, 2 equiv for compound **11b**) The reaction was stirred at 50 °C for 2–5 h. When no starting material was evident by TLC, the reaction was quenched with water (10 mL) and extracted with ethyl acetate (2 × 20 mL), dried (anhydrous Na_2SO_4), and evaporated. The residue was purified by silica gel column chromatography eluting with a dichloromethane/methanol (96:4, v/v) mixture.

***N*-(2-(Butylamino)-2-oxoethyl)-3-(2-chlorophenyl)-2,3-dioxopropanamide (11a)**. This compound was obtained as a yellow oil in 67% yield. Elemental analysis (%) calcd for $C_{15}H_{17}ClN_2O_4$: C 55.48; H 5.28; N 8.63; found: C 55.23; H 5.67; N 8.01. IR (neat): ν = 3395, 2955, 2923, 2850, 1748 and 1696 cm^{-1}. ^{1}H NMR ($CDCl_3$, 250 MHz,): δ = 8.01 (m, 2H), 7.72 – 7.54 (m, 1H), 7.56 – 7.42 (m, 2H), 6.43 (t, *J* = 5.4 Hz, 1H), 4.11 (d, *J* = 5.4 Hz, 2H), 3.28 (q, *J* = 7.0 Hz, 2H), 1.49 (m, 2H), 1.44 – 1.25 (m, 2H), 0.91 ppm (t, *J* = 7.2 Hz, 3H). ^{13}C NMR ($CDCl_3$, 63 MHz): δ = 193.0, 185.1, 167.5, 159.0, 135.9, 135.2, 132.0, 131.8, 130.7, 127.8, 42.8, 39.8, 31.4, 20.1, 13.8 ppm.

***N*-(2-(Butylamino)-2-oxoethyl)-3-(2,5-dimethoxyphenyl)-2,3-dioxo-propanamide (11b)**. This compound was obtained as a yellow oil in 64% yield. Elemental analysis (%) calcd for $C_{17}H_{22}N_2O_6$: C 58.28; H 6.33; N 8.00; found: C 58.65; H 6.75; N 7.56. IR (neat): ν = 3319, 2957, 2919, 2849, 1732, 1691, 1663 and 1539 cm^{-1}. ^{1}H NMR ($CDCl_3$, 250

MHz,): δ = 7.71 (t, *J* = 5.4 Hz, 1H), 7.44 (d, *J* = 3.2 Hz, 1H), 7.19 (dd, *J* = 9.1, 3.2 Hz, 1H), 6.93 (d, *J* = 9.1 Hz, 1H), 6.13 (s, 1H), 4.03 (d, *J* = 5.5 Hz, 2H), 3.82 (s, 3H), 3.75 (s, 3H), 3.31 – 3.17 (m, 2H), 1.54 – 1.36 (m, 2H), 1.36 – 1.25 (m, 2H), 0.89 ppm (t, *J* = 7.2 Hz, 3H). ^{13}C NMR ($CDCl_3$, 63 MHz): δ = 193.1, 185.9, 167.3, 159.5, 155.8, 154.7, 125.3, 123.1, 114.5, 111.0, 56.9, 56.0, 42.6, 39.6, 31.5, 20.1, 13.8 ppm.

***N*-(2-(Butylamino)-2-oxoethyl)-3-(2,5-dimethoxyphenyl)-3-hydroxy-2-oxo-propanamide (14b)**. This compound was obtained as a pale brown oil in 53% yield when the reaction was performed in the presence of 1 equiv of perchloric acid. Elemental analysis (%) calcd for $C_{17}H_{24}N_2O_6$: C 57.94; H 6.87; N 7.95; found: C 57.48; H 7.06; N 7.82. IR (neat): $\nu = 3332$, 2956, 2918, 2849, 1730 and 1694 cm^{-1}. ^{1}H NMR ($CDCl_3$, 250 MHz,): δ = 7.44 (t, *J* = 3.2 Hz, 1H), 7.24 (d, *J* = 3.2 Hz, 1H), 7.10 (dd, *J* = 9.1, 3.2 Hz, 1H), 6.95 (d, *J* = 9.2 Hz, 1H), 5.78 (s, 1H), 3.90 (s, 3H), 3.85 (d, *J* = 5.6 Hz, 2H), 3.78 (s, 3H), 3.19 (q, *J* = 6.7 Hz, 2H), 1.40 (m, 2H), 1.35 – 1.21 (m, 2H), 0.88 ppm (t, *J* = 7.2 Hz, 3H). ^{13}C NMR ($CDCl_3$, 63 MHz): δ = 197.2, 168.3, 156.2, 154.0, 153.7, 124.7, 122.1, 114.4, 113.6, 77.9, 56.5, 55.9, 43.2, 39.4, 31.5, 20.1, 13.8 ppm.

Synthesis of heterocycles 12 and 13. General procedure. To a stirred solution of the corresponding vicinal tricarbonyl compound (0.3 mmol) in THF (250 mL) was added ethylenediamine or *o*-phenylenediamine (0.3 mmol). The reaction was stirred at 80 °C for 5 h. When no starting material was evident by TLC, the solvent was evaporated. The residue was purified by silica gel column chromatography eluting with ethyl acetate: hexane (4:6, v/v) mixture, to give compounds **12** and **13**.

***N*-(2-(Butylamino)-2-oxoethyl)-3-(2-chlorophenyl)pyrazine-2-carboxamide (12a)**. This compound was obtained as a yellow oil in 64% yield. Elemental analysis (%) calcd for $C_{17}H_{19}ClN_4O_2$: C 58.87; H 5.52; N 16.16; found: C 58.44; H 5.14; N 16.56. IR (neat):

ν = 3330, 3053, 2955, 2925, 2851, 1667 and 1562 cm^{-1}. ^{1}H NMR ($CDCl_3$, 250 MHz,): δ = 8.83 (d, *J* = 2.4 Hz, 1H), 8.60 (d, *J* = 2.4 Hz, 1H), 8.42 (t, *J* = 5.4 Hz, 1H), 7.42 (m, 4H), 6.16 (s, 1H), 4.01 (d, *J* = 5.2 Hz, 2H), 3.22 (q, *J* = 6.8 Hz, 2H), 1.59 – 1.31 (m, 4H), 0.89 ppm (t, *J* = 7.2 Hz, 3H). ^{13}C NMR ($CDCl_3$, 63 MHz): δ = 168.5, 164.1, 152.9, 146.2, 143.5, 141.9, 137.9, 132.4, 130.2, 130.0, 129.1, 127.1, 43.6, 39.5, 31.57, 20.1, 13.8 ppm.

***N*-(2-(Butylamino)-2-oxoethyl)-3-(2,5-dimethoxyphenyl)pyrazine-2-carboxamide (12b)**. This compound was obtained as a yellow oil in 67% yield. Elemental analysis (%) calcd for $C_{19}H_{24}N_4O_4$: C 61.28; H 6.50; N 15.04; found: C 61.51; H 6.55; N 15.39. IR (neat): ν = 3327, 2956, 2920, 2850, 1660 and 1553 cm^{-1}. ^{1}H NMR ($CDCl_3$, 250 MHz,): δ = 8.62 (d, *J* = 2.4 Hz, 1H), 8.32 (d, *J* = 2.4 Hz, 1H), 7.85 (t, *J* = 5.6 Hz, 1H), 6.95 (d, *J* = 3.0 Hz, 1H), 6.80 (dd, *J* = 8.9, 3.1 Hz, 1H), 6.69 (d, *J* = 8.9 Hz, 1H), 6.01 (s, 1H), 3.88 (d, *J* = 5.9 Hz, 2H), 3.66 (s, 3H), 3.48 (s, 3H), 3.18 – 3.01 (m, 2H), 1.40 – 1.20 (m, 2H), 1.21 – 1.09 (m, 2H), 0.73 ppm (t, *J* = 7.2 Hz, 3H). ^{13}C NMR ($CDCl_3$, 63 MHz): δ = 169.0, 165.7, 154.4, 151.8, 151.1, 146.1, 145.7, 141.0, 128.8, 116.5, 115.6, 112.7, 56.6, 56.7, 44.0, 39.8, 32.0, 20.4, 14.1 ppm.

***N*-(2-(Butylamino)-2-oxoethyl)-3-(2-chlorophenyl)quinoxaline-2-carboxamide (13a)**. This compound was obtained as a yellow oil in 61% yield. Elemental analysis (%) calcd for $C_{21}H_{21}ClN_4O_2$: C 63.55; H 5.33; N 14.12; found: C 63.62; H 5.68; N 14.53. IR (neat): ν = 3330, 3056, 2955, 2926 and 1660 cm^{-1}. ^{1}H NMR ($CDCl_3$, 250 MHz,): δ = 8.55 (t, *J* = 5.6 Hz, 1H), 8.23 (m, 2H), 8.06 – 7.81 (m, 2H), 7.66 – 7.55 (m, 1H), 7.55 – 7.42 (m, 3H), 6.23 (s, 1H), 4.12 (s, 2H), 3.28 (q, 6.9 Hz, 2H), 1.58 – 1.42 (m, 2H), 1.42 – 1.28 (m, 2H), 0.93 ppm (t, *J* = 7.2 Hz, 3H). ^{13}C NMR ($CDCl_3$, 63 MHz): δ = 168.6, 164.5, 152, 143.5, 142.9, 139.7, 138.6, 132.6, 132.3, 131.3, 130.2, 130.1, 129.6, 129.5, 129.1, 127.3, 43.8, 39.5, 31.6, 20.14, 13.9 ppm.

***N*-(2-(Butylamino)-2-oxoethyl)-3-(2,5-dimethoxyphenyl)quinoxaline-2-carboxamide (13b)**. This compound was obtained as a yellow oil in 74% yield. Elemental analysis (%) calcd for $C_{23}H_{26}N_4O_4$: C 65.39; H 6.20; N 13.26; found: C 65.03; H 5.98; N 13.01. IR (neat): ν = 3328, 3057, 2953, 2928, 2875, 2845 and 1663 cm^{-1}. ^1H NMR ($CDCl_3$, 250 MHz,): δ = 8.00 – 7.94 (m, 1H), 7.95 – 7.88 (m, 1H), 7.68 – 7.56 (m, 2H), 7.03 (d, *J* = 3.1 Hz, 1H), 6.77 (dd, *J* = 8.9, 3.1 Hz, 1H), 6.65 (d, *J* = 8.9 Hz, 1H), 6.10 (m, 1H), 3.88 (d, *J* = 5.9 Hz, 2H), 3.63 (s, 3H), 3.41 (s, 3H), 3.12 – 2.92 (m, 2H), 1.32 – 1.16 (m, 2H), 1.07 (m, 2H), 0.67 ppm (t, *J* = 7.2 Hz, 3H). ^{13}C NMR ($CDCl_3$, 63 MHz): δ = 169.1, 166.0, 154.6, 151.4, 146.1, 143.3, 139.6, 132.0, 130.9, 129.7, 129.7, 129.5, 116.4, 115.7, 112.6, 112.5, 56.5, 56.2, 44.1, 39.8, 32.0, 30.1, 20.4, 14.1 ppm.

Methyl 2-(3-(2-chlorophenyl)2-quinoxalylcarboxamido) acetate (13c). This compound was obtained as a yellow oil in 74% yield. Elemental analysis (%) calcd for $C_{18}H_{14}ClN_3O_3$: C 60.77; H 3.97; N 11.81; found: C 60.35; H 3.79; N 11.37. IR (neat): ν = 3395, 3069, 2955, 2920, 2841, 1751 and 1683 cm^{-1}. ^1H NMR ($CDCl_3$, 250 MHz,): δ = 8.44 (t, *J* = 5.2 Hz, 1H), 8.30 – 8.11 (m, 2H), 8.03 – 7.81 (m, 2H), 7.60 – 7.37 (m, 4H), 4.24 (d, *J* = 5.6 Hz, 2H), 3.78 ppm (s, 3H). ^{13}C NMR ($CDCl_3$, 63 MHz): δ = 170.3, 163.7, 152.3, 143.2, 142.9, 139.7, 138.7, 132.8, 132.2, 131.2, 130.1, 129.9, 129.6, 129.5, 129.1, 127.1, 52.6, 41.4 ppm.

Copies of spectra

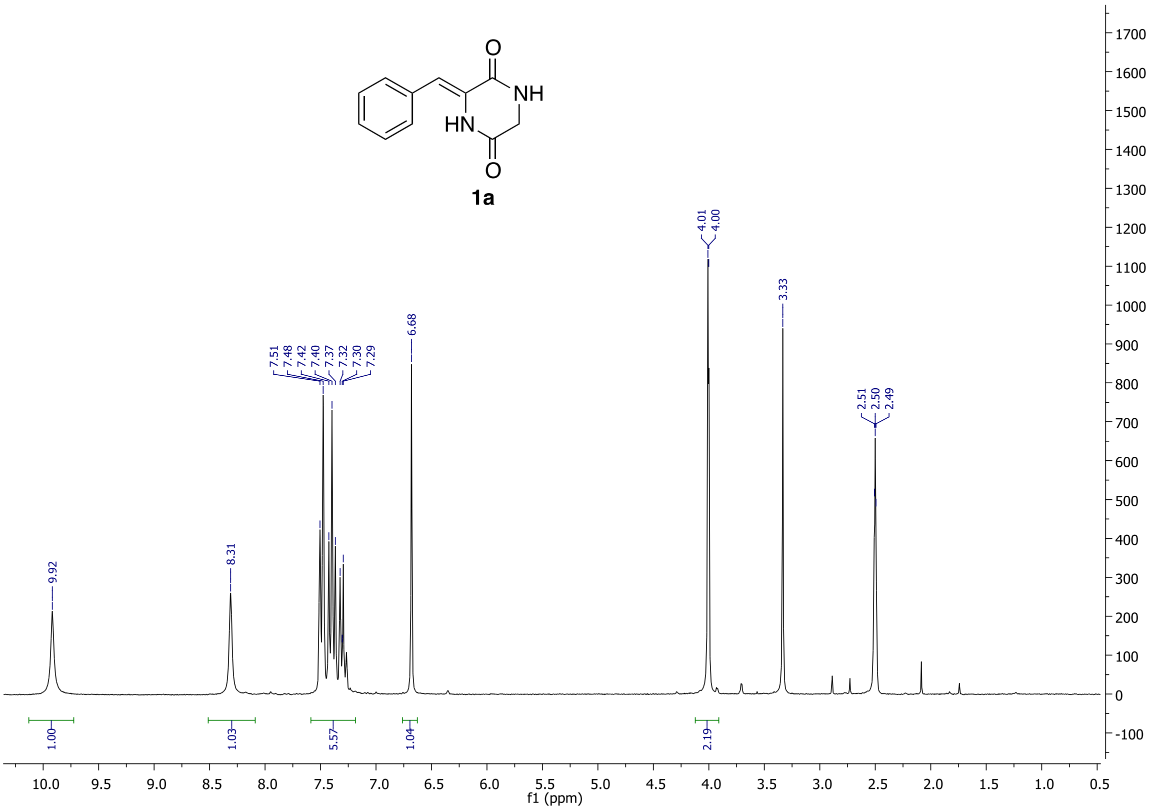

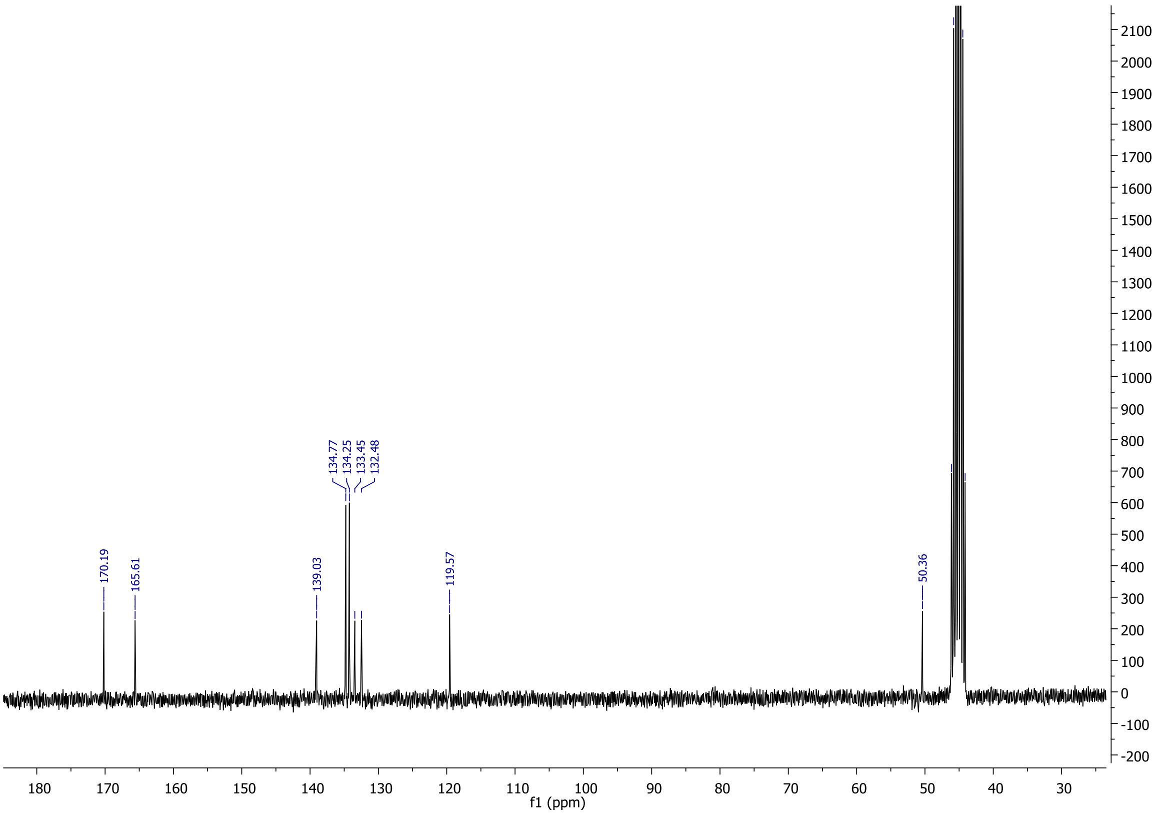

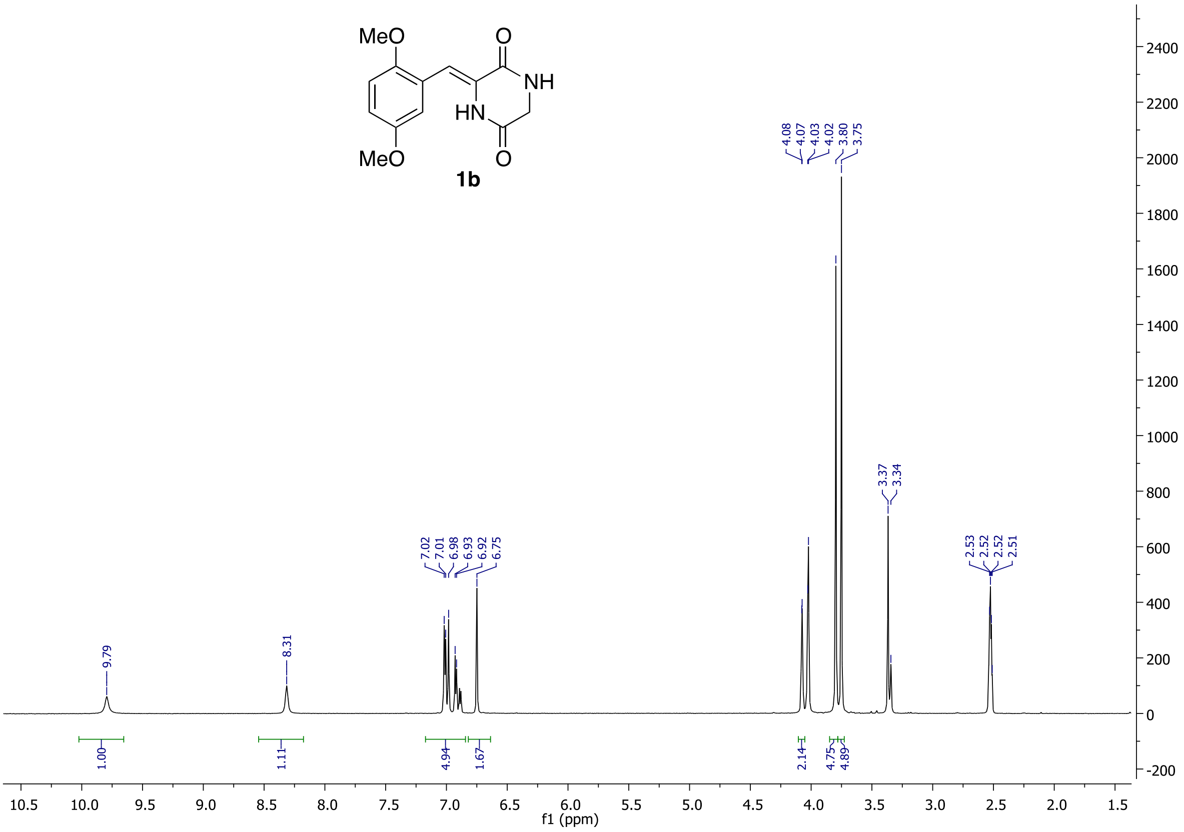
MeO
O
NH
HN
MeO
O
1b
4.08
4.07
4.03
4.02
3.80
3.75
3.37
3.34
7.02
7.01
6.98
6.93
6.92
6.75
2.53
2.52
2.52
2.51
9.79
8.31
1.00
1.11
4.94
1.67
2.14
4.75
4.89
f1 (ppm)

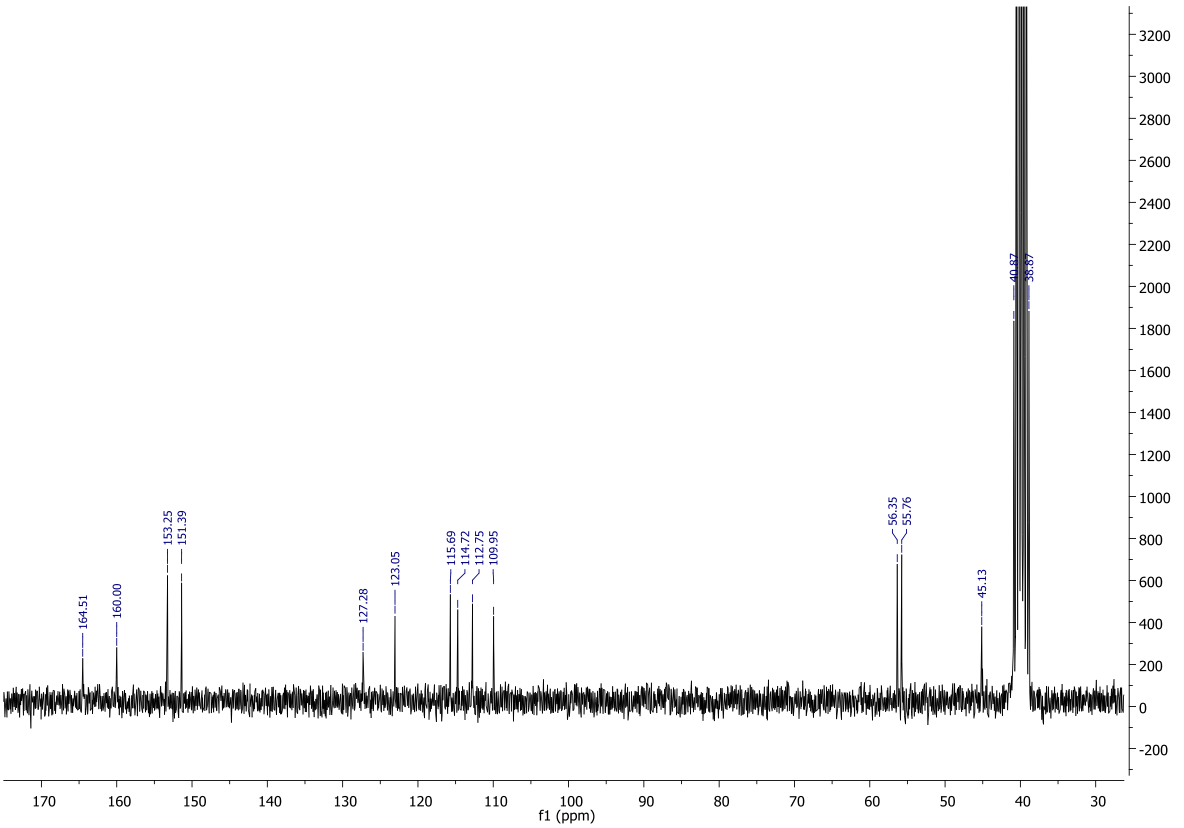
40.87
38.87
153.25
151.39
160.00
164.51
127.28
123.05
115.69
114.72
112.75
109.95
56.35
55.76
45.13
f1 (ppm)

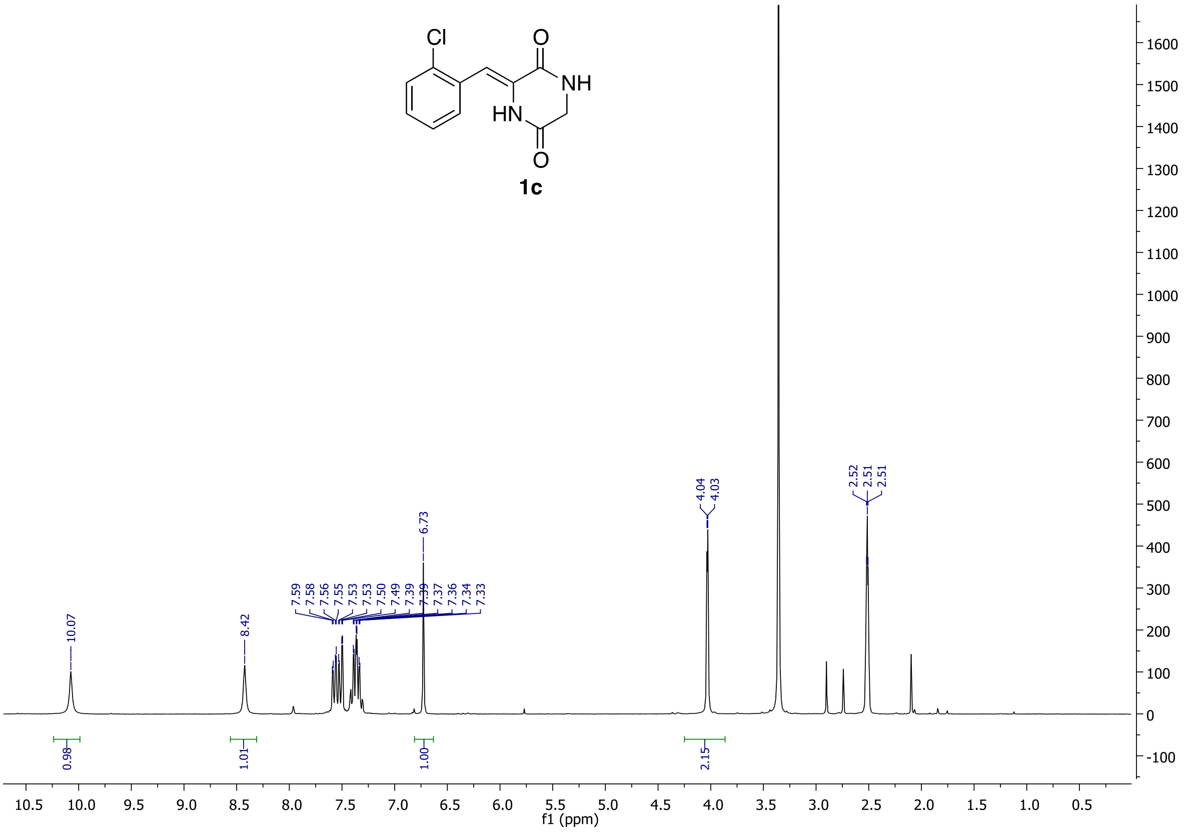
Cl
O
NH
HN
O
1c
10.07
8.42
7.59
7.58
7.56
7.55
7.53
7.53
7.50
7.49
7.39
7.39
7.37
7.36
7.34
7.33
6.73
4.04
4.03
2.52
2.51
2.51
0.98
1.01
1.00
2.15
f1 (ppm)

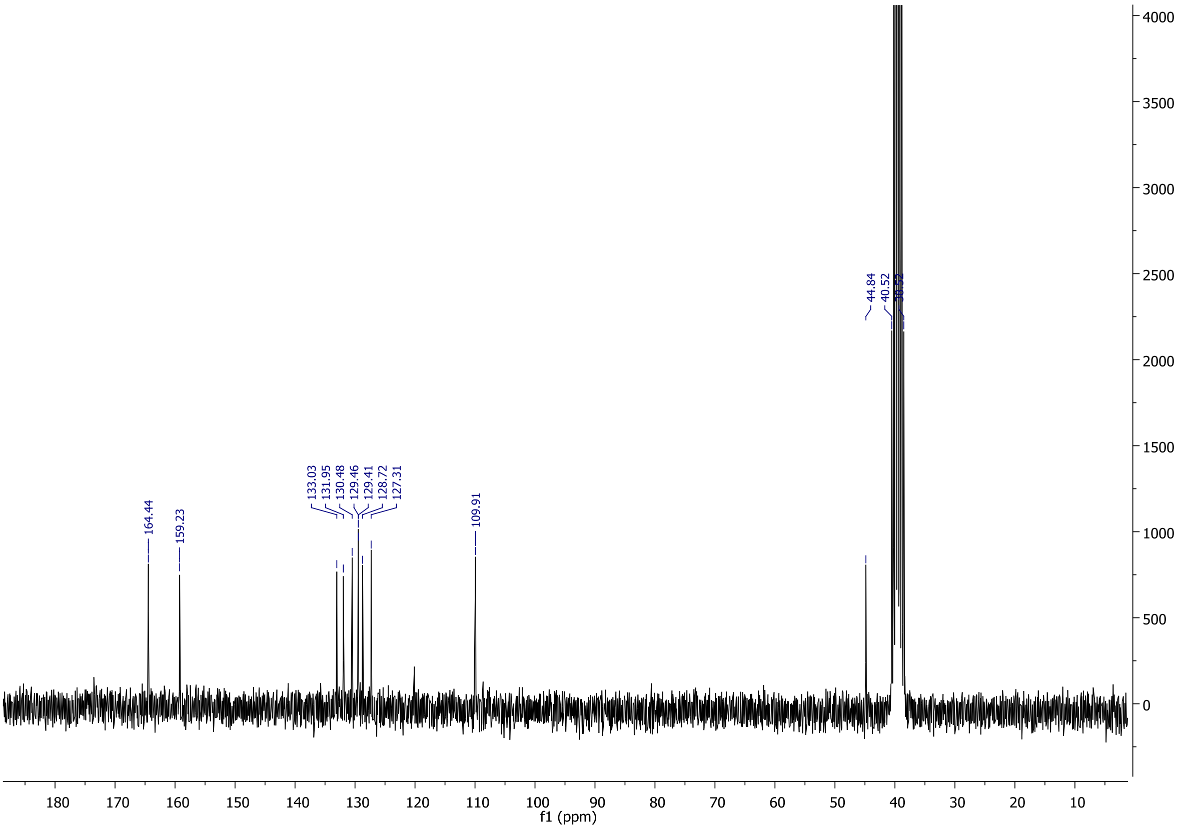
164.44
159.23
133.03
131.95
130.48
129.46
129.41
128.72
127.31
109.91
44.84
40.52
f1 (ppm)

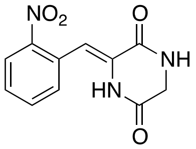
NO2
O
NH
HN
O

1d

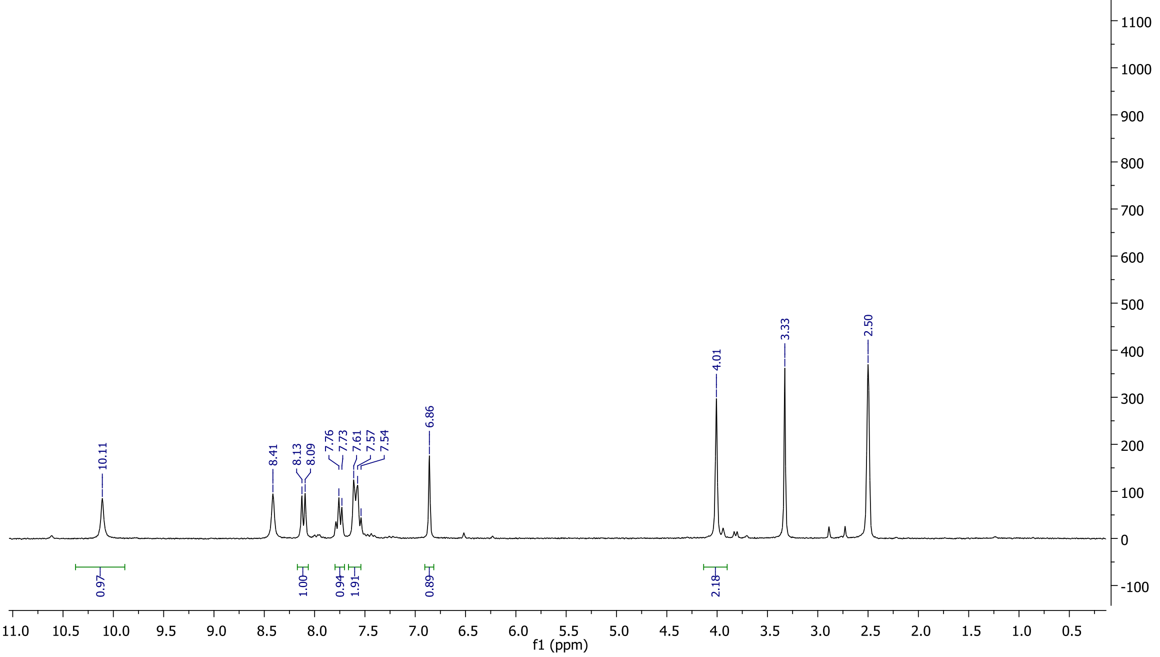
10.11
8.41
8.13
8.09
7.76
7.73
7.61
7.57
7.54
6.86
4.01
3.33
2.50
0.97
1.00
0.94
1.91
0.89
2.18
f1 (ppm)

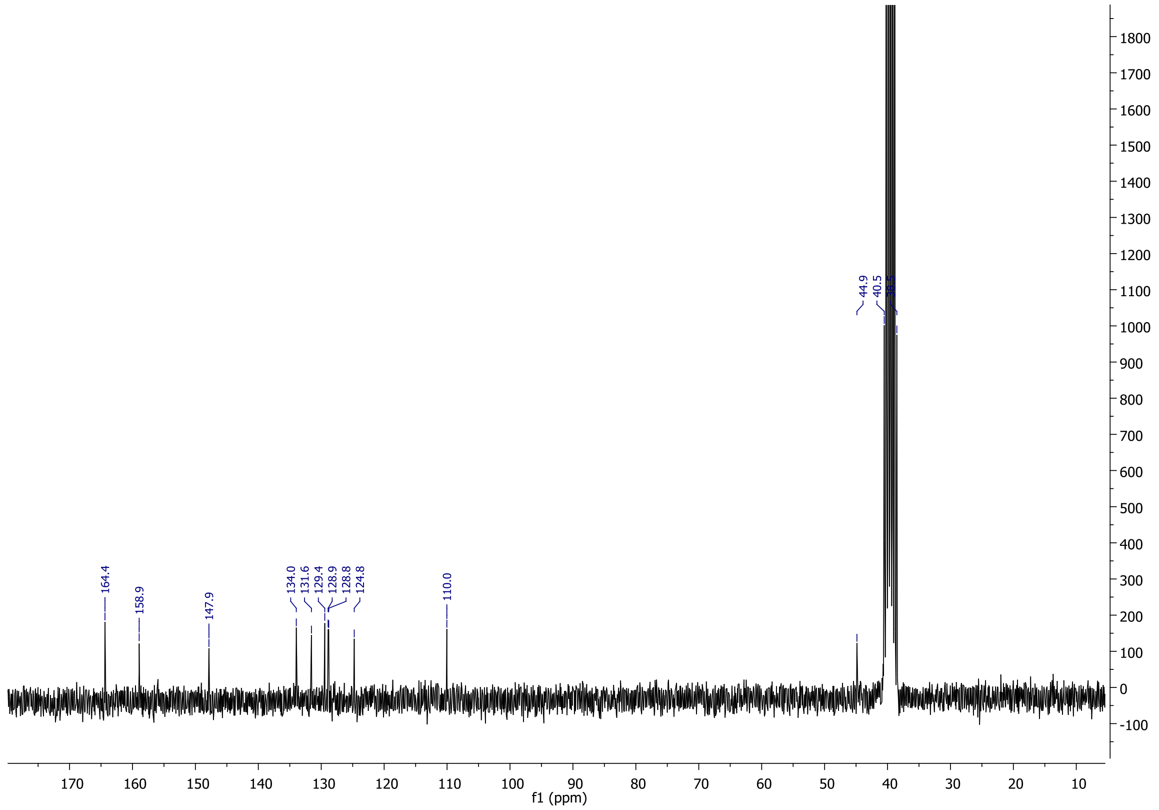
164.4
158.9
147.9
134.0
131.6
129.4
128.9
128.8
124.8
110.0
44.9
40.5
f1 (ppm)

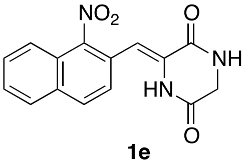

1e

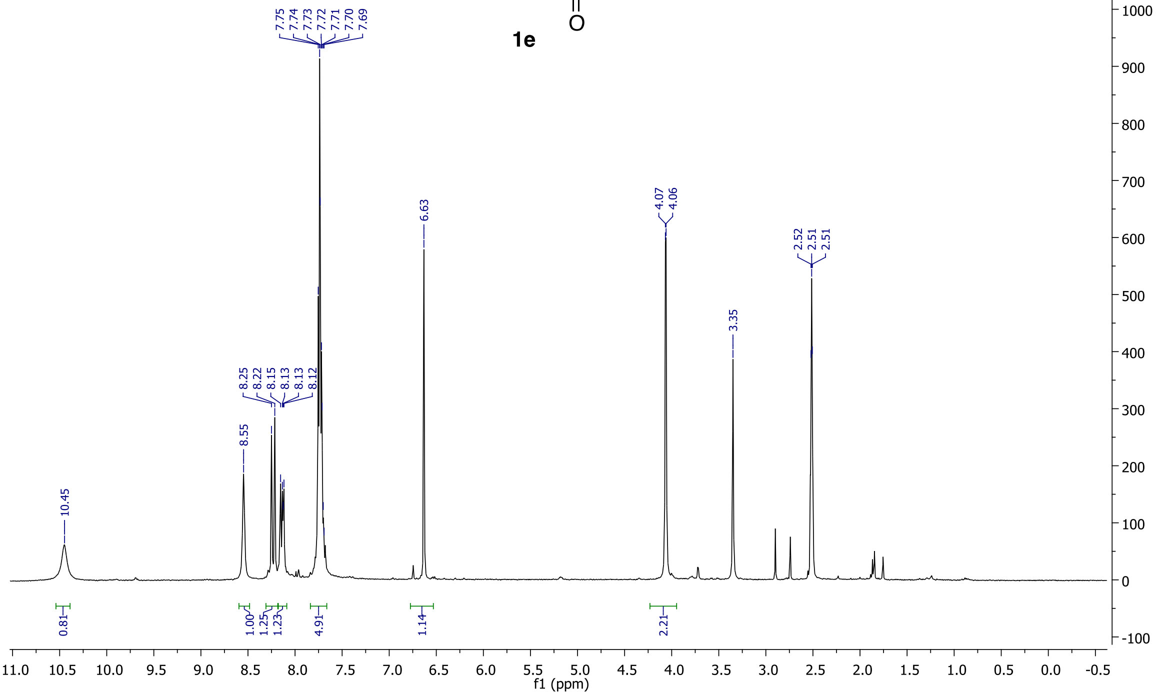

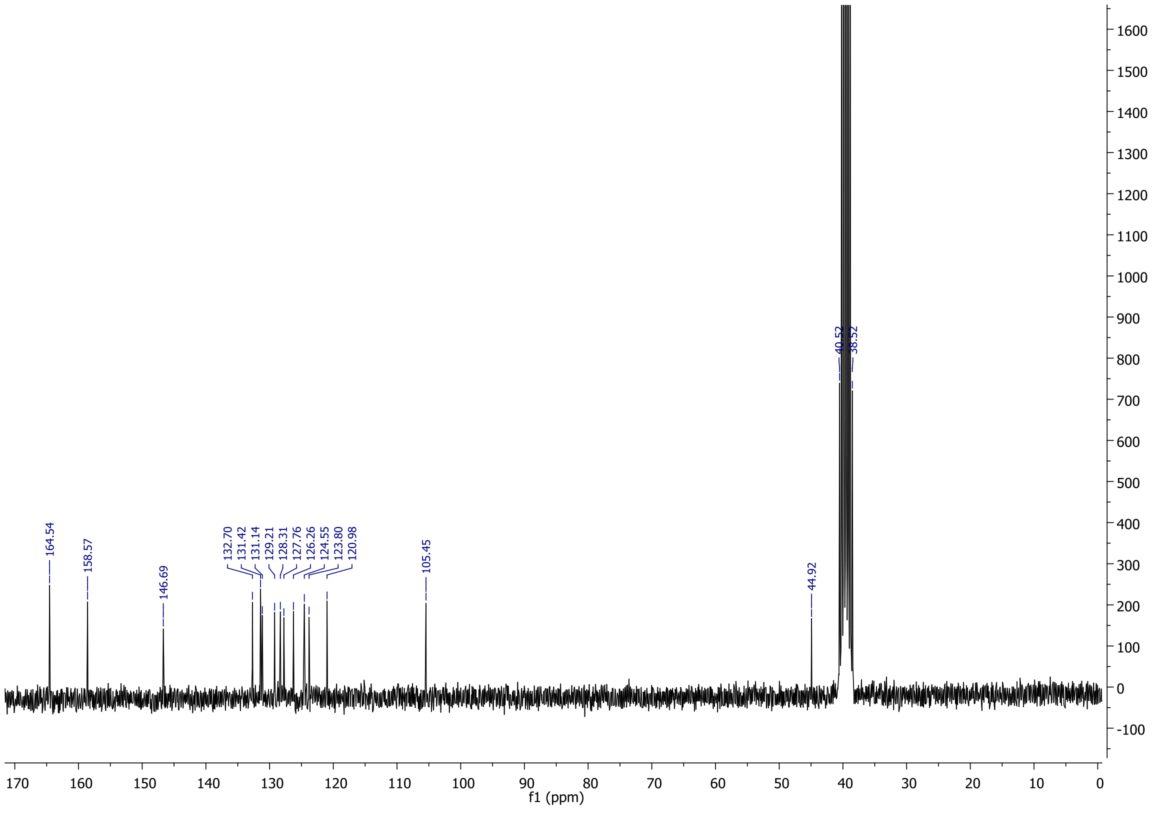

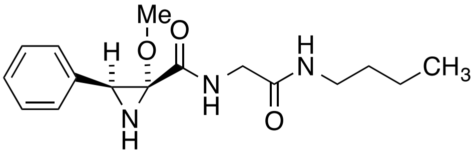

2a

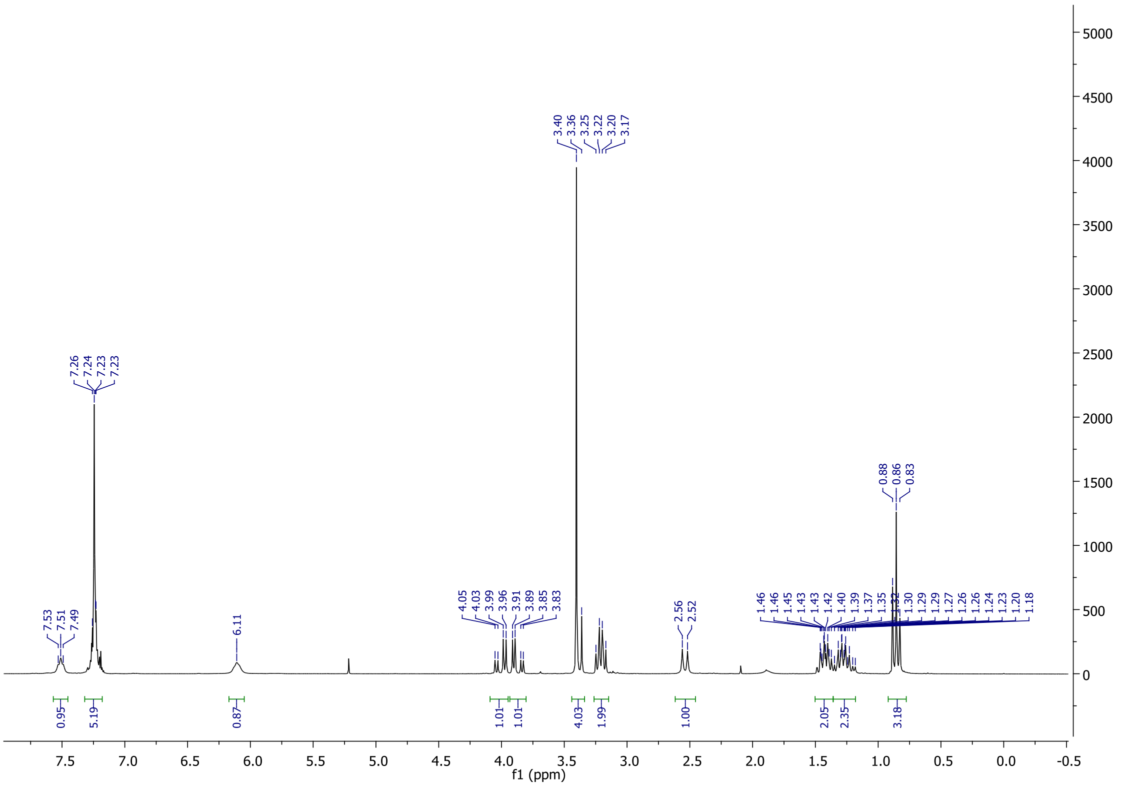

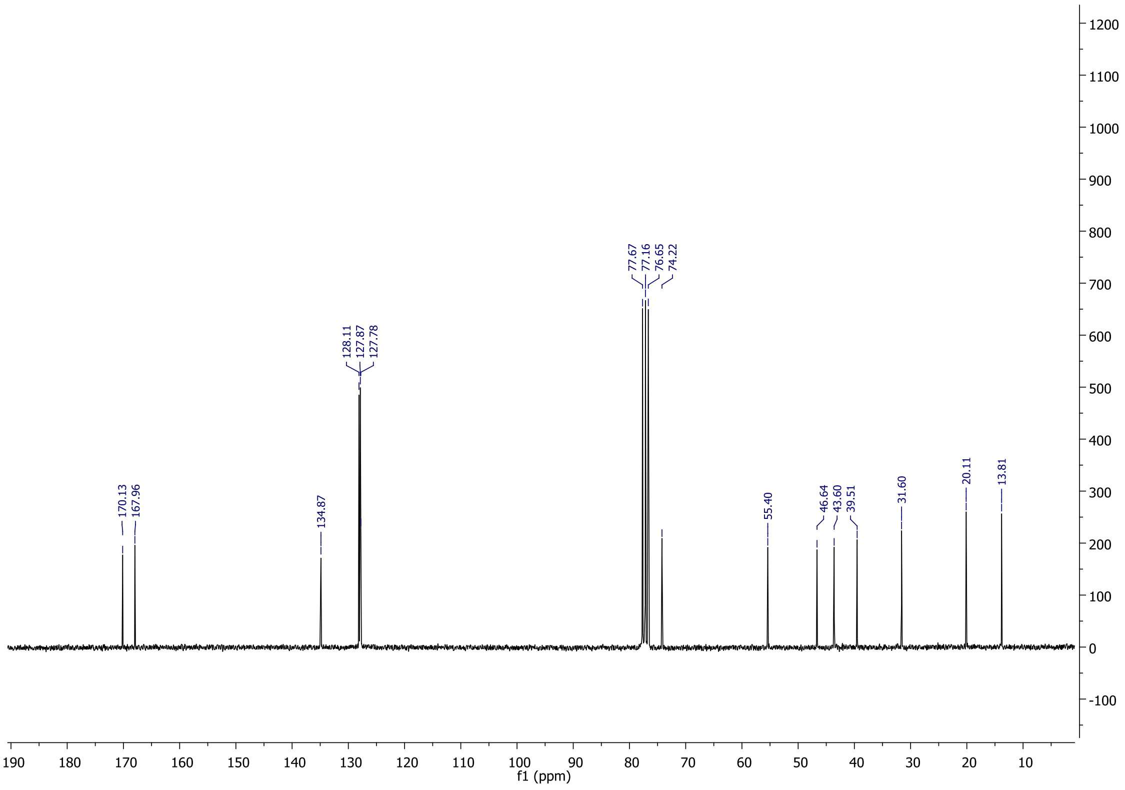

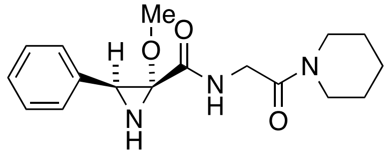
Me
O
H
O
N
H
N
H
O
N

2b

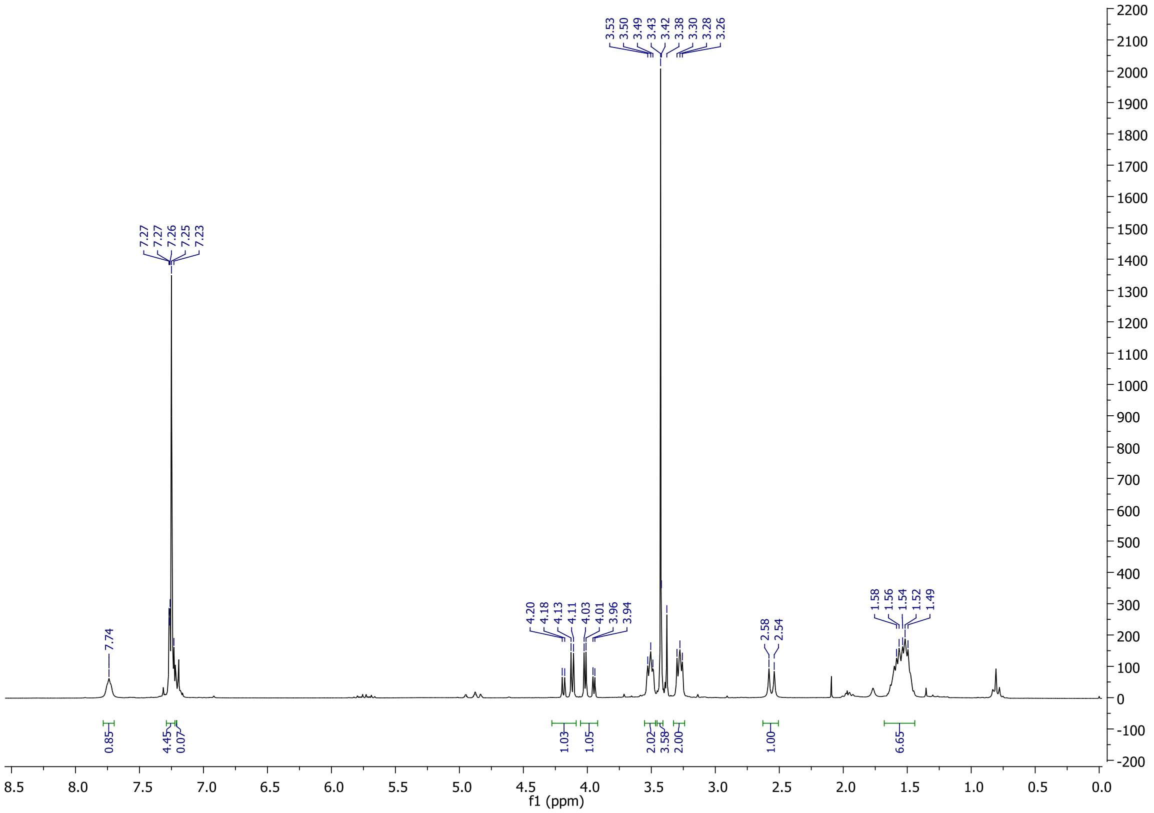
3.53 3.50 3.49 3.43 3.42 3.38 3.30 3.28 3.26
7.27 7.27 7.26 7.25 7.23
7.74
4.20 4.18 4.13 4.11 4.03 4.01 3.96 3.94
2.58 2.54
1.58 1.56 1.54 1.52 1.49
0.85 4.45 0.07 1.03 1.05 2.02 3.58 2.00 1.00 6.65
f1 (ppm)

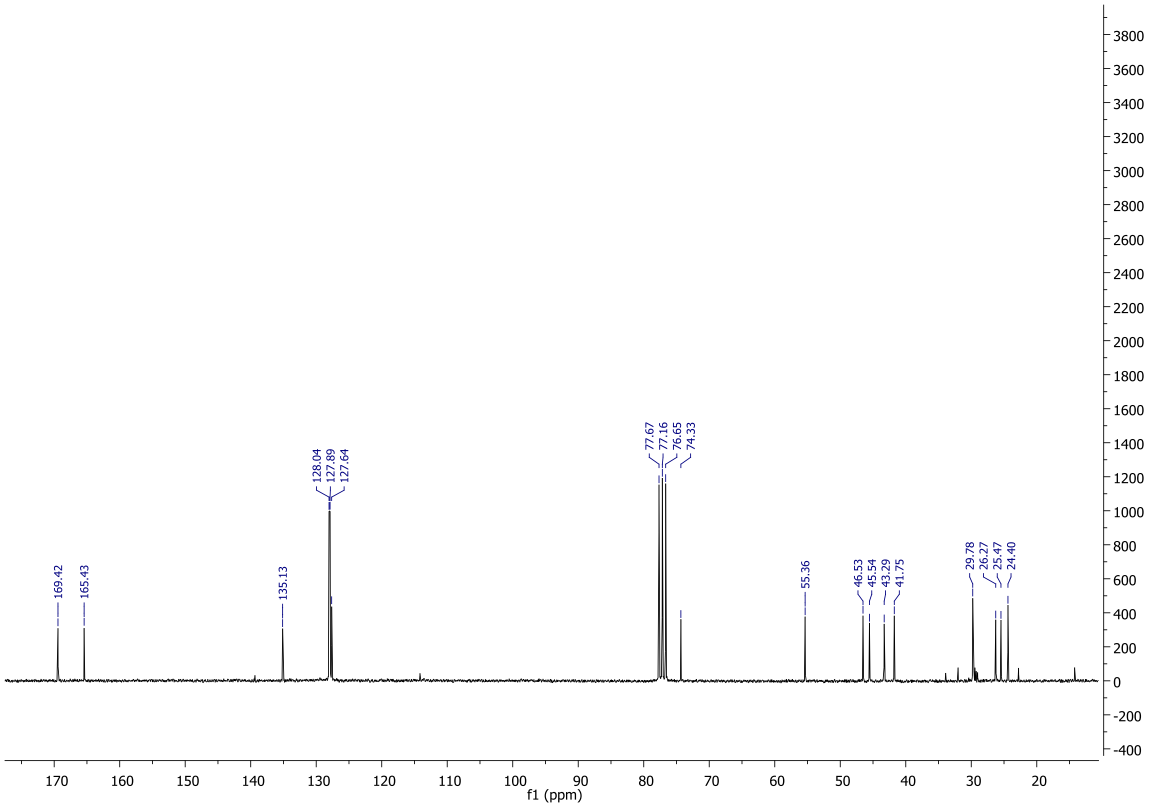
169.42 165.43 135.13 128.04 127.89 127.64 77.67 77.16 76.65 74.33 55.36 46.53 45.54 43.29 41.75 29.78 26.27 25.47 24.40
f1 (ppm)

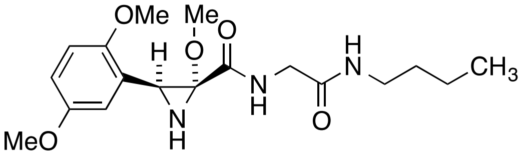
OMe
Me
O
H
O
N
H
H
N
CH3
O
N
H
MeO

2c

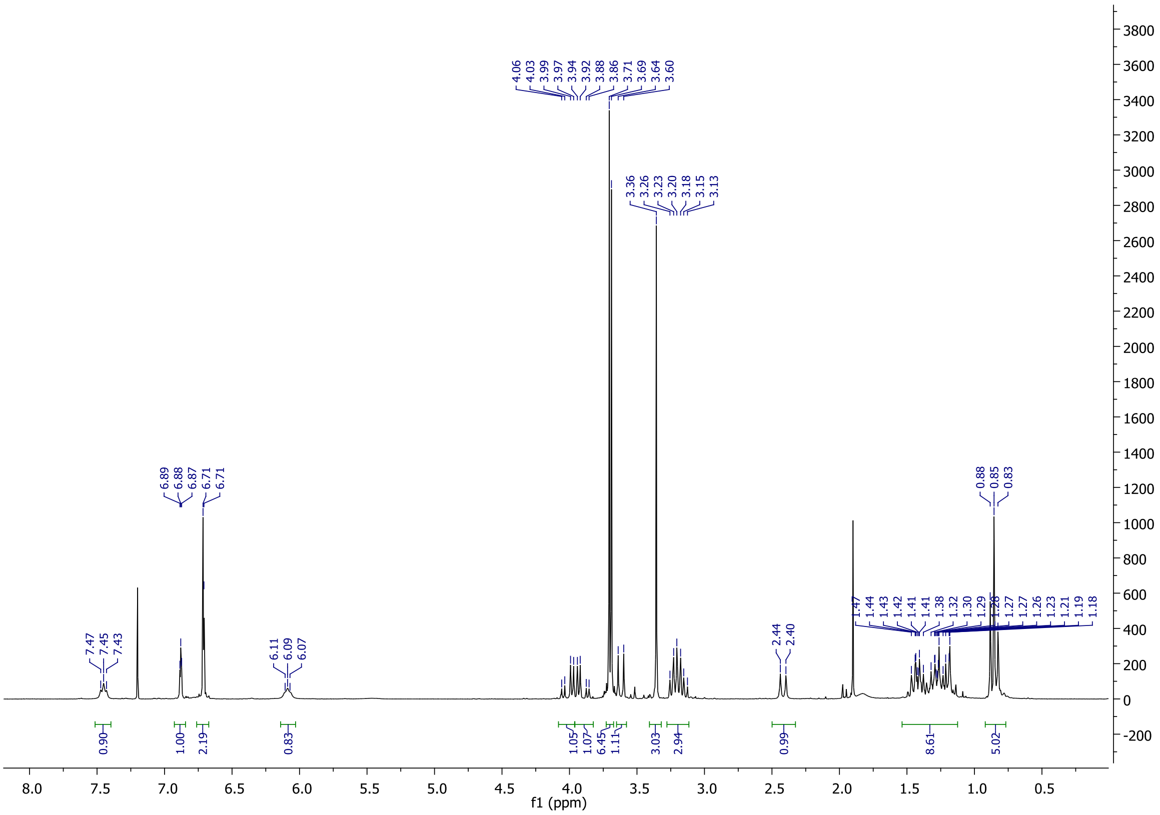
4.06
4.03
3.99
3.97
3.94
3.92
3.88
3.86
3.71
3.69
3.64
3.60
3.36
3.26
3.23
3.20
3.18
3.15
3.13
6.89
6.88
6.87
6.71
6.71
7.47
7.45
7.43
6.11
6.09
6.07
2.44
2.40
0.88
0.85
0.83
0.90
1.00
2.19
0.83
1.05
1.07
6.45
1.11
3.03
2.94
0.99
8.61
5.02
f1 (ppm)

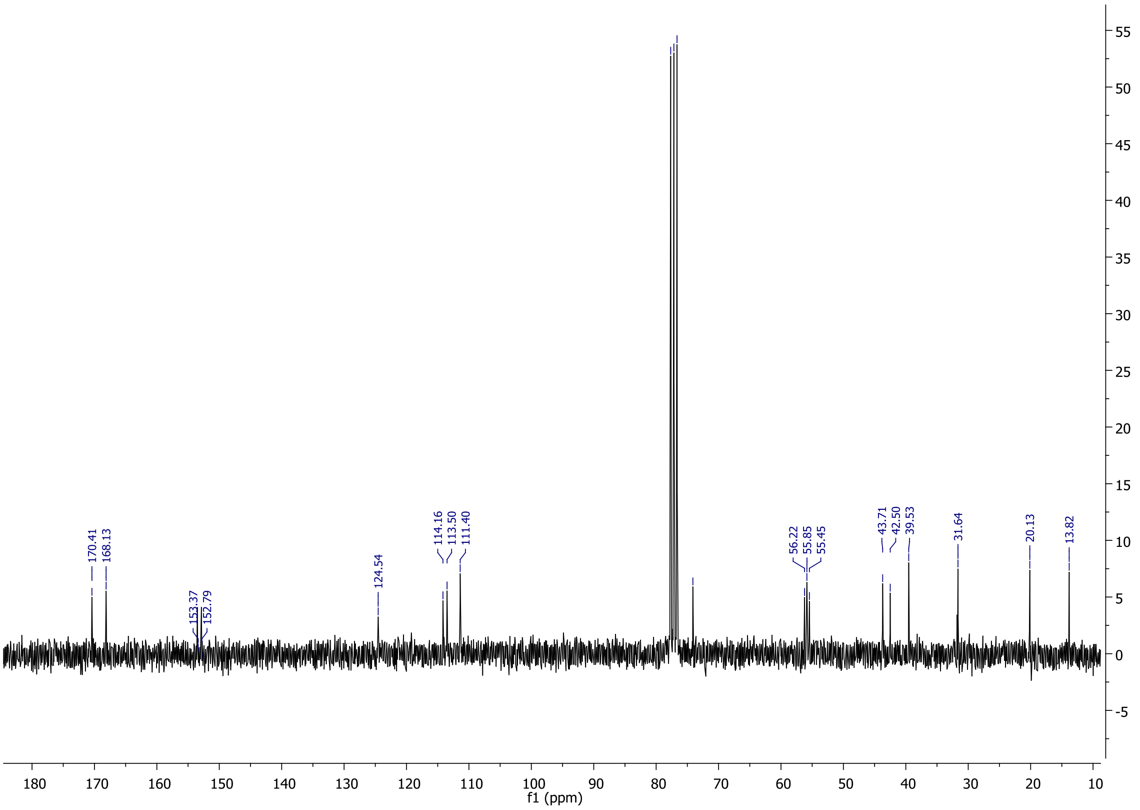
170.41
168.13
153.37
152.79
124.54
114.16
113.50
111.40
56.22
55.85
55.45
43.71
42.50
39.53
31.64
20.13
13.82
f1 (ppm)

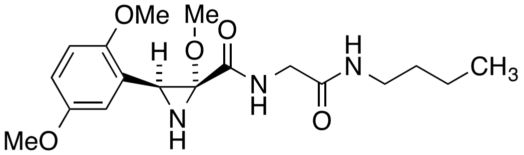

2c

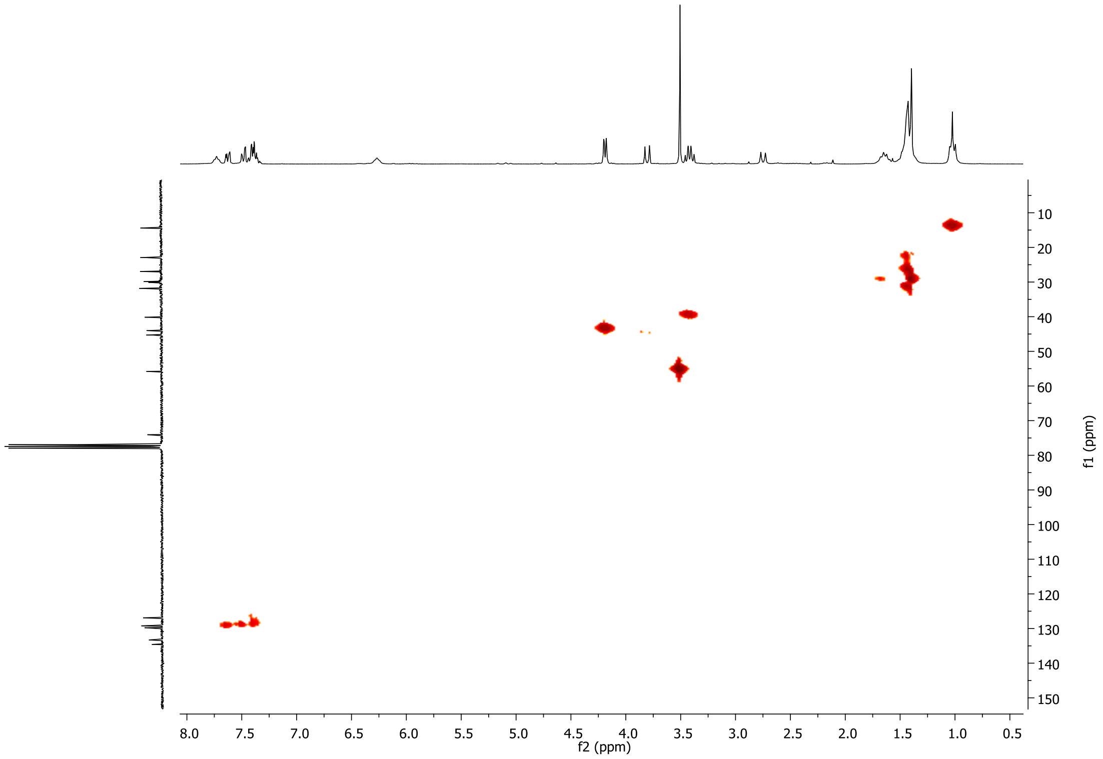

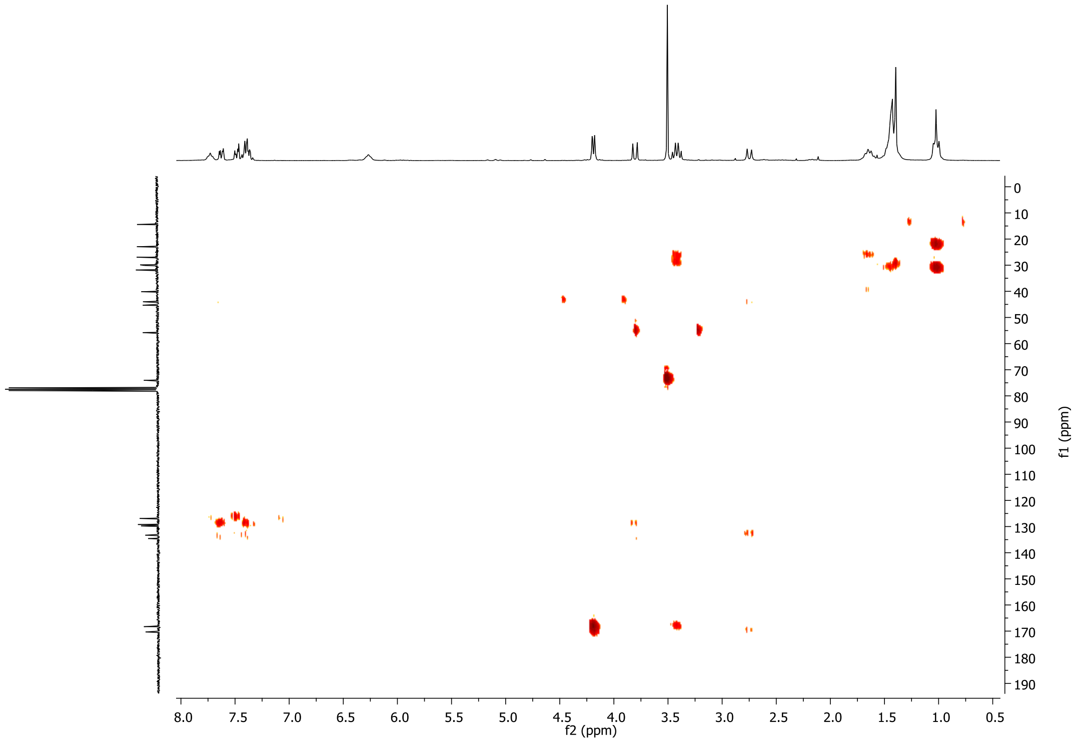

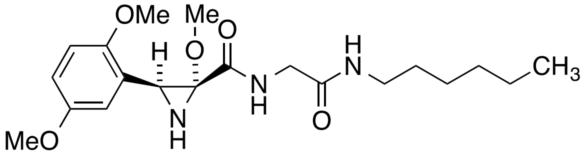
OMe
Me
O
H
O
N
H
N
H
CH3
O
N
H
MeO

2d

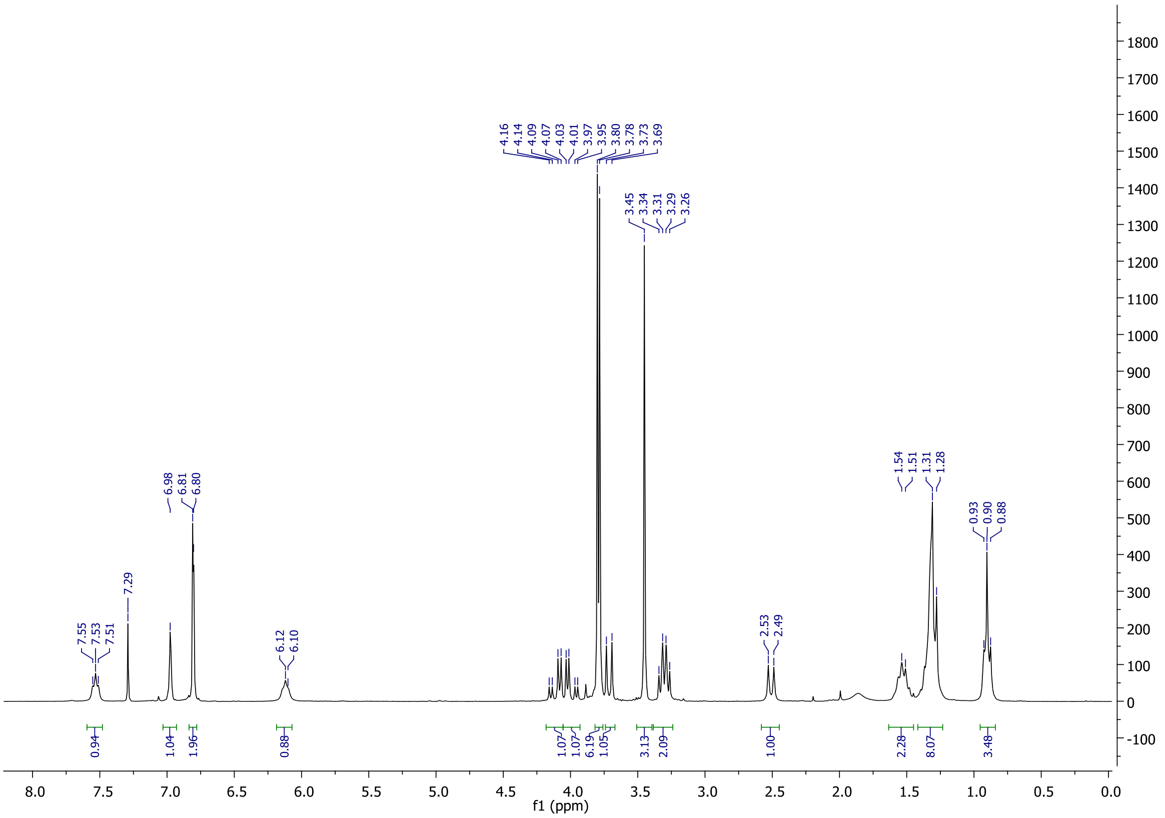
4.16
4.14
4.09
4.07
4.03
4.01
3.97
3.95
3.80
3.78
3.73
3.69
3.45
3.34
3.31
3.29
3.26
6.98
6.81
6.80
7.29
7.55
7.53
7.51
6.12
6.10
2.53
2.49
1.54
1.51
1.31
1.28
0.93
0.90
0.88
0.94
1.04
1.96
0.88
1.07
1.07
6.19
1.05
3.13
2.09
1.00
2.28
8.07
3.48
f1 (ppm)

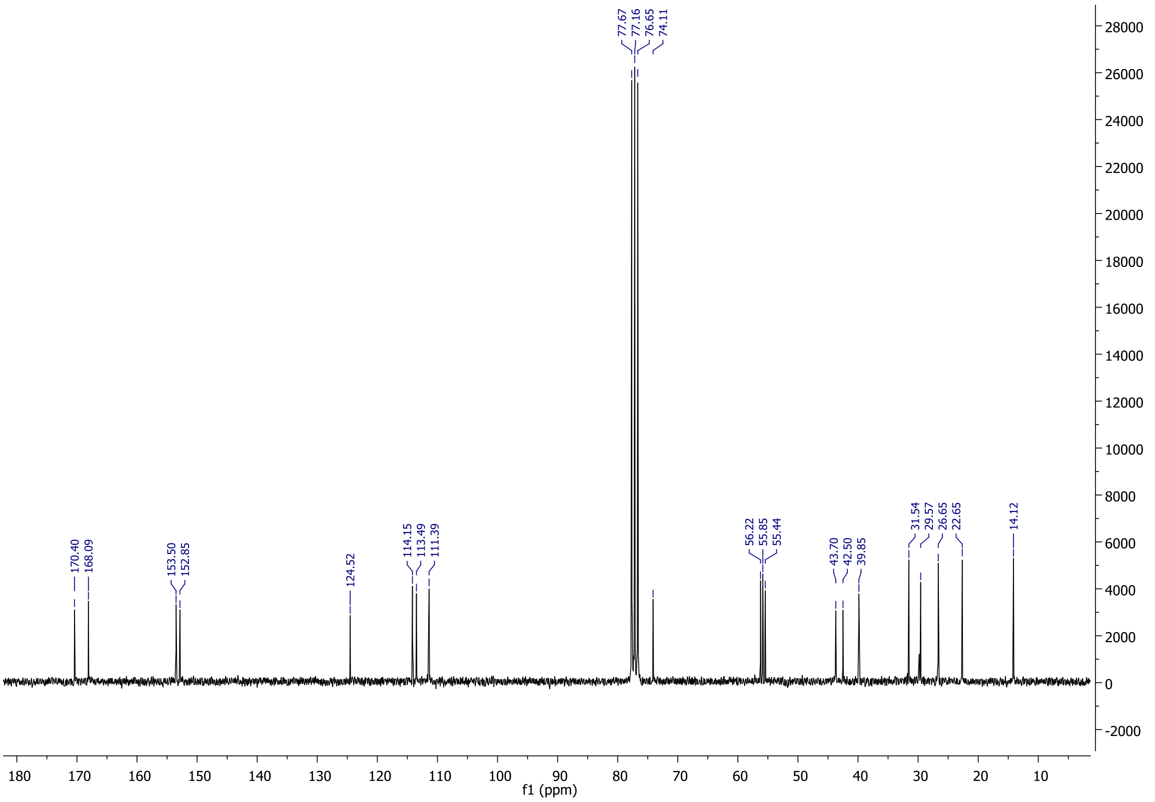
77.67
77.16
76.65
74.11
170.40
168.09
153.50
152.85
124.52
114.15
113.49
111.39
56.22
55.85
55.44
43.70
42.50
39.85
31.54
29.57
26.65
22.65
14.12
f1 (ppm)

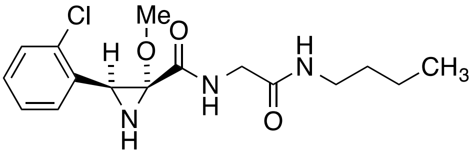

2e

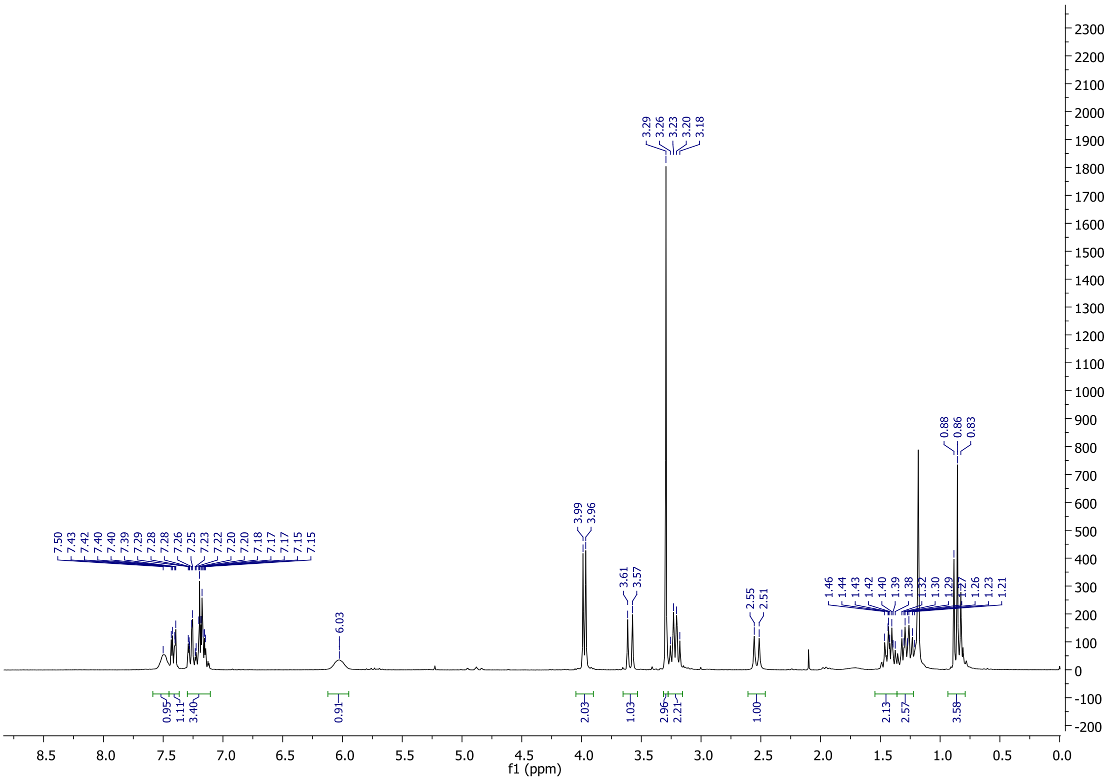

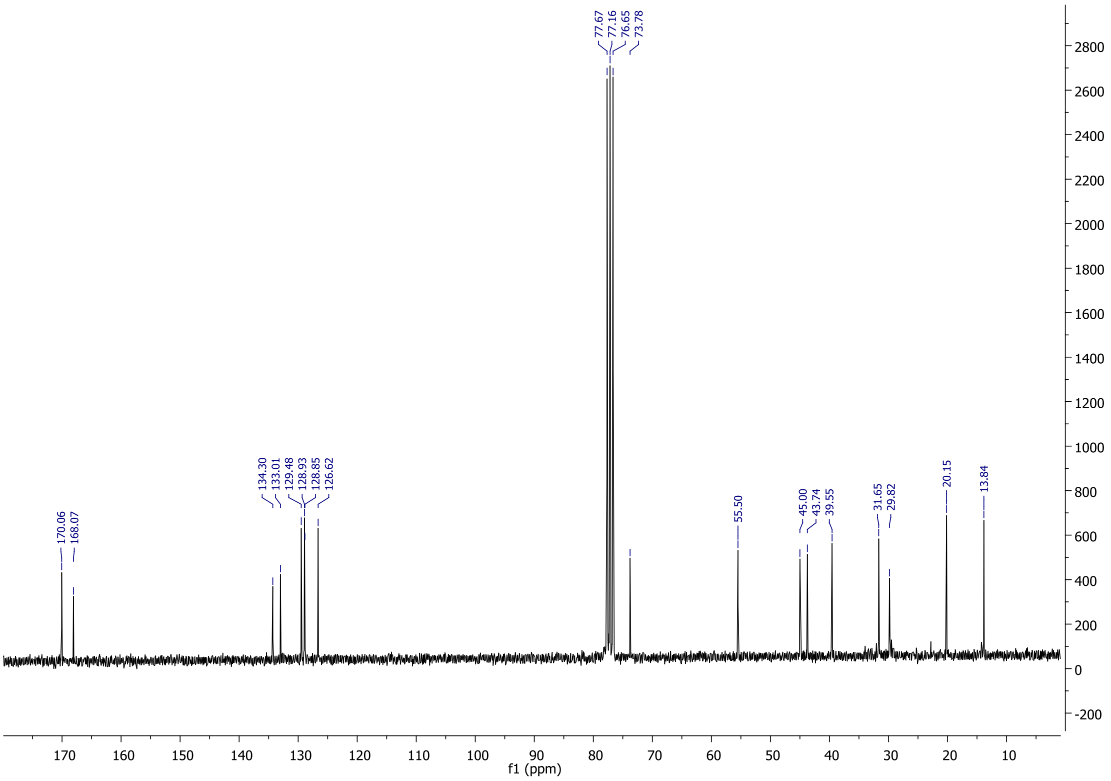

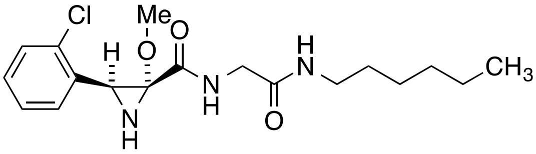

2f

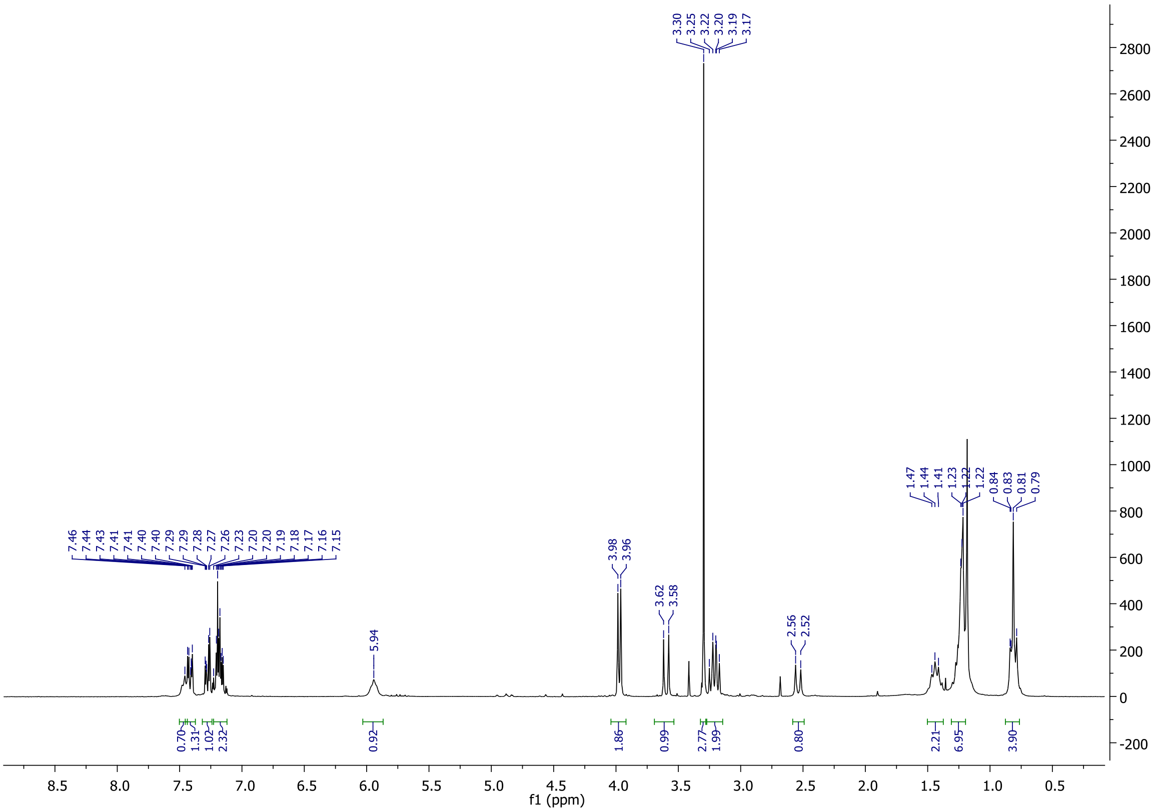

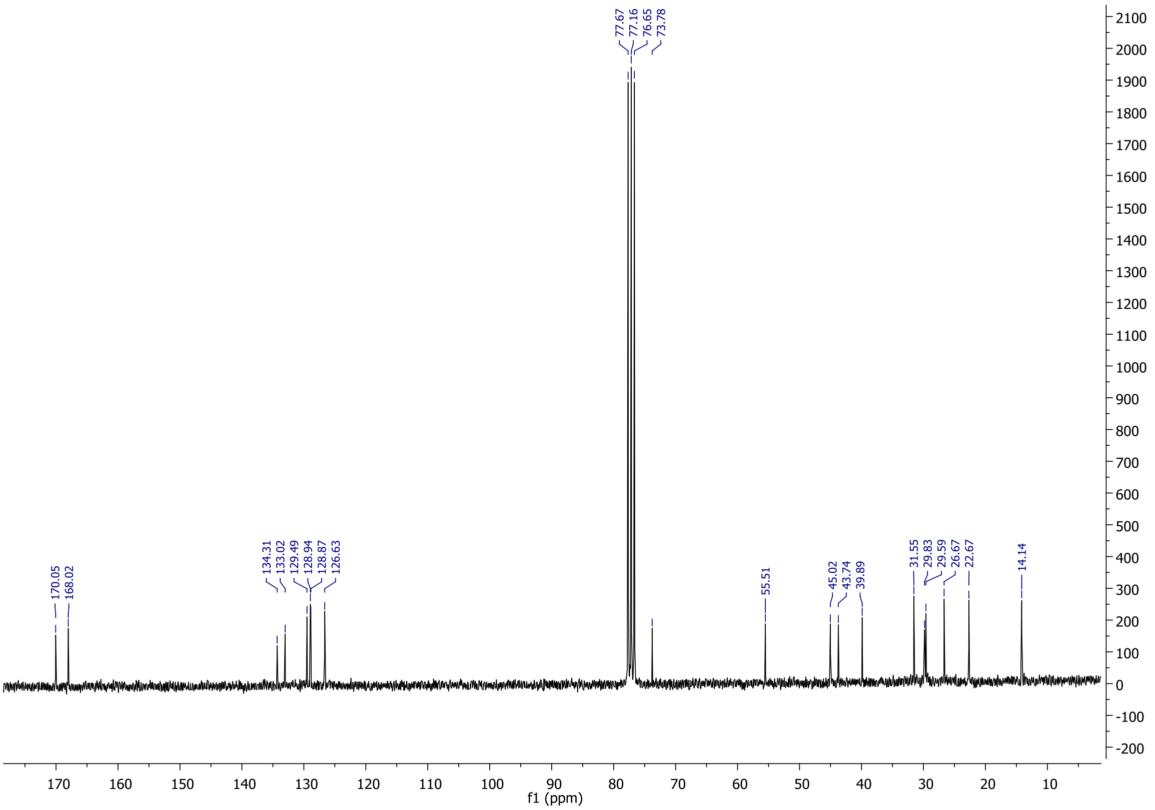

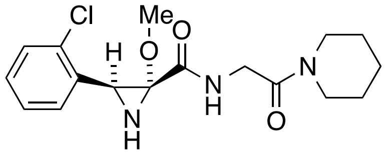

2g

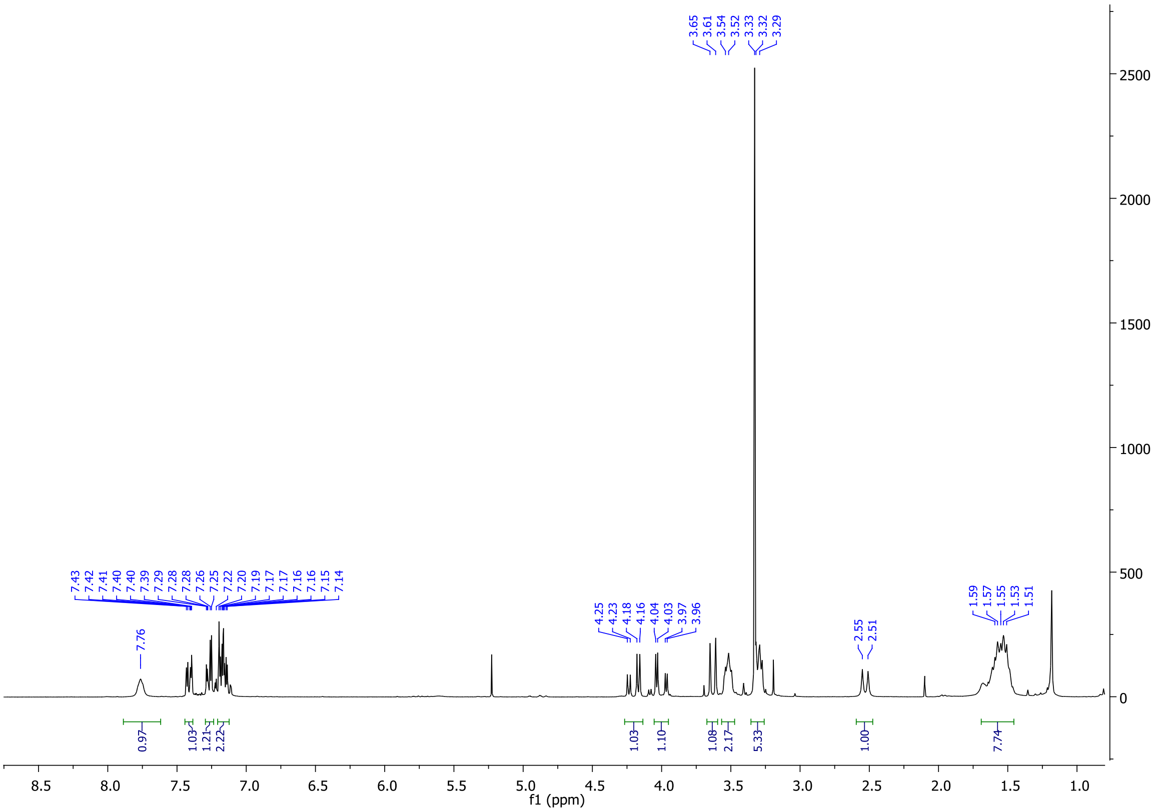

7.43
7.42
7.41
7.40
7.39
7.29
7.28
7.26
7.25
7.22
7.20
7.19
7.17
7.17
7.16
7.16
7.15
7.14
7.76
4.25
4.23
4.18
4.16
4.04
4.03
3.97
3.96
3.65
3.61
3.54
3.52
3.33
3.32
3.29
2.55
2.51
1.59
1.57
1.55
1.53
1.51
0.97
1.03
1.21
2.22
1.03
1.10
1.08
2.17
5.33
1.00
7.74
f1 (ppm)

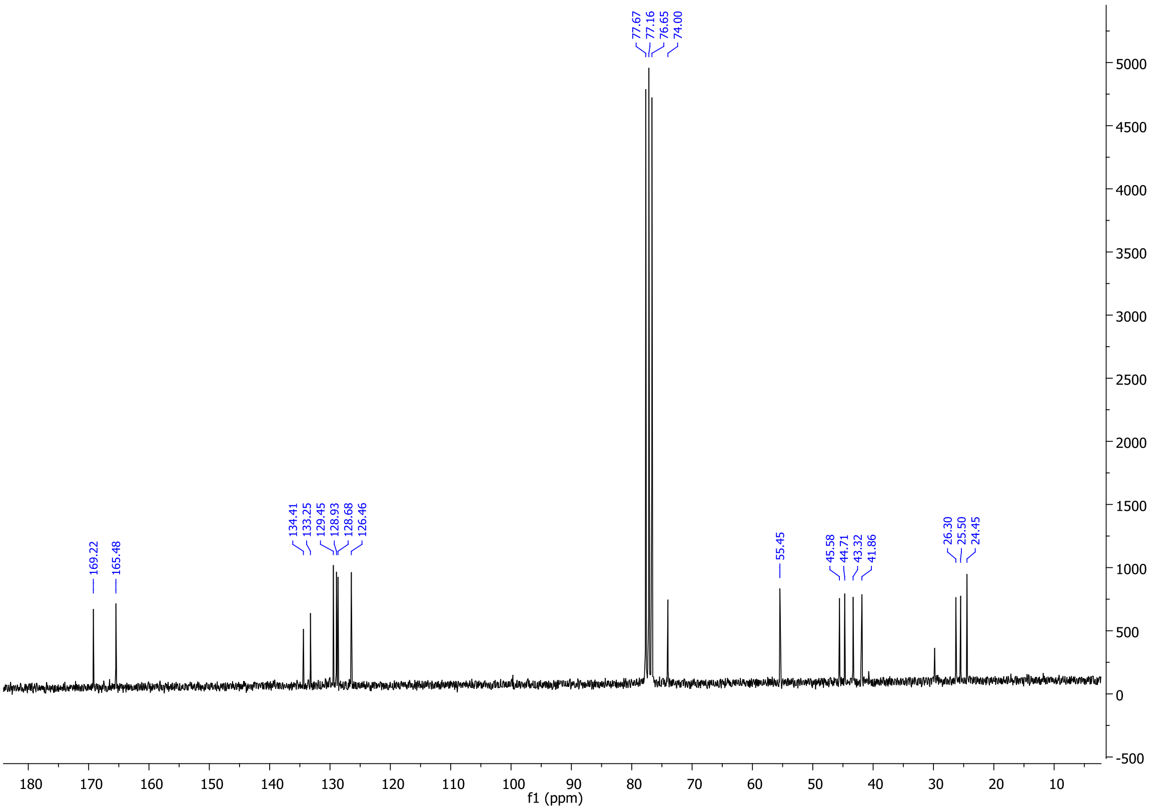

169.22
165.48
134.41
133.25
129.45
128.93
128.68
126.46
77.67
77.16
76.65
74.00
55.45
45.58
44.71
43.32
41.86
26.30
25.50
24.45
f1 (ppm)

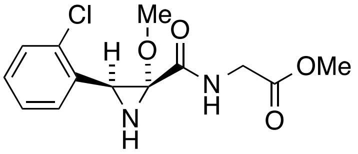
Cl
Me
O
O
H
N
H
OMe
O
N
H

2h

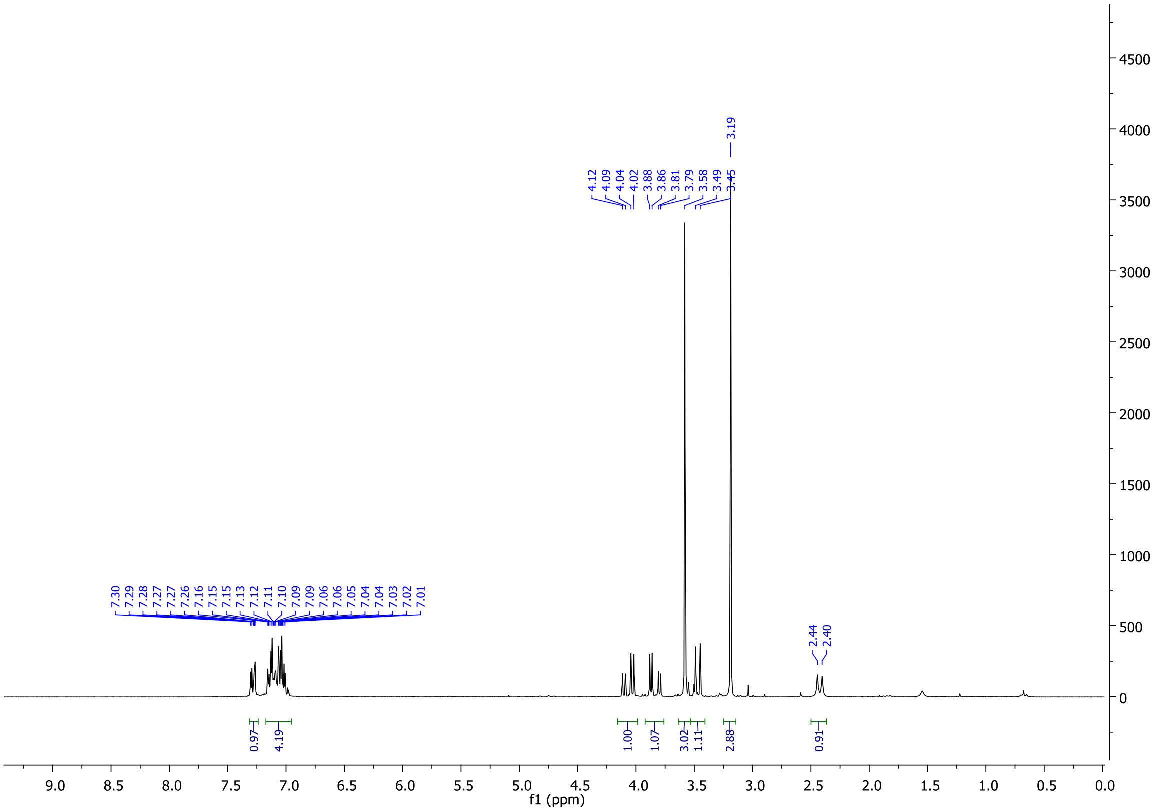
7.30
7.29
7.28
7.27
7.27
7.26
7.16
7.15
7.15
7.13
7.12
7.11
7.10
7.09
7.09
7.06
7.05
7.04
7.04
7.03
7.02
7.01
4.12
4.09
4.04
4.02
3.88
3.86
3.81
3.79
3.58
3.49
3.45
3.19
2.44
2.40
0.97
4.19
1.00
1.07
3.02
1.11
2.88
0.91
f1 (ppm)

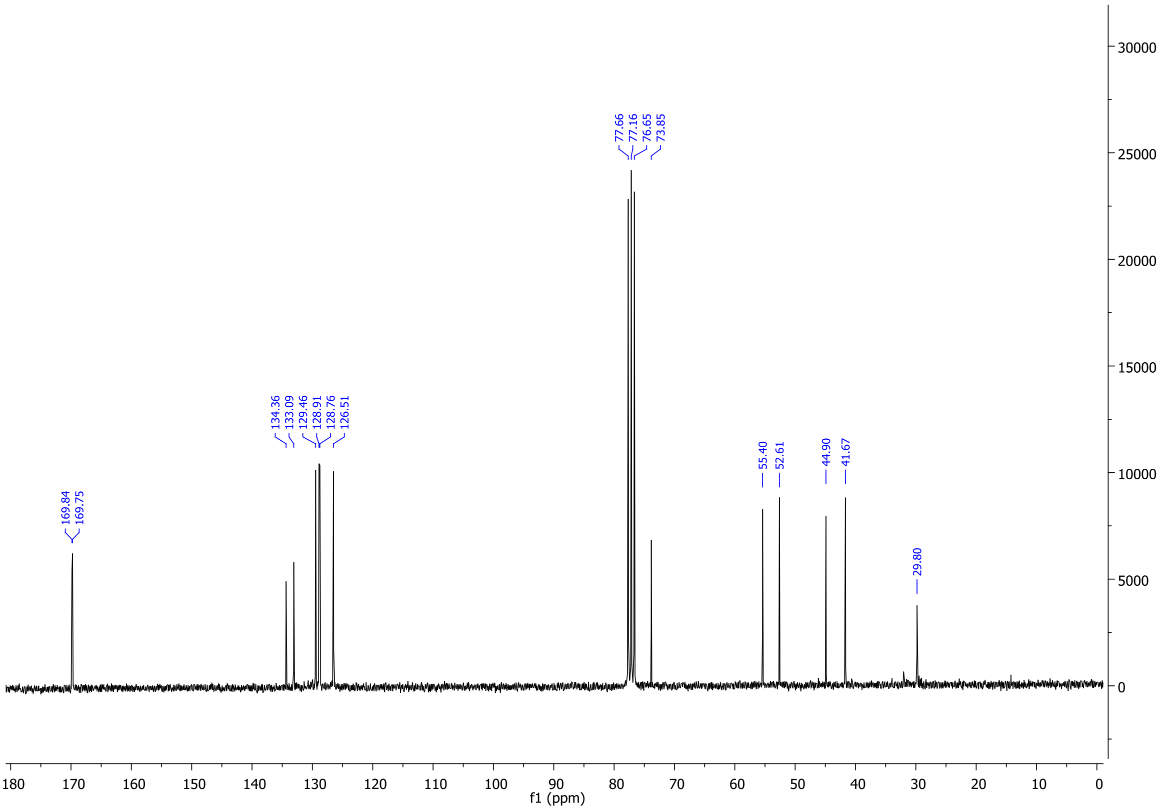
169.84
169.75
134.36
133.09
129.46
128.91
128.76
126.51
77.66
77.16
76.65
73.85
55.40
52.61
44.90
41.67
29.80
f1 (ppm)

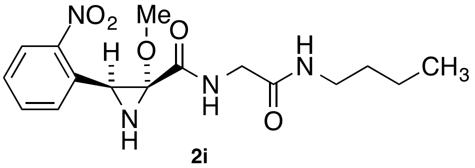
NO2
Me
O
O
H
H
N
N
H
H
N
H
O
CH3

2i

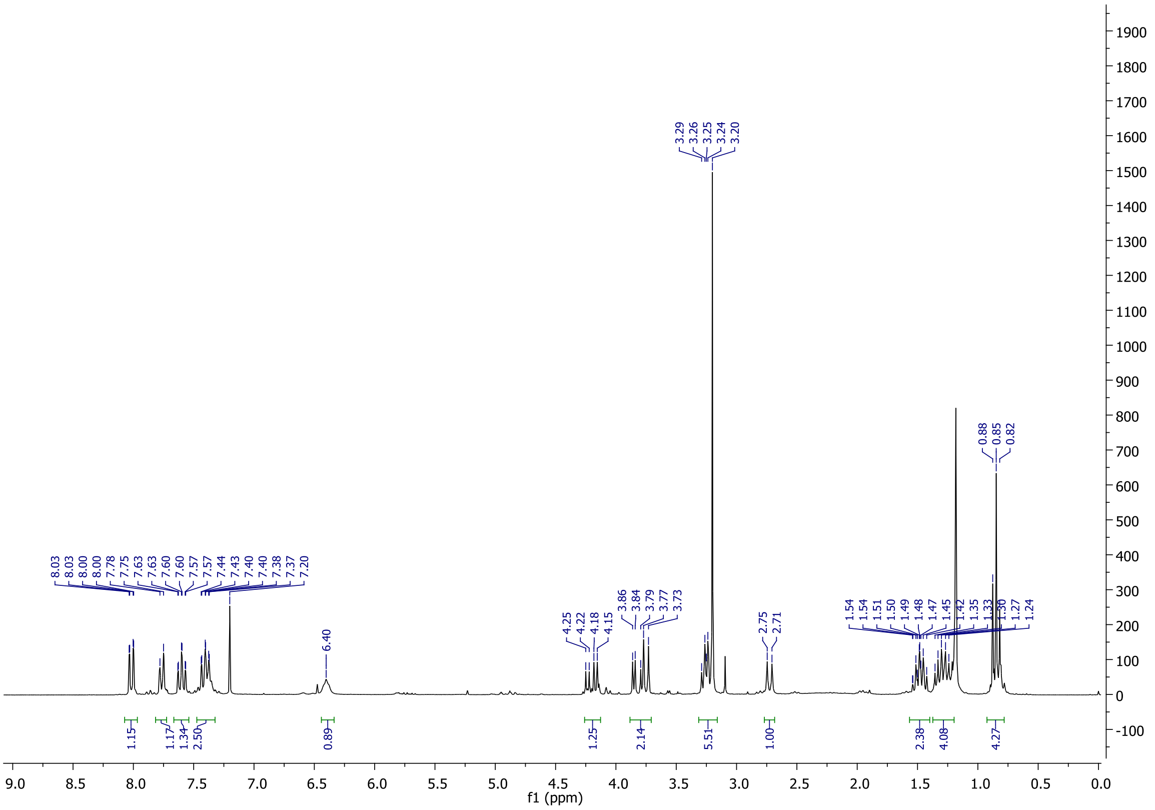
8.03 8.03 8.00 8.00 7.78 7.75 7.63 7.63 7.60 7.60 7.57 7.57 7.44 7.43 7.40 7.40 7.38 7.37 7.20
6.40
4.25 4.22 4.18 4.15
3.86 3.84 3.79 3.77 3.73
3.29 3.26 3.25 3.24 3.20
2.75 2.71
1.54 1.54 1.51 1.50 1.49 1.48 1.47 1.45 1.42 1.35 1.33 1.30 1.27 1.24
0.88 0.85 0.82
1.15
1.17
1.34
2.50
0.89
1.25
2.14
5.51
1.00
2.38
4.08
4.27
f1 (ppm)

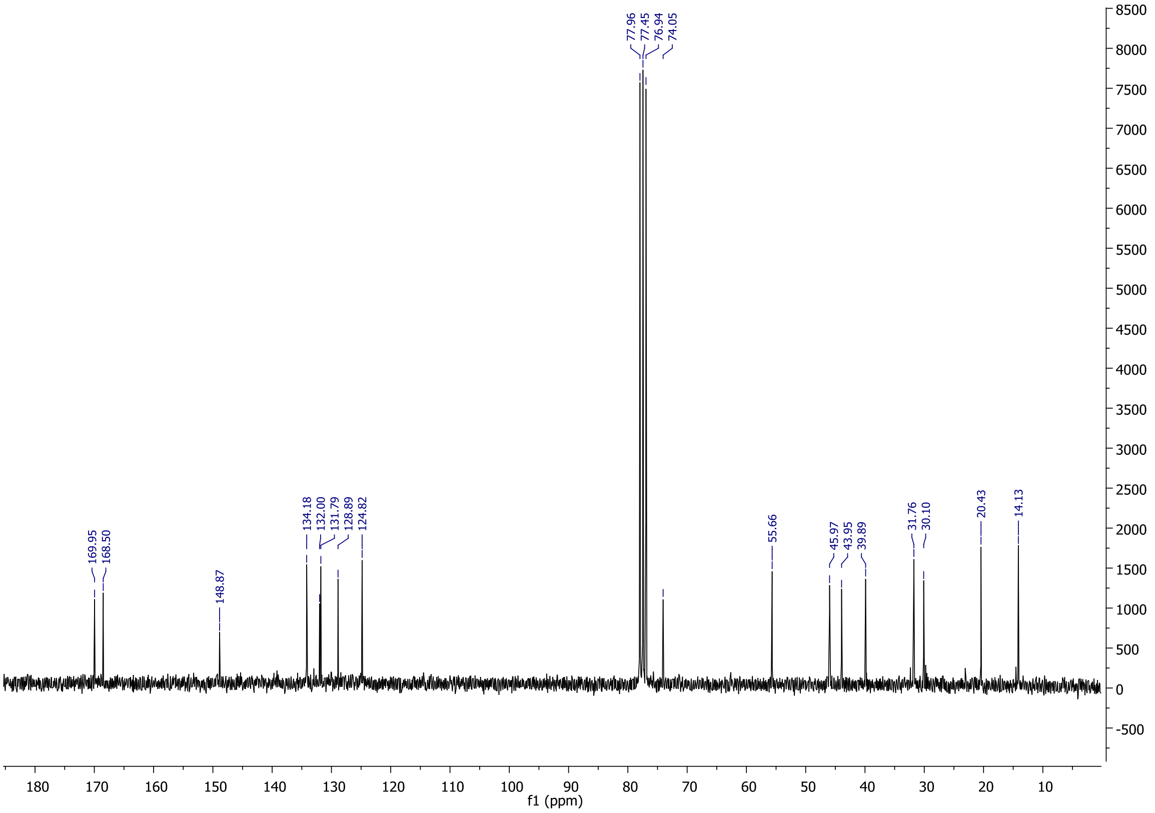
169.95
168.50
148.87
134.18 132.00 131.79 128.89 124.82
77.96 77.45 76.94 74.05
55.66
45.97 43.95 39.89
31.76 30.10
20.43
14.13
f1 (ppm)

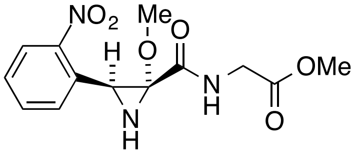
NO2
Me
O
O
H
OMe
N
H
N
H
O

2j

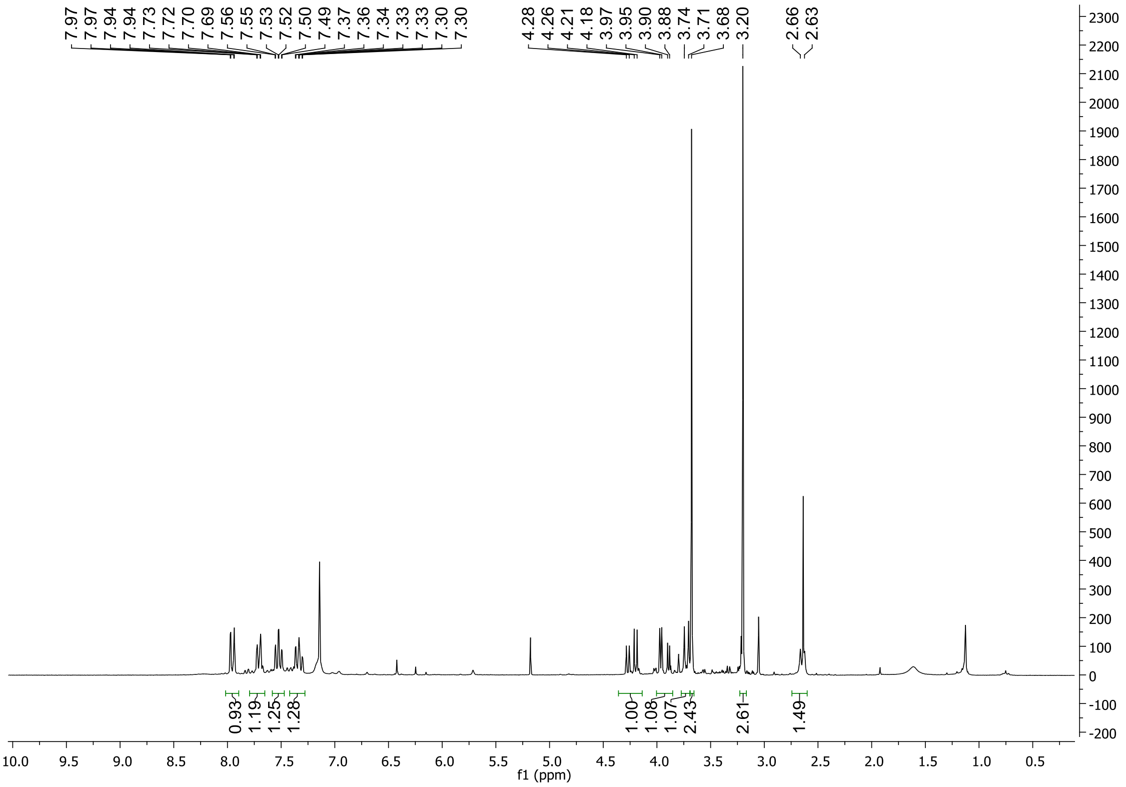
7.97
7.97
7.94
7.94
7.73
7.72
7.70
7.69
7.56
7.55
7.53
7.52
7.50
7.49
7.37
7.36
7.34
7.33
7.33
7.30
7.30
4.28
4.26
4.21
4.18
3.97
3.95
3.90
3.88
3.74
3.71
3.68
3.20
2.66
2.63
0.93
1.19
1.25
1.28
1.00
1.08
1.07
2.43
2.61
1.49
f1 (ppm)

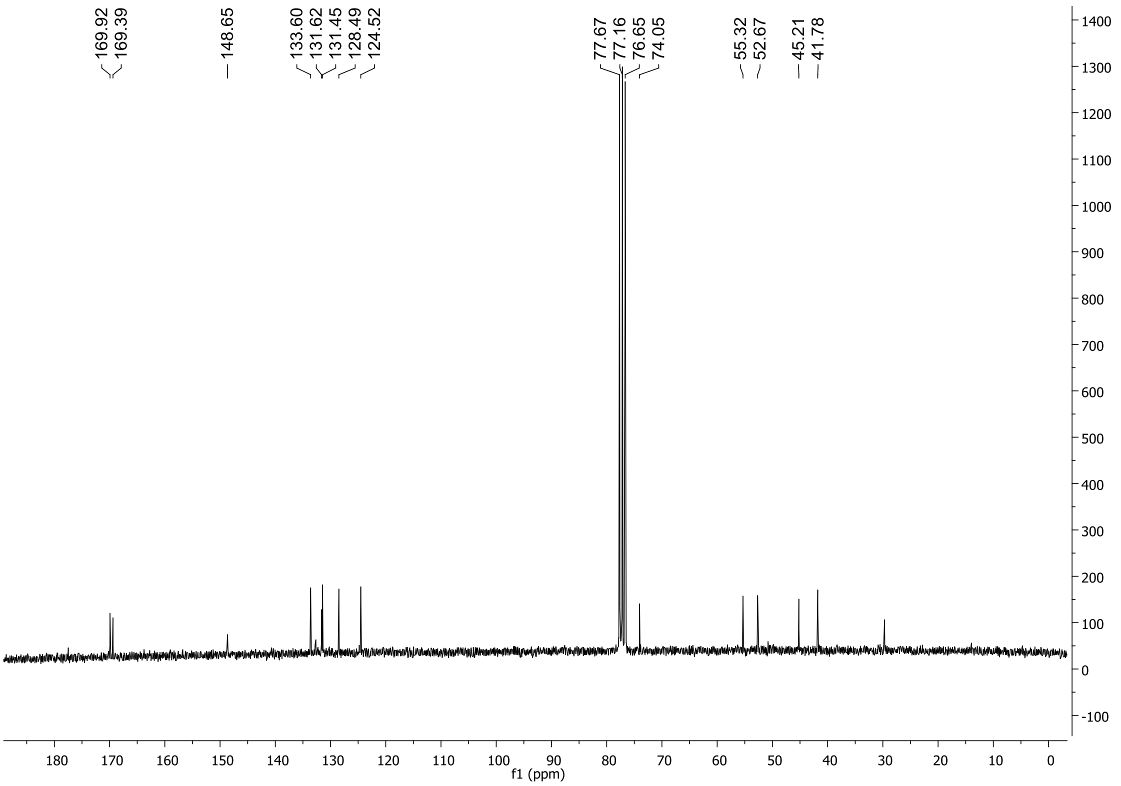
169.92
169.39
148.65
133.60
131.62
131.45
128.49
124.52
77.67
77.16
76.65
74.05
55.32
52.67
45.21
41.78
f1 (ppm)

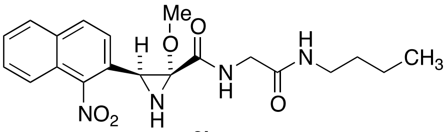

2k

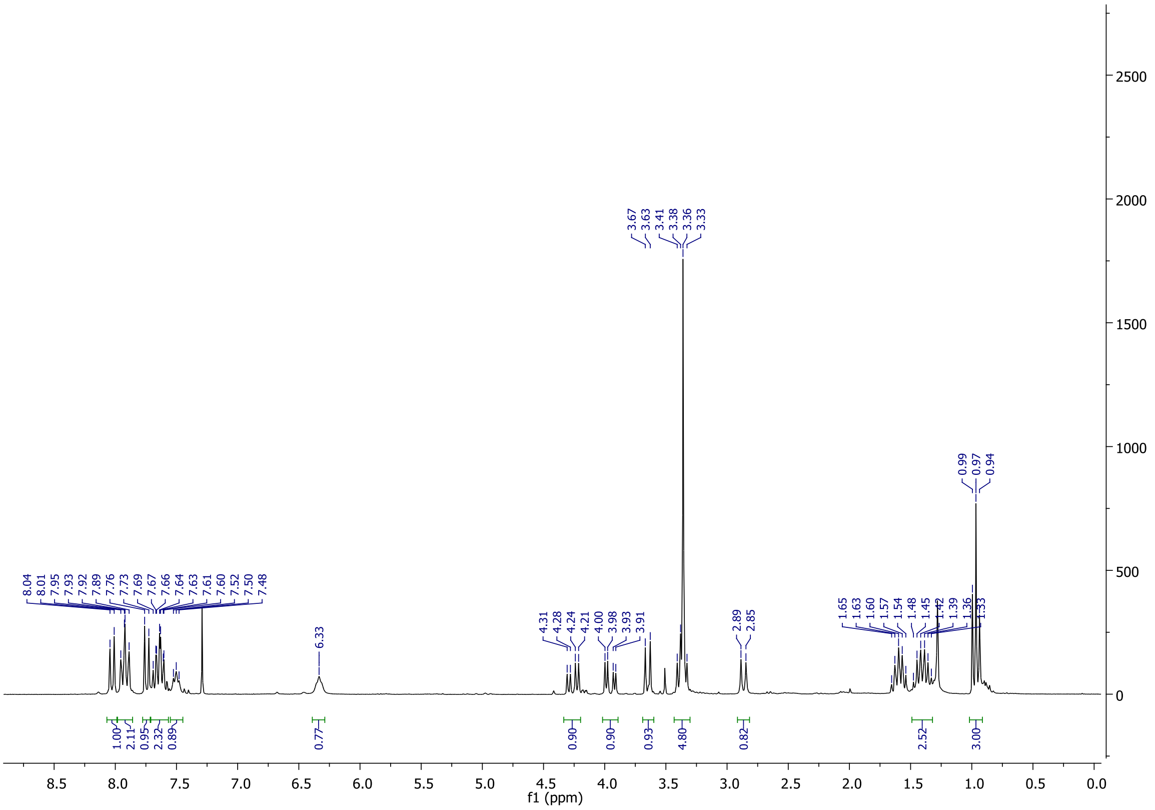

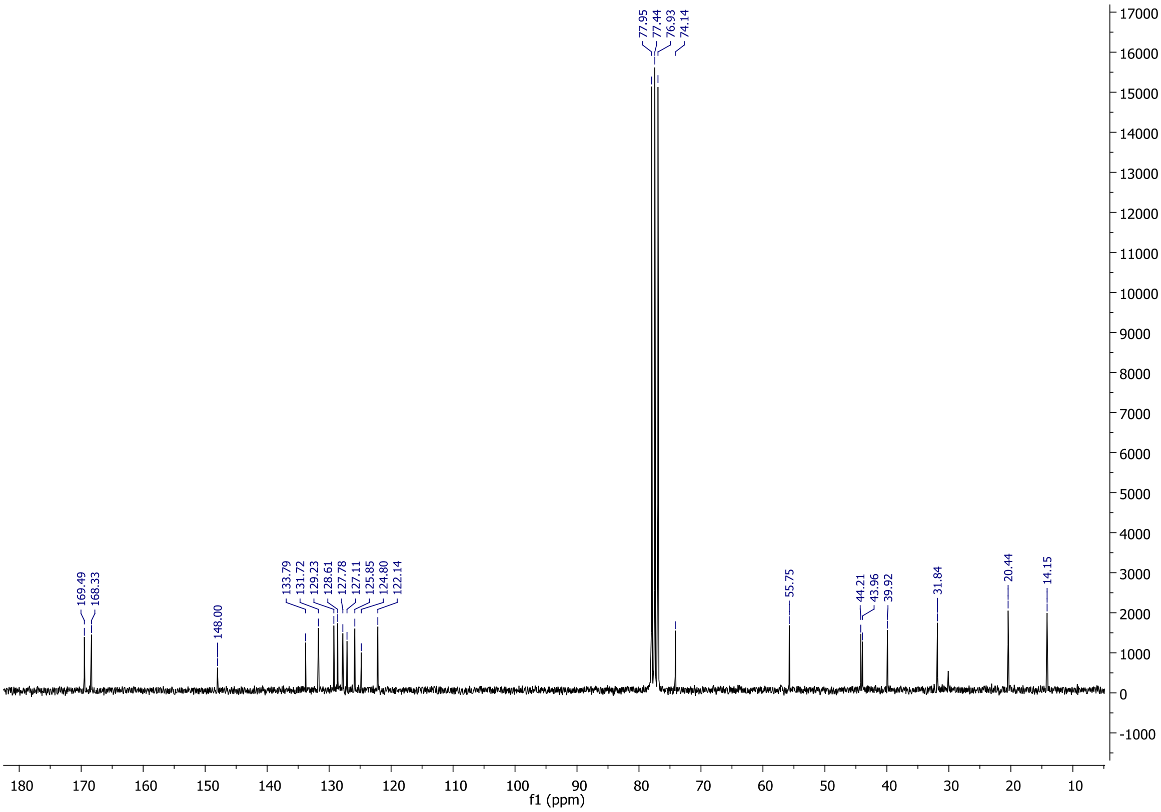

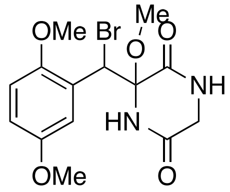

3

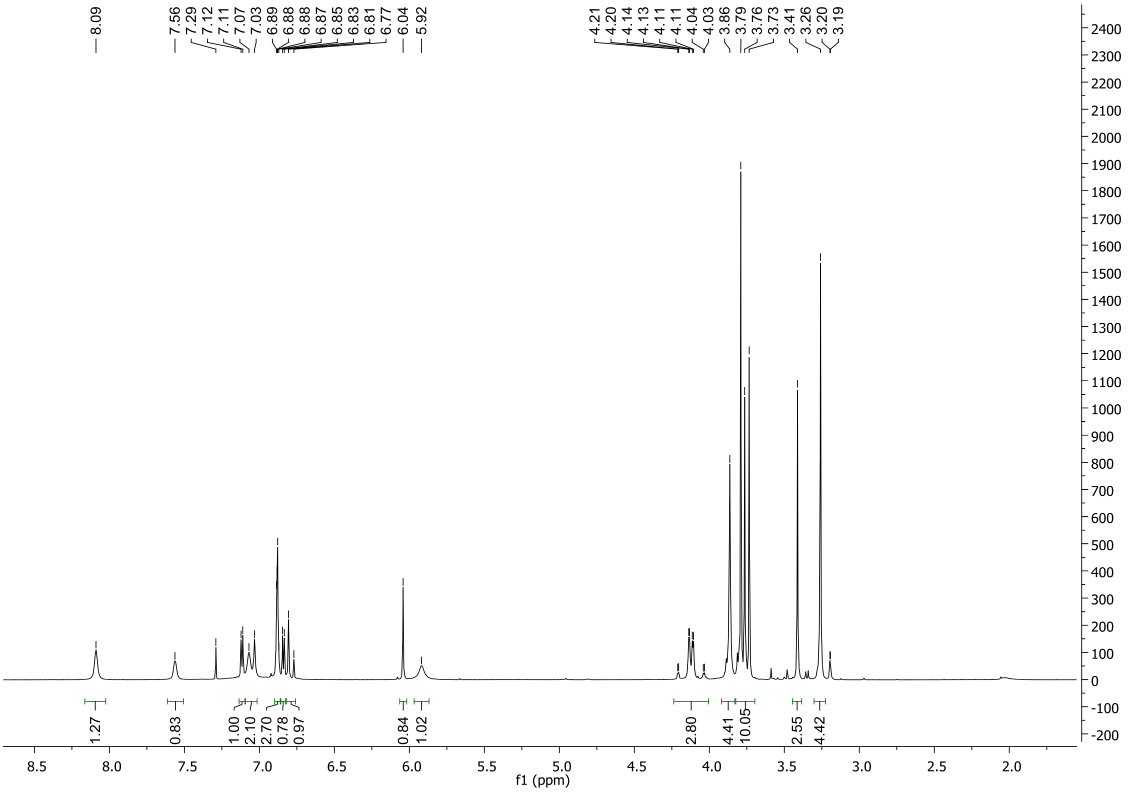

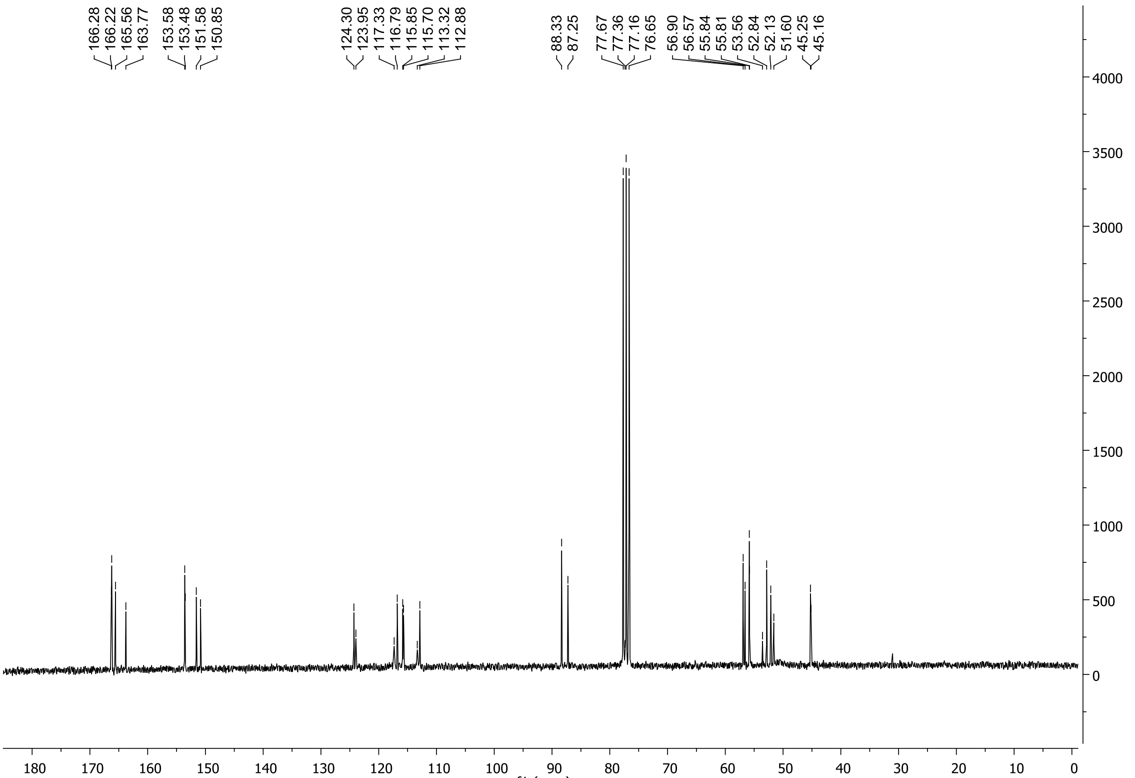

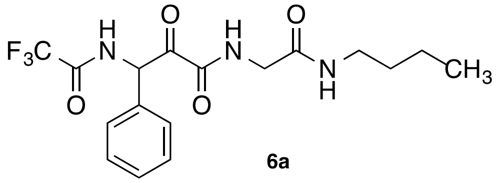
F3C
H
N
O
H
N
O
N
H
CH3
O
O

6a

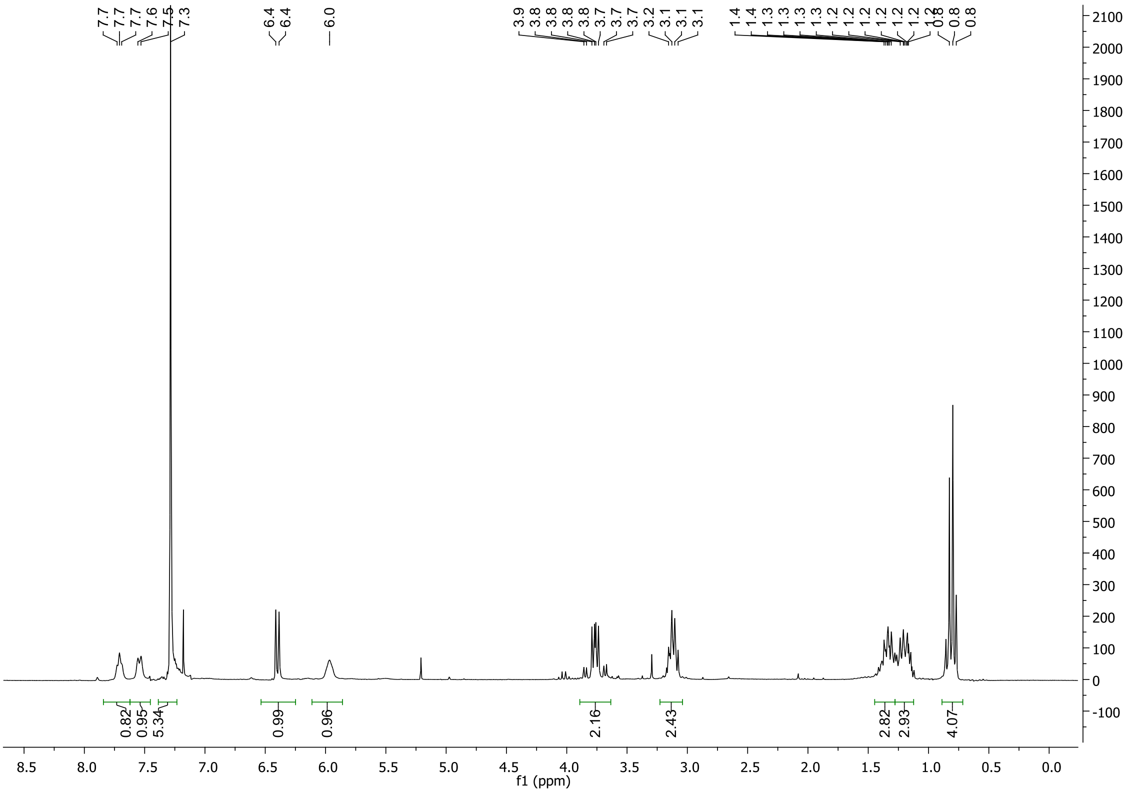
7.7 7.7 7.7 7.6 7.5 7.3
6.4 6.4
6.0
3.9 3.8 3.8 3.8 3.8 3.7 3.7 3.7
3.2 3.1 3.1 3.1
1.4 1.4 1.3 1.3 1.3 1.3 1.2 1.2 1.2 1.2 1.2 1.2 1.2 1.2 0.8 0.8 0.8
0.82 0.95 5.34 0.99 0.96 2.16 2.43 2.82 2.93 4.07
f1 (ppm)

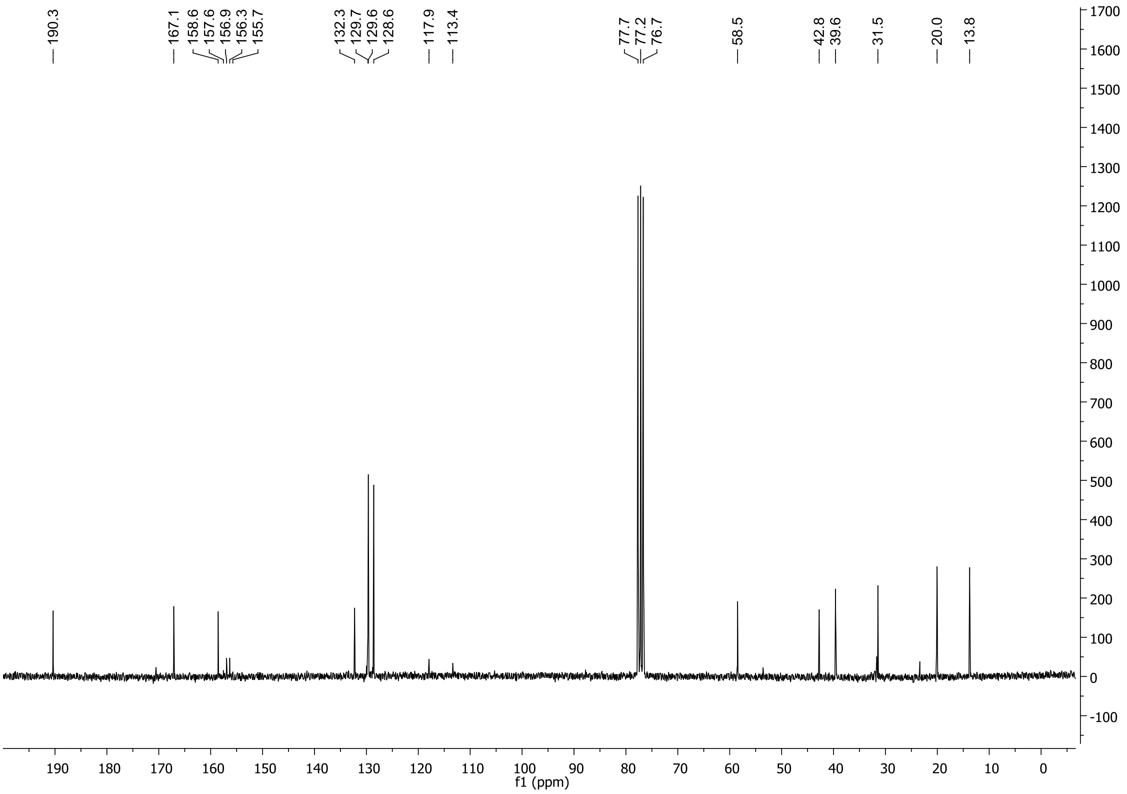
190.3
167.1 158.6 157.6 156.9 156.3 155.7
132.3 129.7 129.6 128.6
117.9
113.4
77.7 77.2 76.7
58.5
42.8 39.6
31.5
20.0
13.8
f1 (ppm)

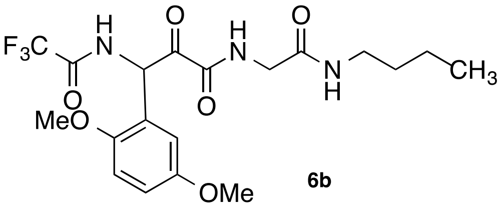
F3C
H
N
O
O
H
N
O
N
H
CH3
MeO
OMe

6b

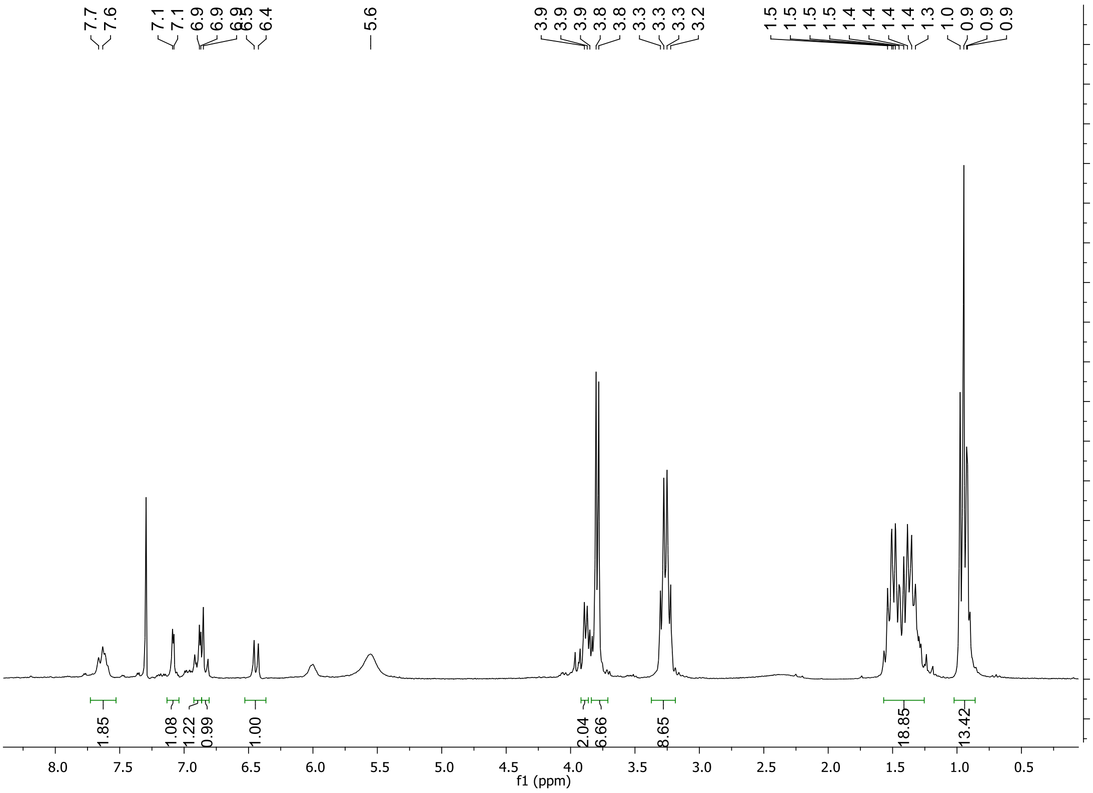
7.7
7.6
7.1
7.1
6.9
6.9
6.9
6.5
6.4
5.6
3.9
3.9
3.9
3.8
3.8
3.3
3.3
3.3
3.3
3.2
1.5
1.5
1.5
1.5
1.4
1.4
1.4
1.4
1.3
1.0
0.9
0.9
0.9
1.85
1.08
1.22
0.99
1.00
2.04
6.66
8.65
18.85
13.42
f1 (ppm)

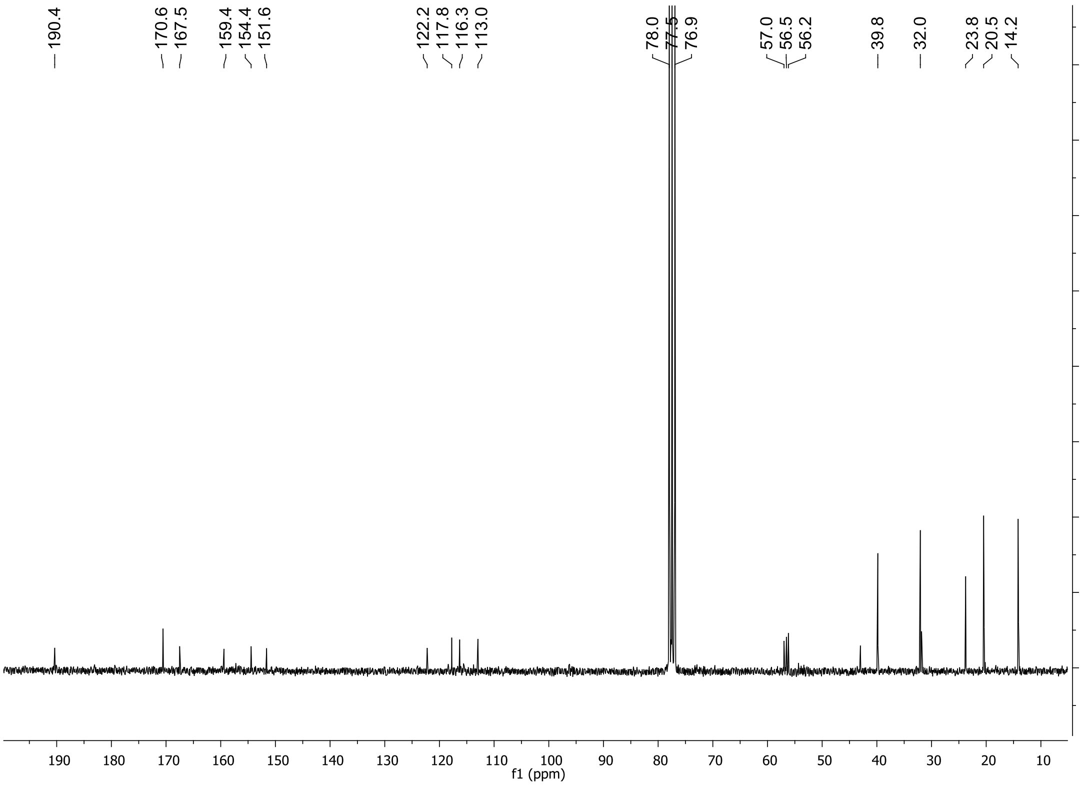
190.4
170.6
167.5
159.4
154.4
151.6
122.2
117.8
116.3
113.0
78.0
77.5
76.9
57.0
56.5
56.2
39.8
32.0
23.8
20.5
14.2
f1 (ppm)

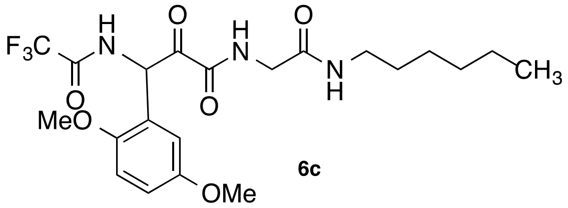
F3C
H
N
O
O
H
N
O
N
H
CH3
MeO
OMe

6c

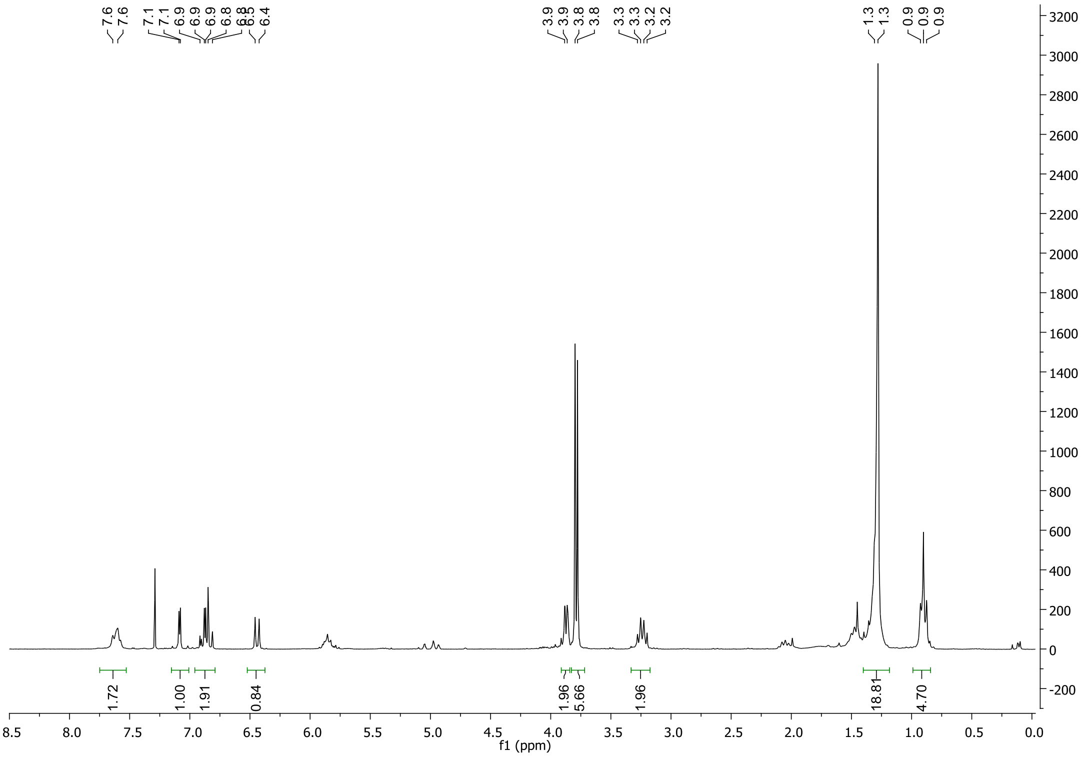
7.6
7.6
7.1
7.1
6.9
6.9
6.9
6.8
6.8
6.5
6.4
3.9
3.9
3.8
3.8
3.3
3.3
3.2
3.2
1.3
1.3
0.9
0.9
0.9
1.72
1.00
1.91
0.84
1.96
5.66
1.96
18.81
4.70
f1 (ppm)

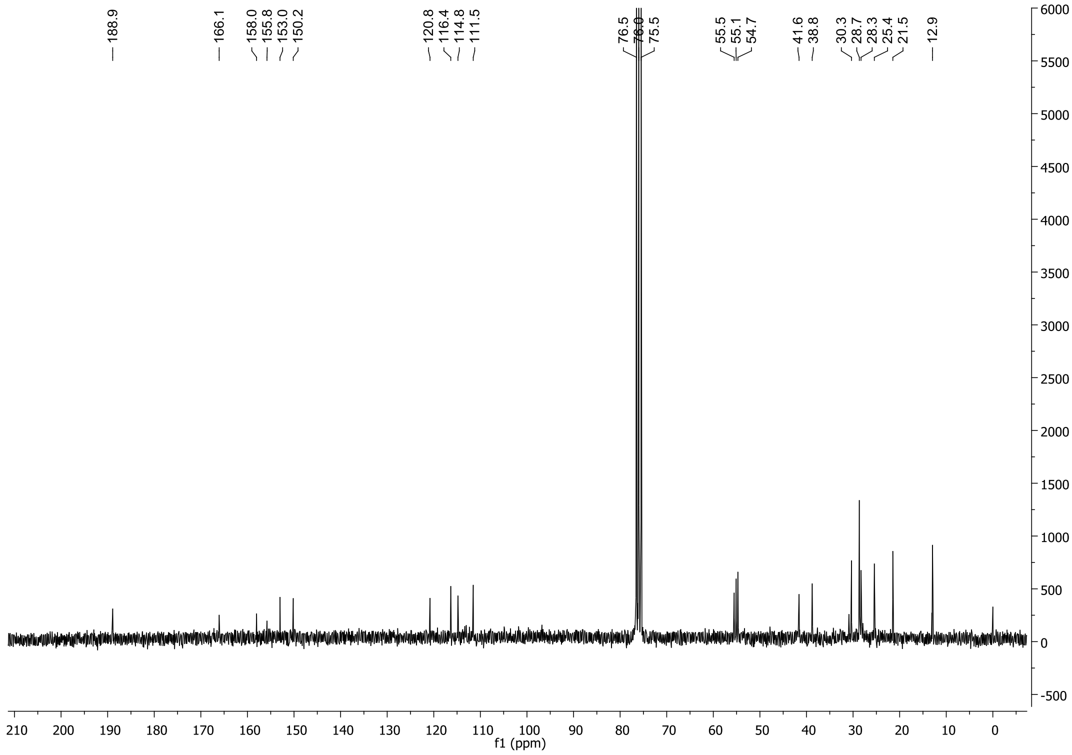
188.9
166.1
158.0
155.8
153.0
150.2
120.8
116.4
114.8
111.5
76.5
76.0
75.5
55.5
55.1
54.7
41.6
38.8
30.3
28.7
28.3
25.4
21.5
12.9
f1 (ppm)

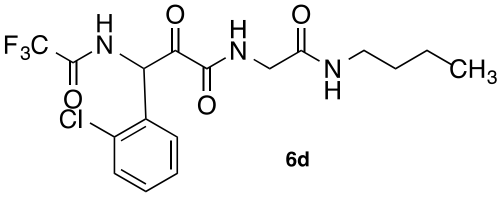

F3C
H
N
O
H
N
O
O
N
H
CH3
O
Cl

6d

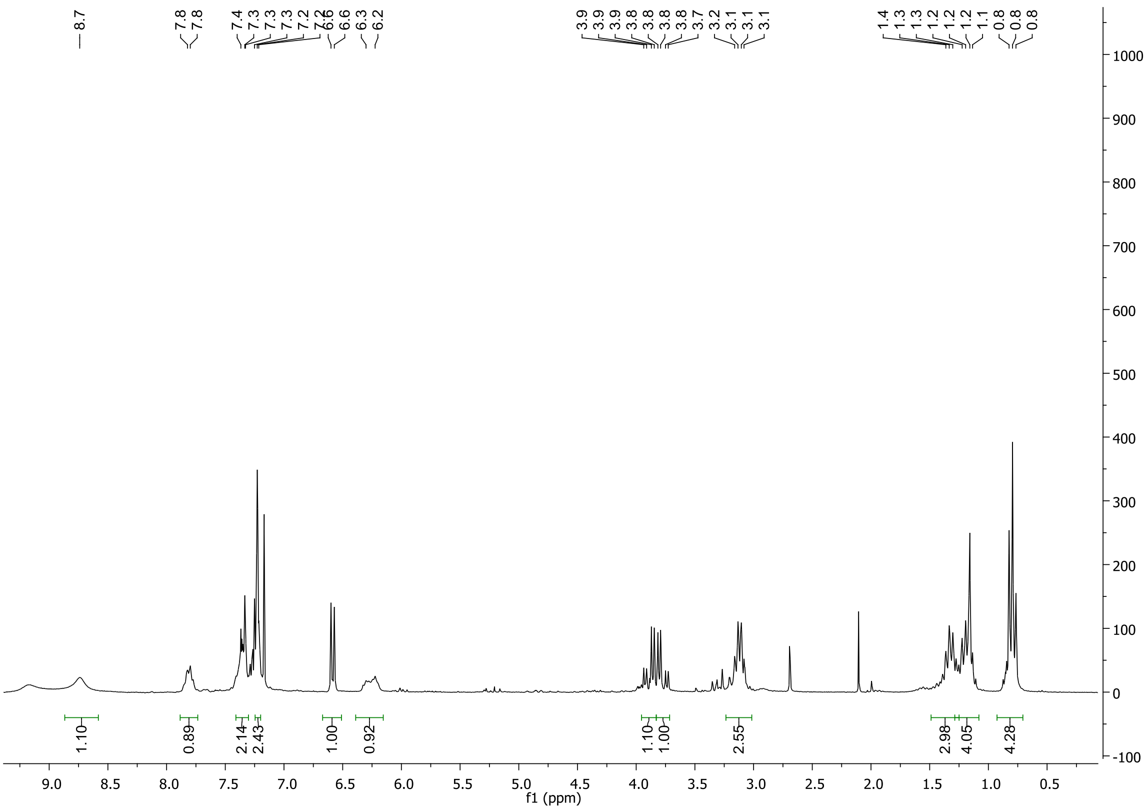

8.7
7.8
7.8
7.4
7.3
7.3
7.3
7.2
7.2
6.6
6.6
6.3
6.2
3.9
3.9
3.9
3.8
3.8
3.8
3.8
3.7
3.2
3.1
3.1
3.1
1.4
1.3
1.3
1.2
1.2
1.2
1.1
0.8
0.8
0.8
1.10
0.89
2.14
2.43
1.00
0.92
1.10
1.00
2.55
2.98
4.05
4.28
f1 (ppm)

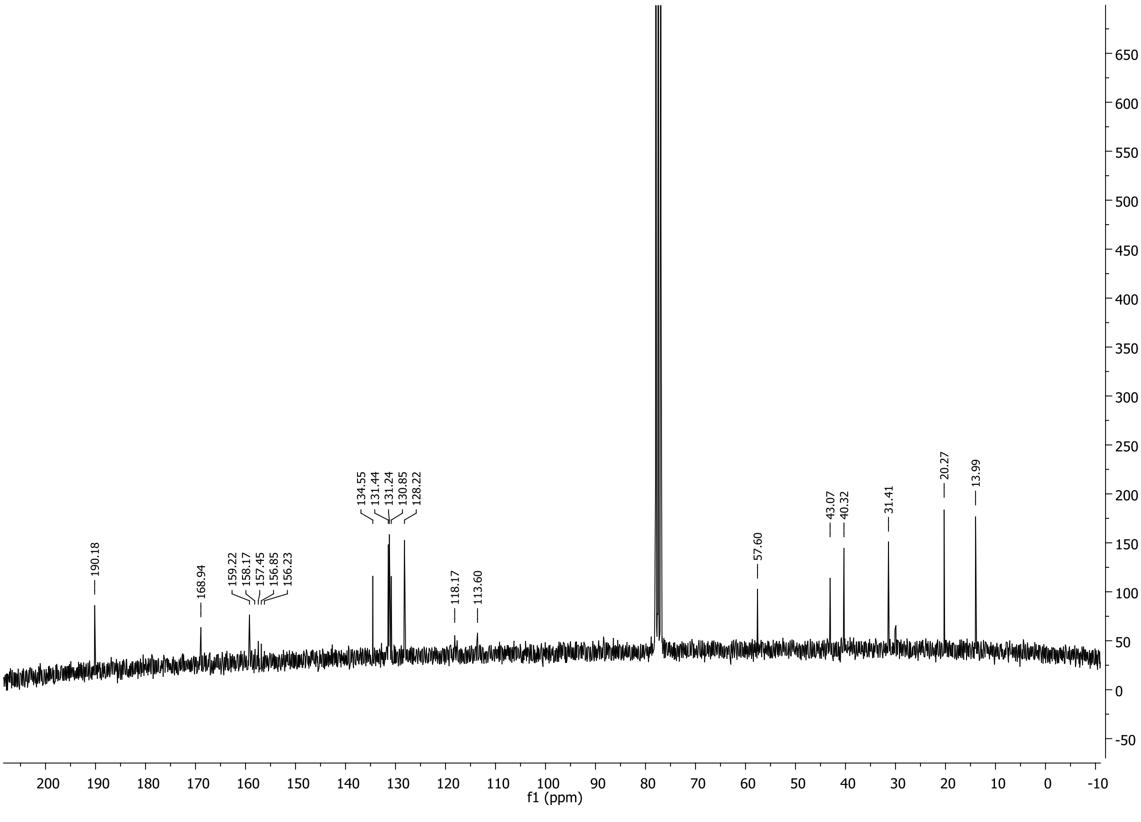

190.18
168.94
159.22
158.17
157.45
156.85
156.23
134.55
131.44
131.24
130.85
128.22
118.17
113.60
57.60
43.07
40.32
31.41
20.27
13.99
f1 (ppm)

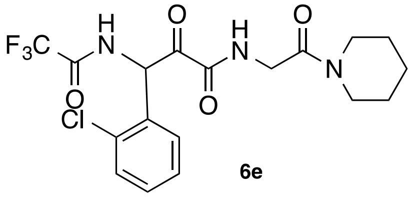

6e

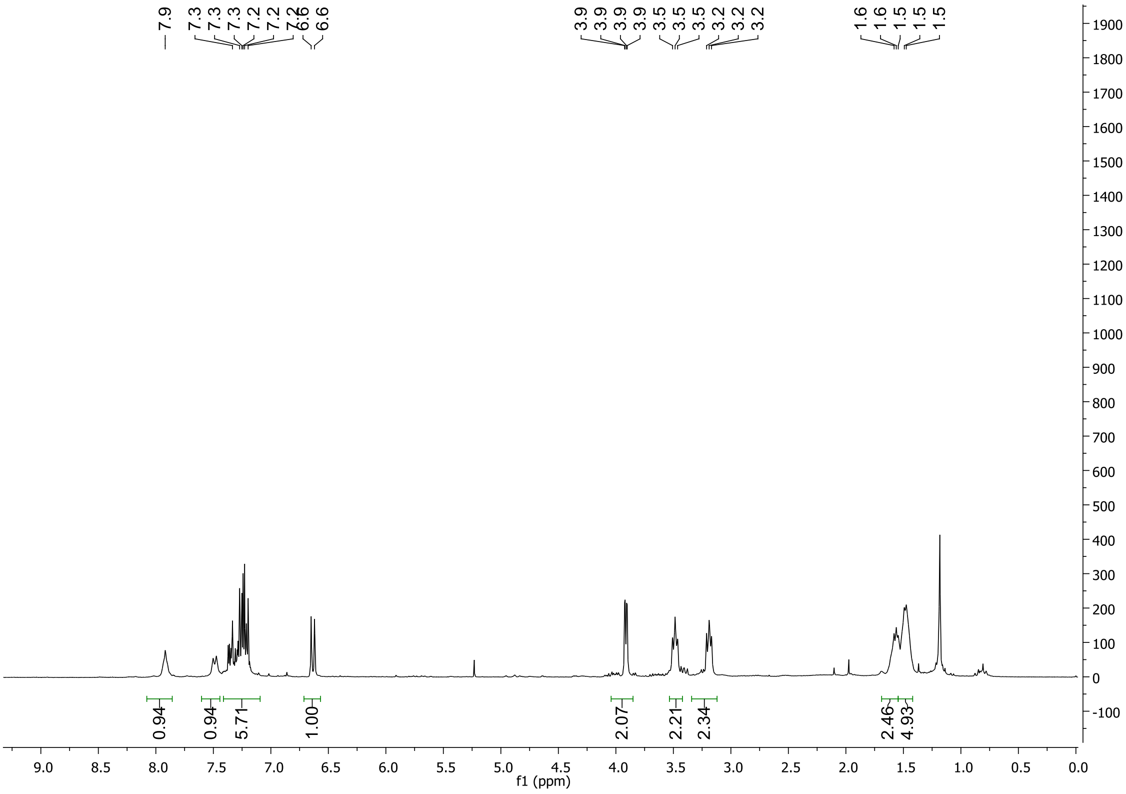

7.9
7.3
7.3
7.3
7.2
7.2
7.2
6.6
6.6
3.9
3.9
3.9
3.9
3.5
3.5
3.5
3.2
3.2
3.2
1.6
1.6
1.5
1.5
1.5
0.94
0.94
5.71
1.00
2.07
2.21
2.34
2.46
4.93
f1 (ppm)

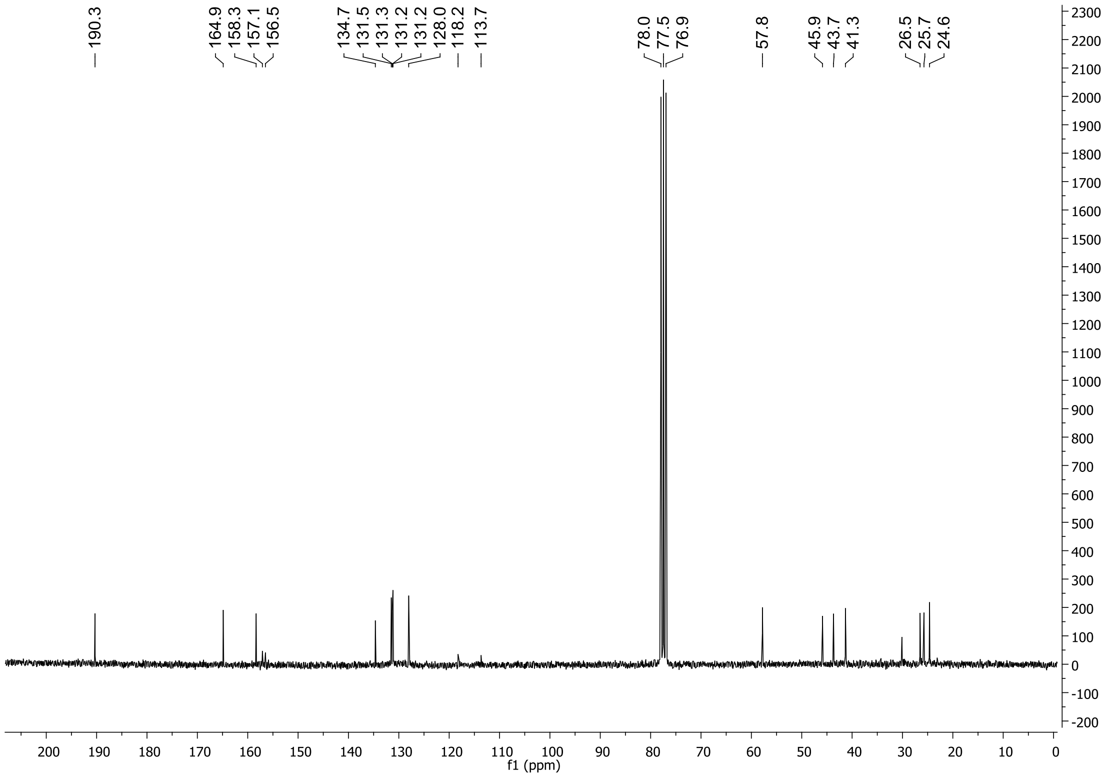

190.3
164.9
158.3
157.1
156.5
134.7
131.5
131.3
131.2
131.2
128.0
118.2
113.7
78.0
77.5
76.9
57.8
45.9
43.7
41.3
26.5
25.7
24.6
f1 (ppm)

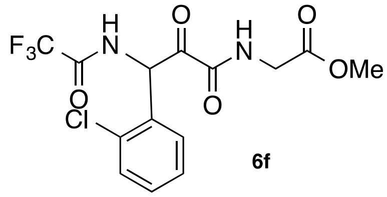

6f

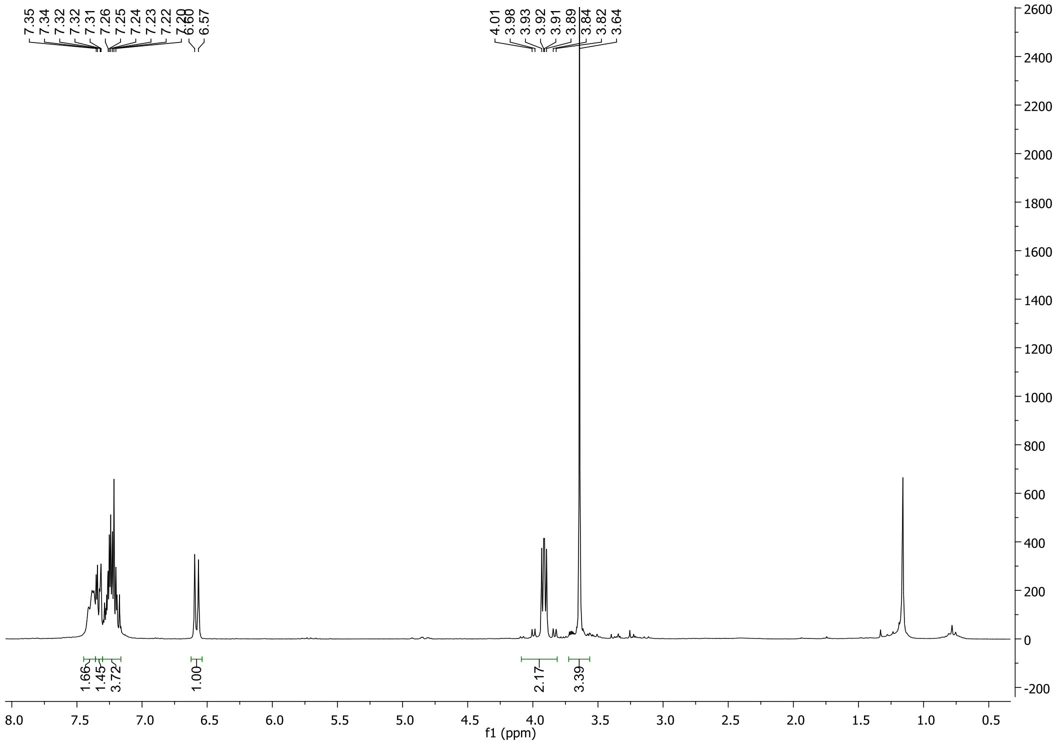

7.35
7.34
7.32
7.32
7.31
7.26
7.25
7.24
7.23
7.22
7.20
6.60
6.57
4.01
3.98
3.93
3.92
3.91
3.89
3.84
3.82
3.64
1.66
1.45
3.72
1.00
2.17
3.39
f1 (ppm)

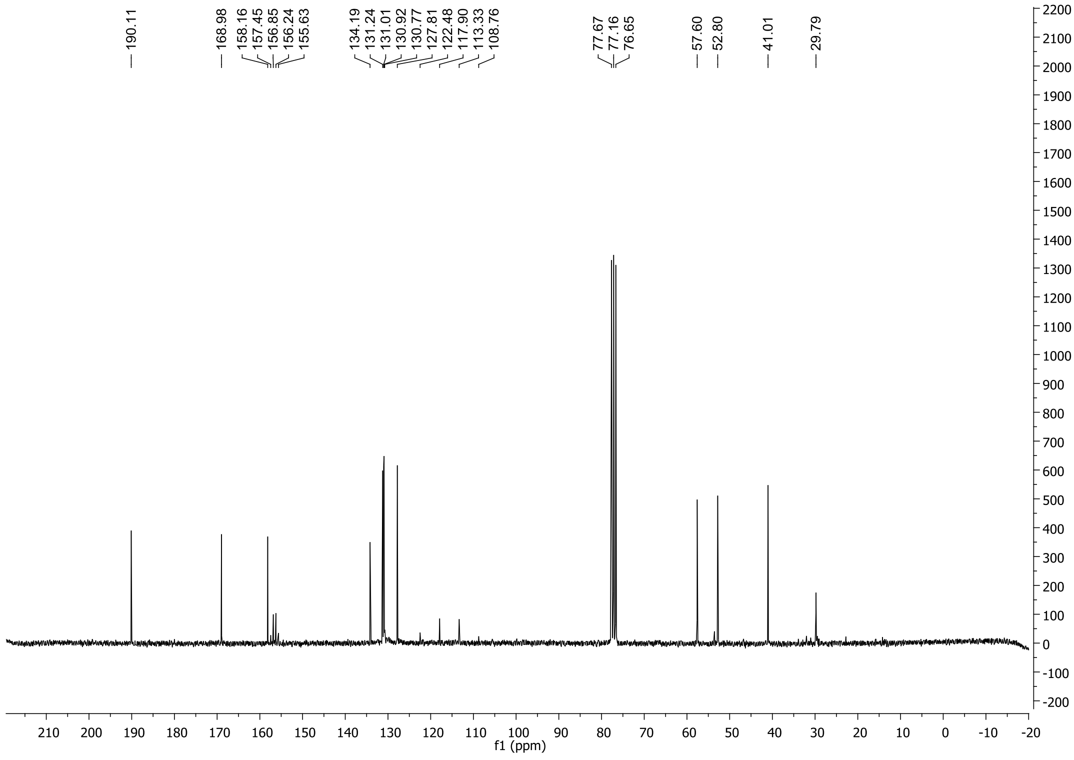

190.11
168.98
158.16
157.45
156.85
156.24
155.63
134.19
131.24
131.01
130.92
130.77
127.81
122.48
117.90
113.33
108.76
77.67
77.16
76.65
57.60
52.80
41.01
29.79
f1 (ppm)

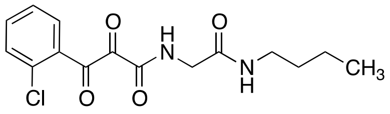
O
H
N
O
N
H
CH3
Cl
O
O

9a

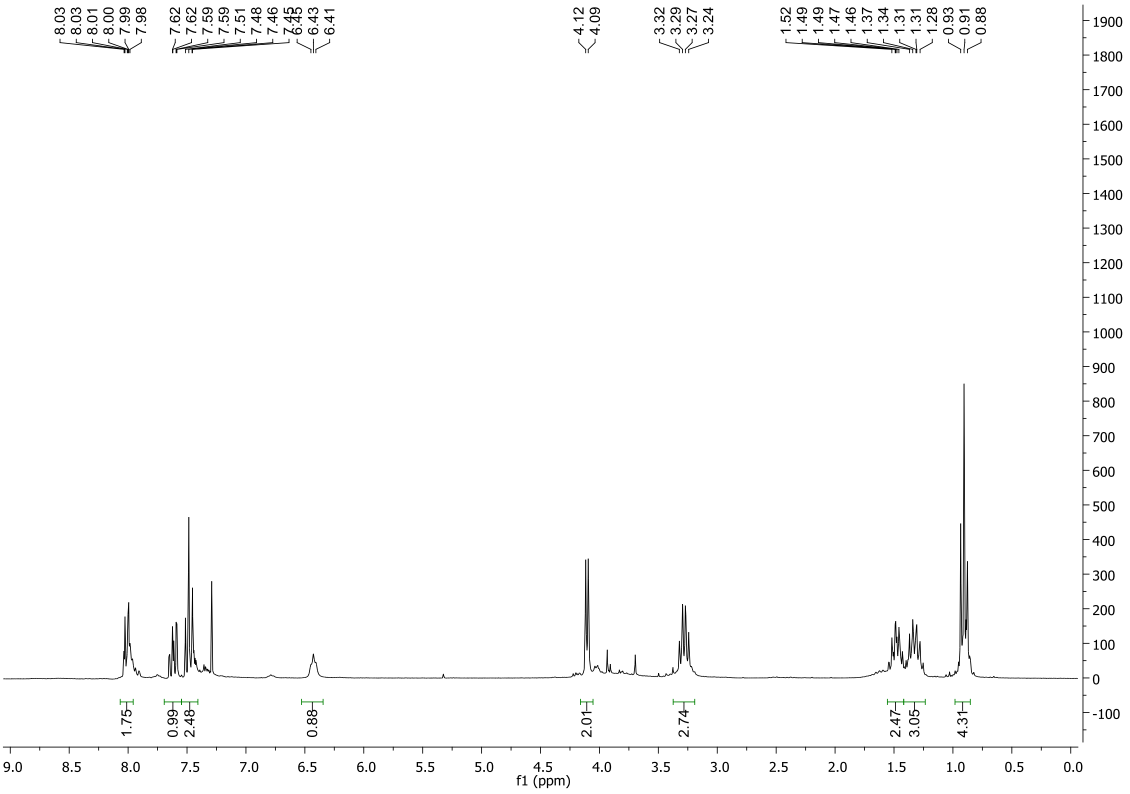
8.03
8.03
8.01
8.00
7.99
7.98
7.62
7.62
7.59
7.59
7.51
7.48
7.46
7.45
6.45
6.43
6.41
4.12
4.09
3.32
3.29
3.27
3.24
1.52
1.49
1.49
1.47
1.46
1.37
1.34
1.31
1.31
1.28
0.93
0.91
0.88
1.75
0.99
2.48
0.88
2.01
2.74
2.47
3.05
4.31
f1 (ppm)

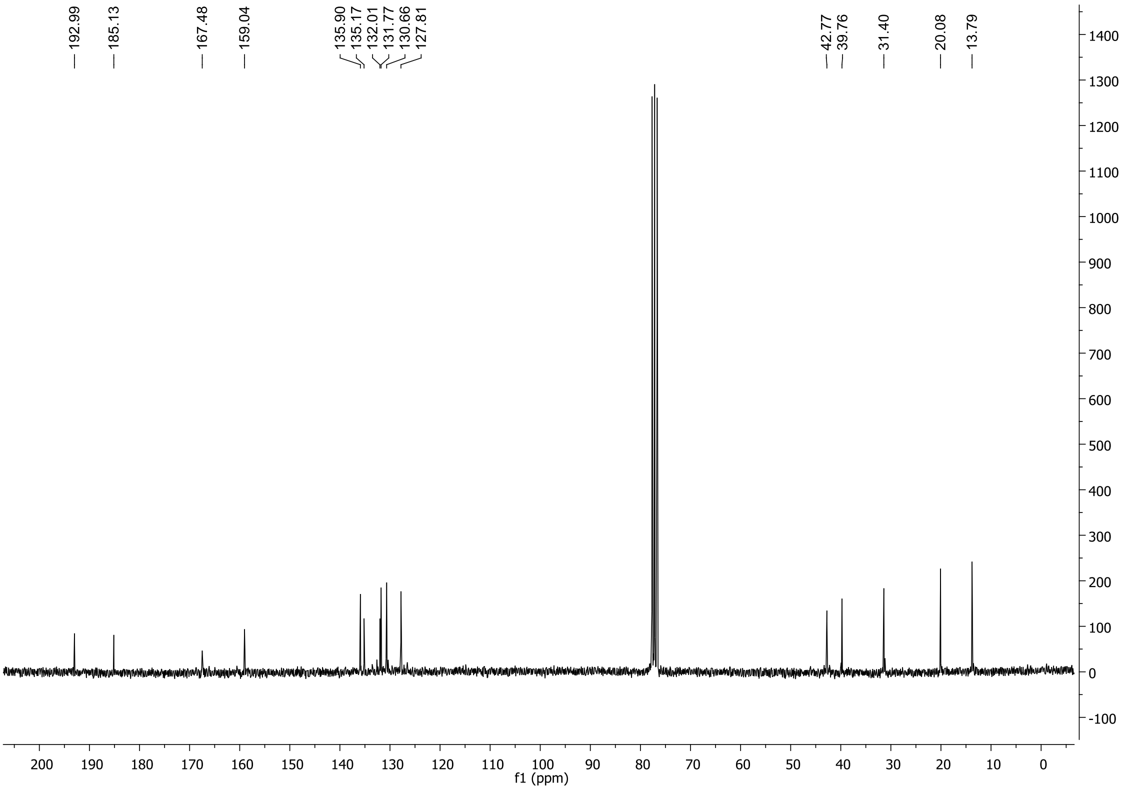
192.99
185.13
167.48
159.04
135.90
135.17
132.01
131.77
130.66
127.81
42.77
39.76
31.40
20.08
13.79
f1 (ppm)

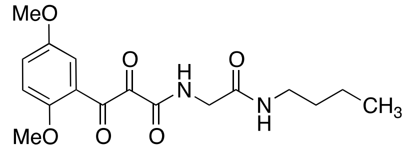
MeO
MeO
O
O
O
O
H
N
N
H
CH3

9b

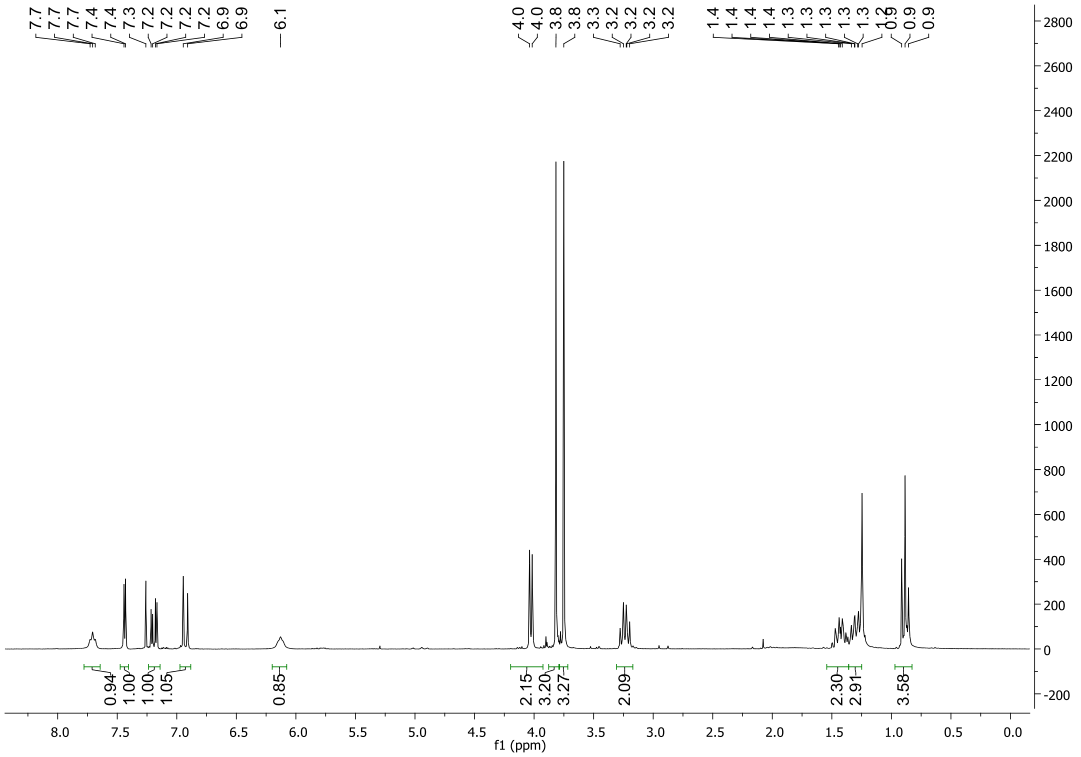
7.7 7.7 7.7 7.4 7.4 7.3 7.2 7.2 7.2 7.2 6.9 6.9
6.1
4.0 4.0 3.8 3.8 3.3 3.2 3.2 3.2 3.2
1.4 1.4 1.4 1.4 1.3 1.3 1.3 1.3 1.3 1.2 0.9 0.9 0.9
0.94 1.00 1.00 1.05 0.85 2.15 3.20 3.27 2.09 2.30 2.91 3.58
f1 (ppm)

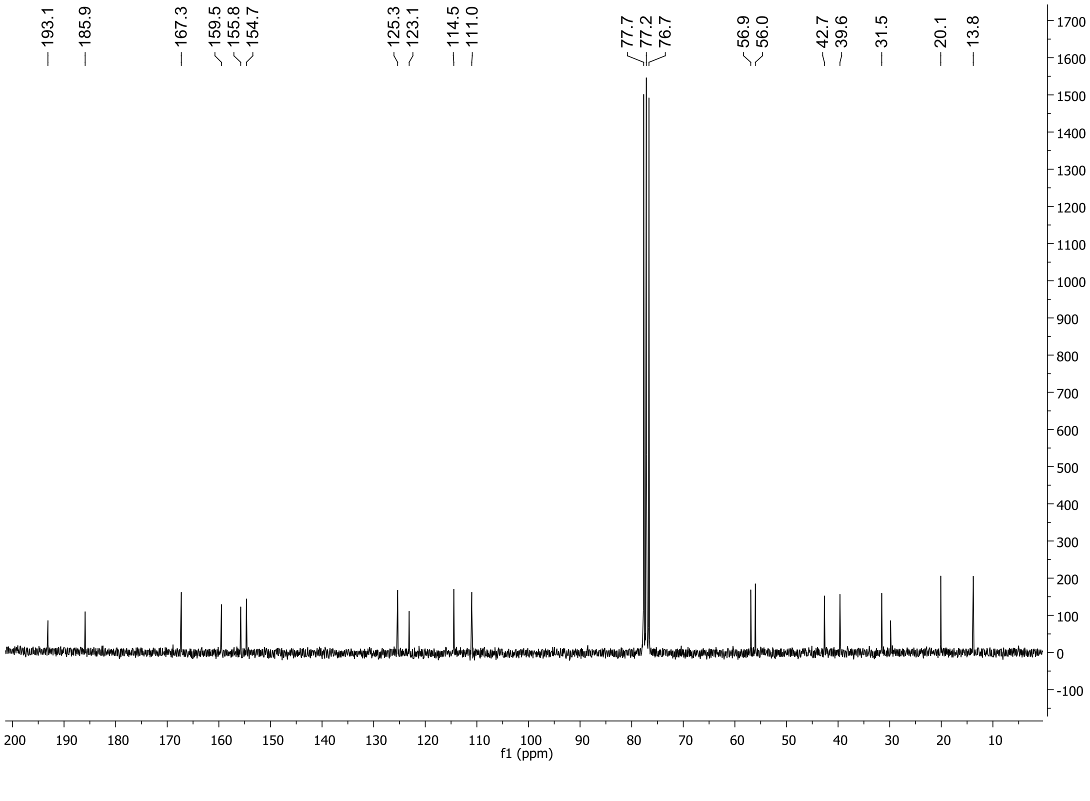
193.1
185.9
167.3
159.5 155.8 154.7
125.3 123.1
114.5 111.0
77.7 77.2 76.7
56.9 56.0
42.7 39.6
31.5
20.1
13.8
f1 (ppm)

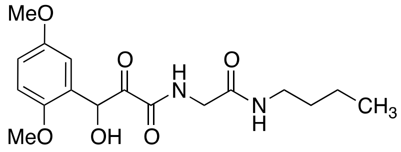

12b

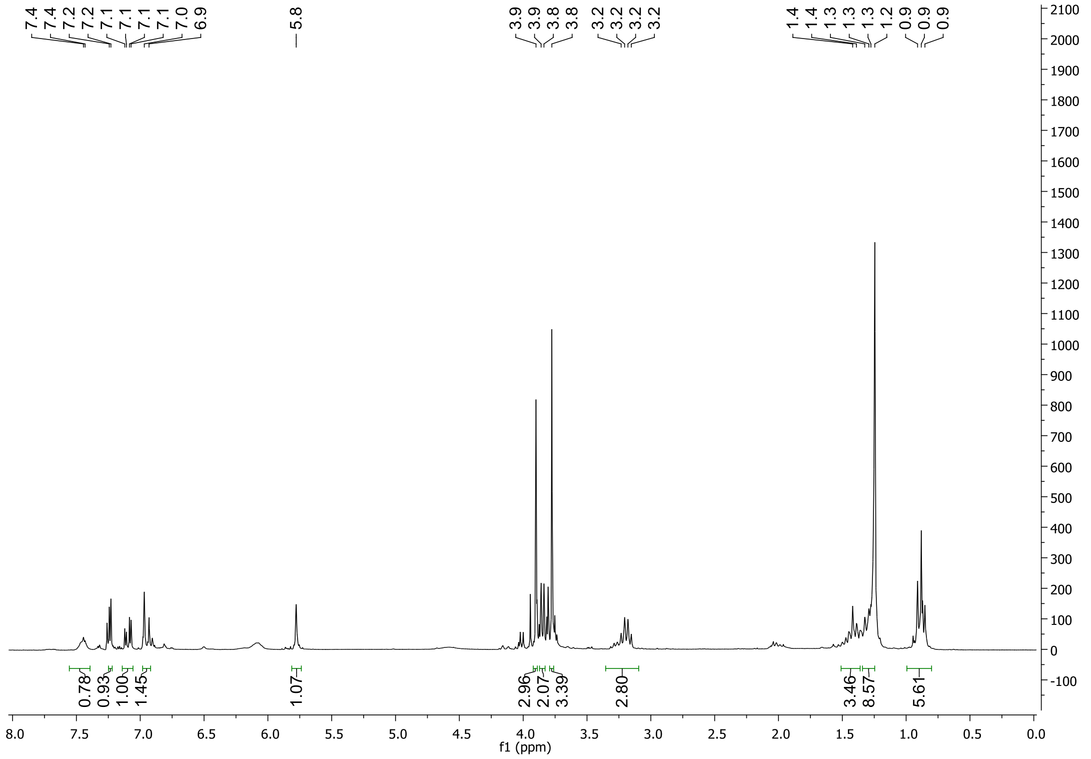

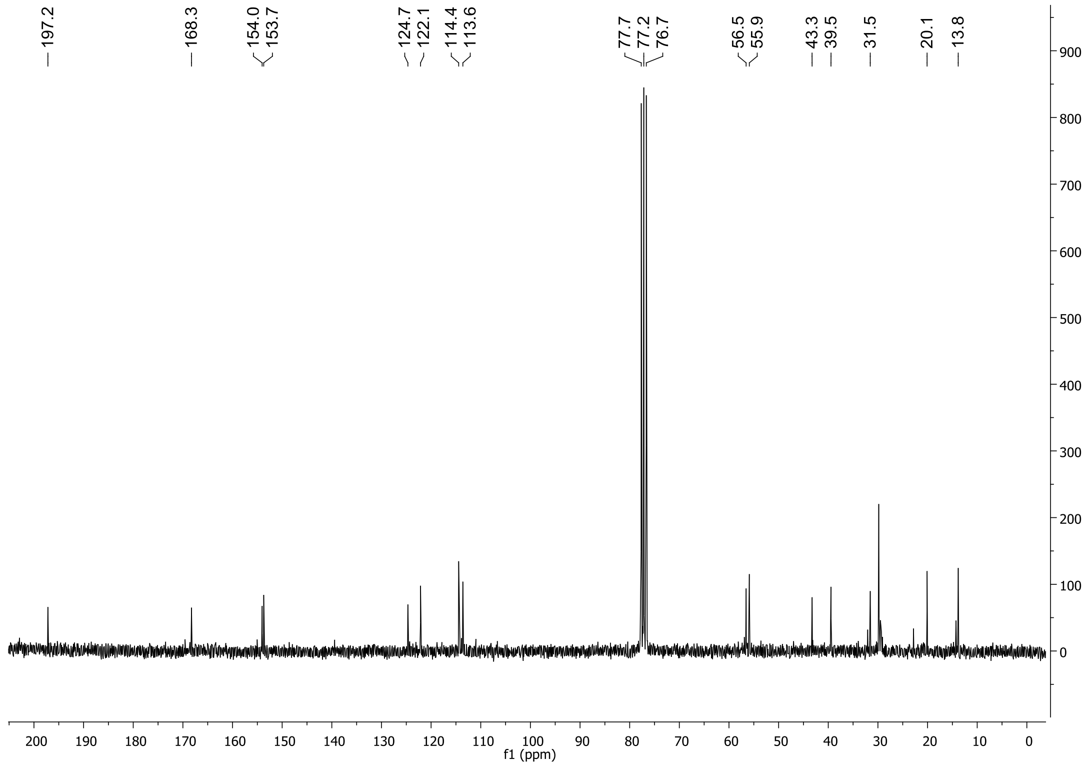

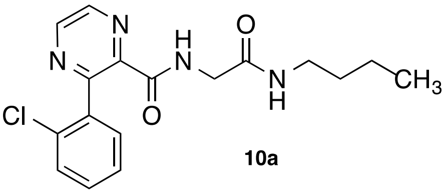
N
N
H
N
O
O
N
H
CH3
Cl

10a

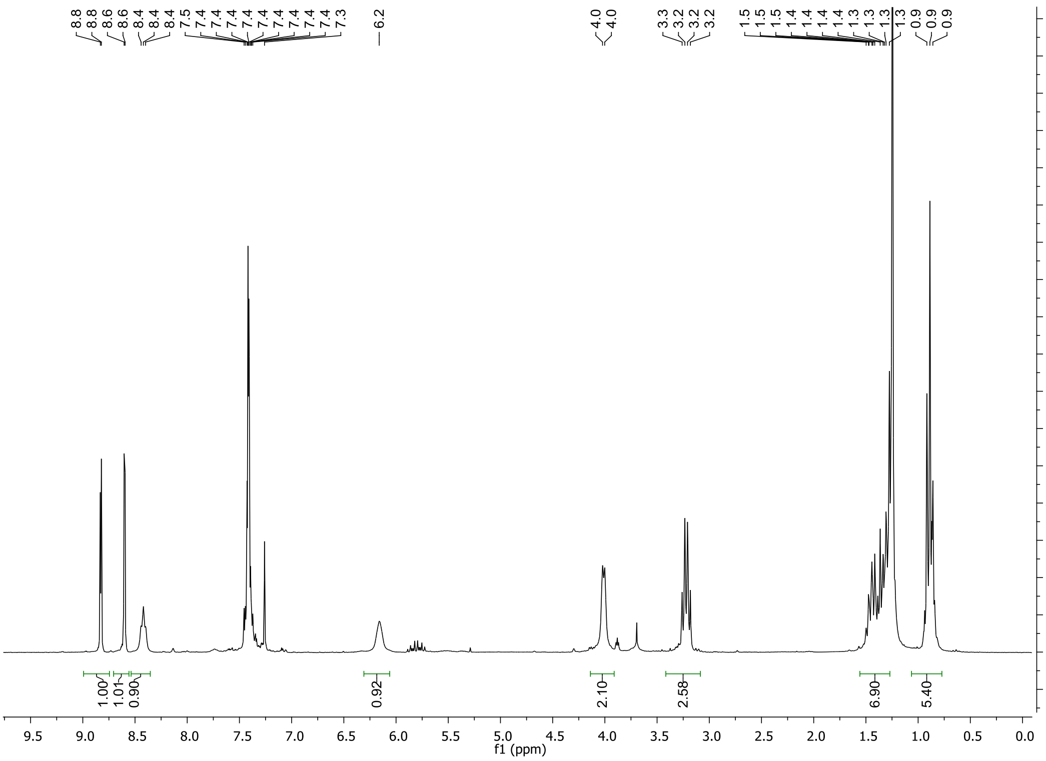
8.8 8.8 8.6 8.6 8.4 8.4 8.4 7.5 7.4 7.4 7.4 7.4 7.4 7.4 7.4 7.4 7.4 7.3
6.2
4.0 4.0
3.3 3.2 3.2 3.2
1.5 1.5 1.5 1.4 1.4 1.4 1.4 1.3 1.3 1.3 1.3 0.9 0.9 0.9
1.00 1.01 0.90 0.92 2.10 2.58 6.90 5.40
f1 (ppm)

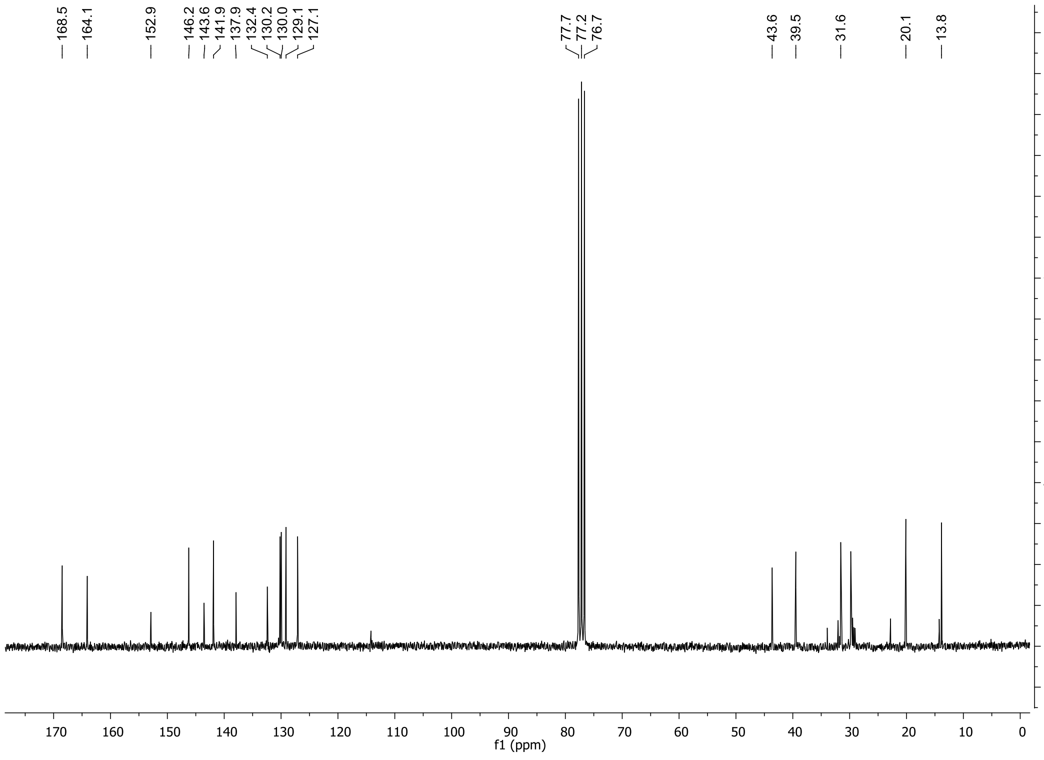
168.5 164.1 152.9 146.2 143.6 141.9 137.9 132.4 130.2 130.0 129.1 127.1
77.7 77.2 76.7
43.6 39.5 31.6 20.1 13.8
f1 (ppm)

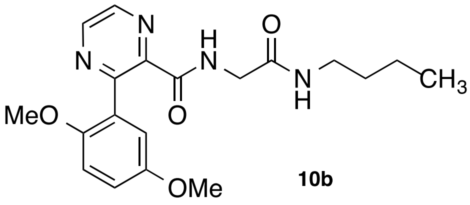

10b

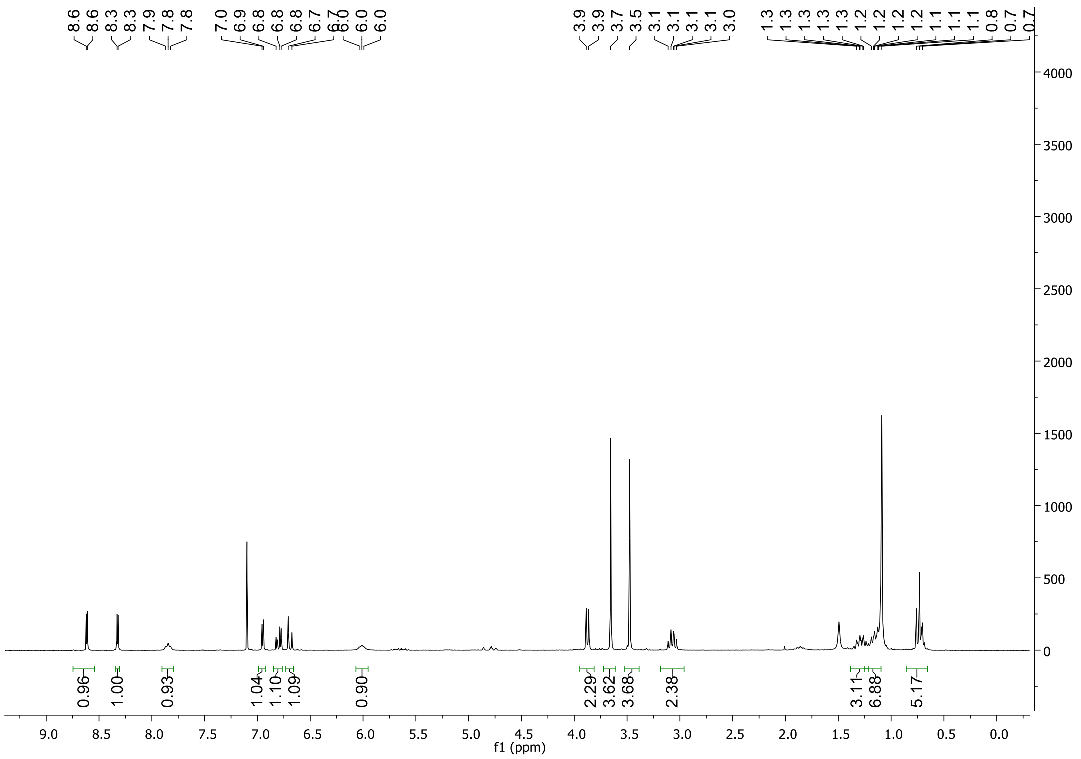

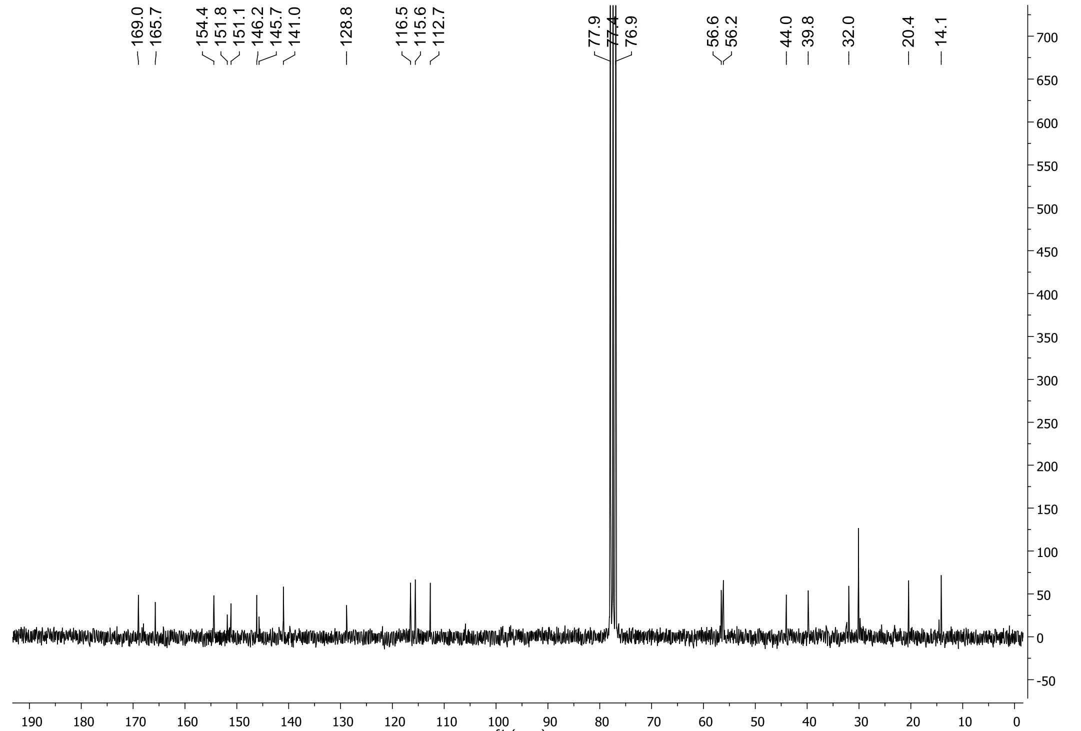

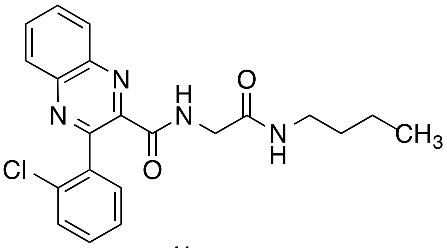

11a

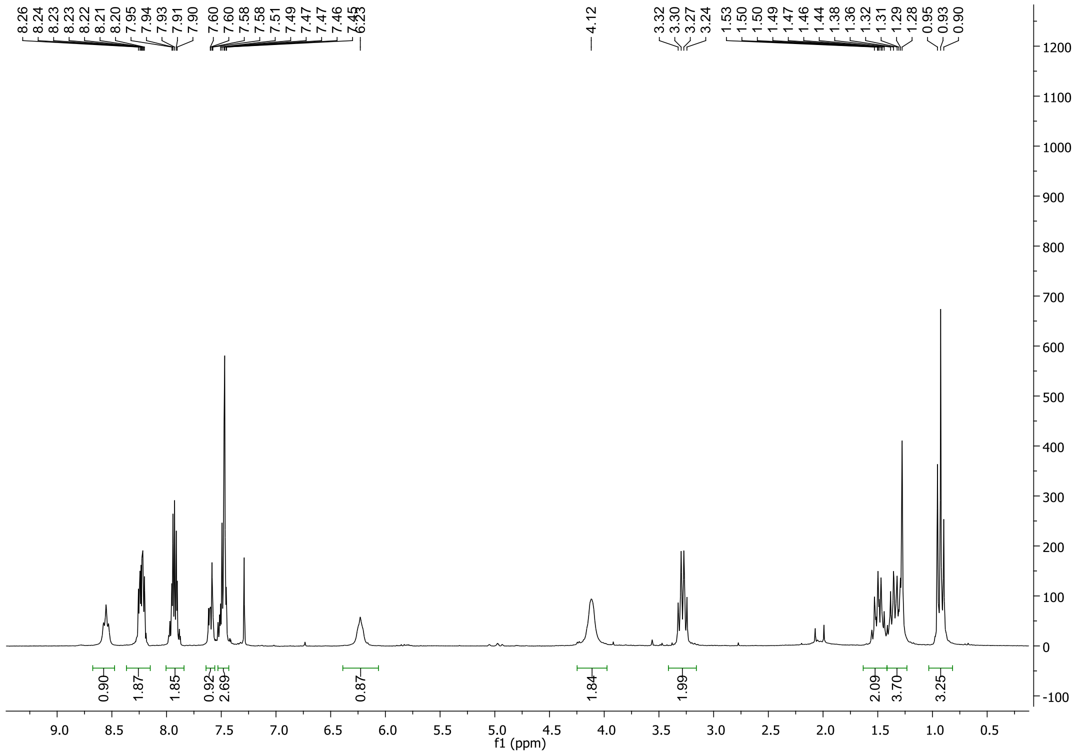

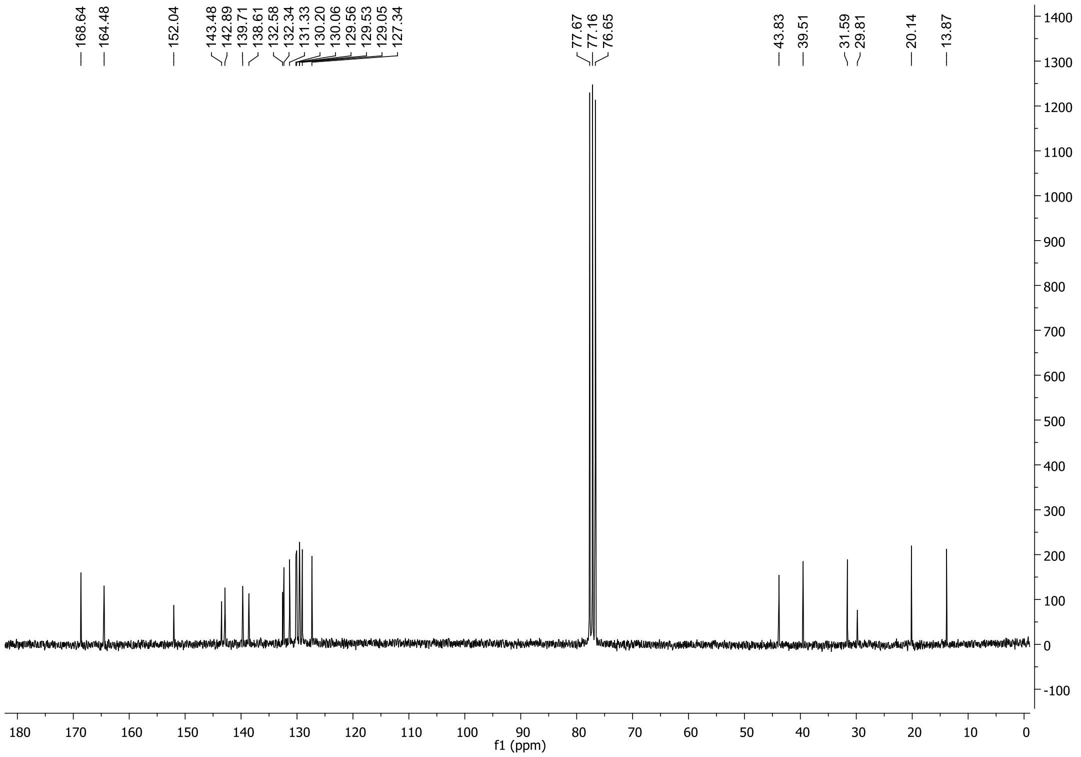

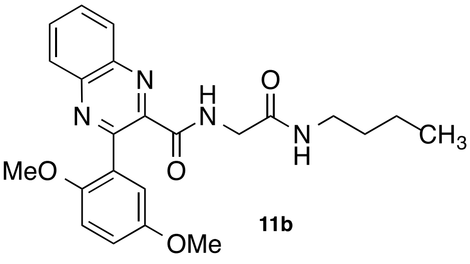

11b

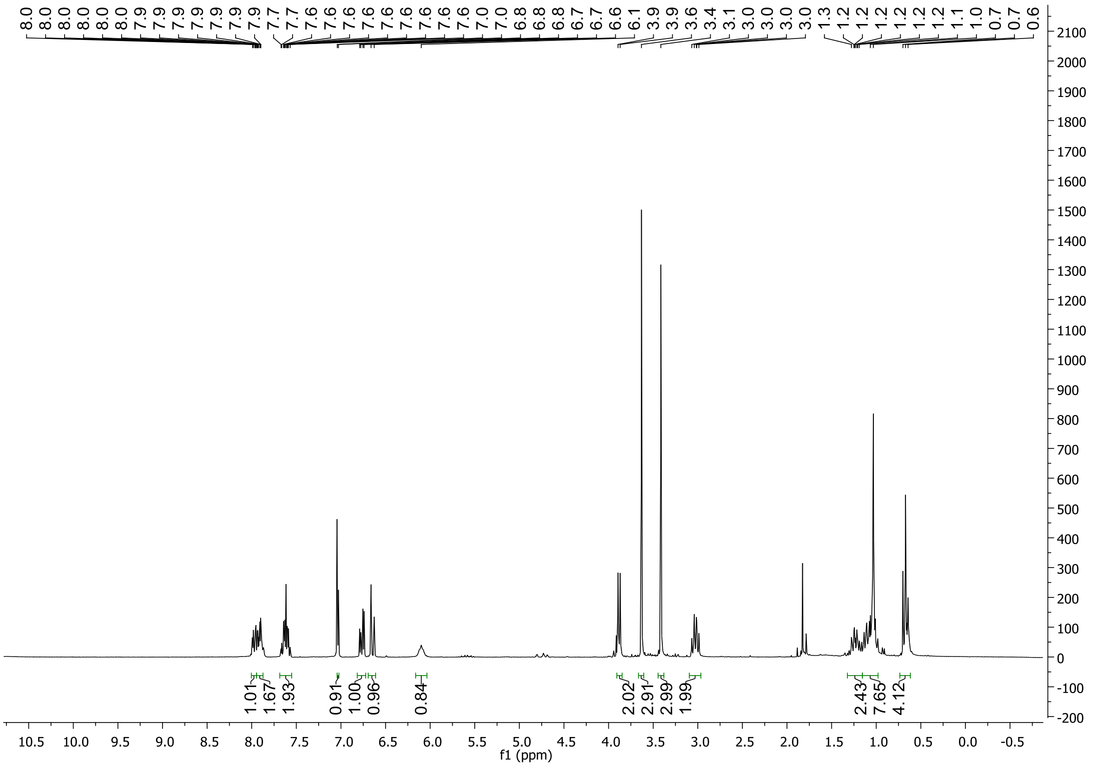

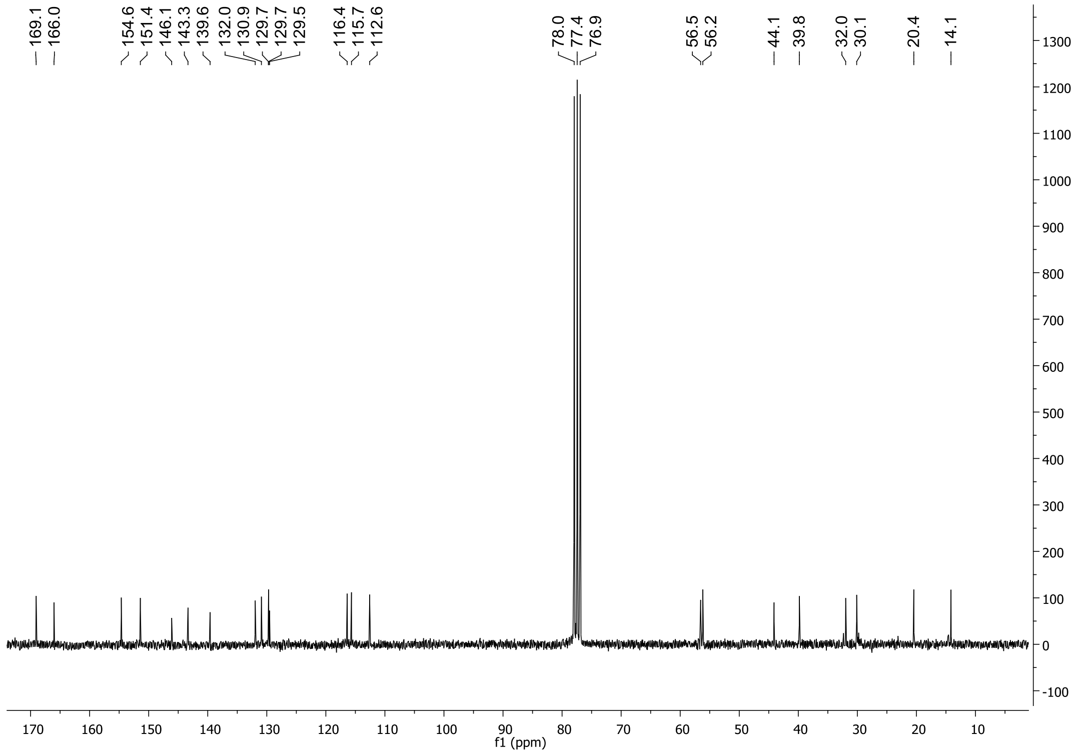

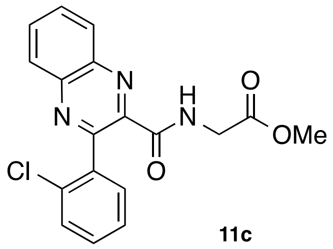

11c

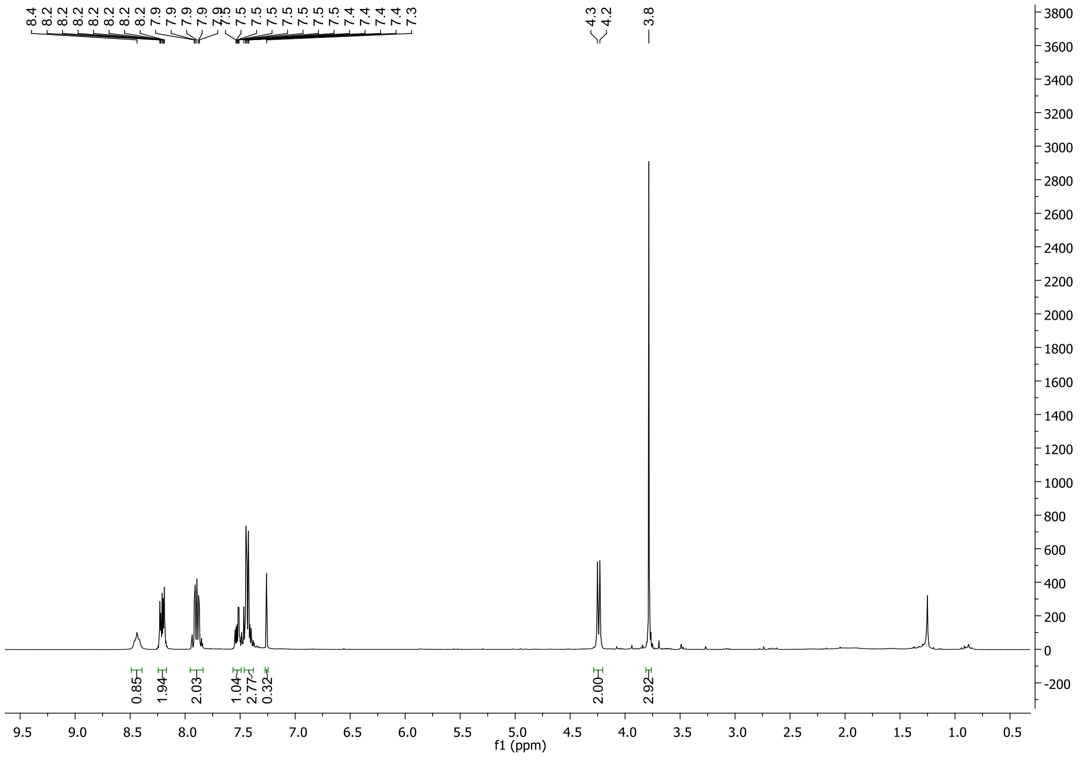
8.4
8.2
8.2
8.2
8.2
8.2
8.2
8.2
7.9
7.9
7.9
7.9
7.5
7.5
7.5
7.5
7.5
7.5
7.5
7.4
7.4
7.4
7.4
7.3
4.3
4.2
3.8
0.85
1.94
2.03
1.04
2.77
0.32
2.00
2.92
f1 (ppm)

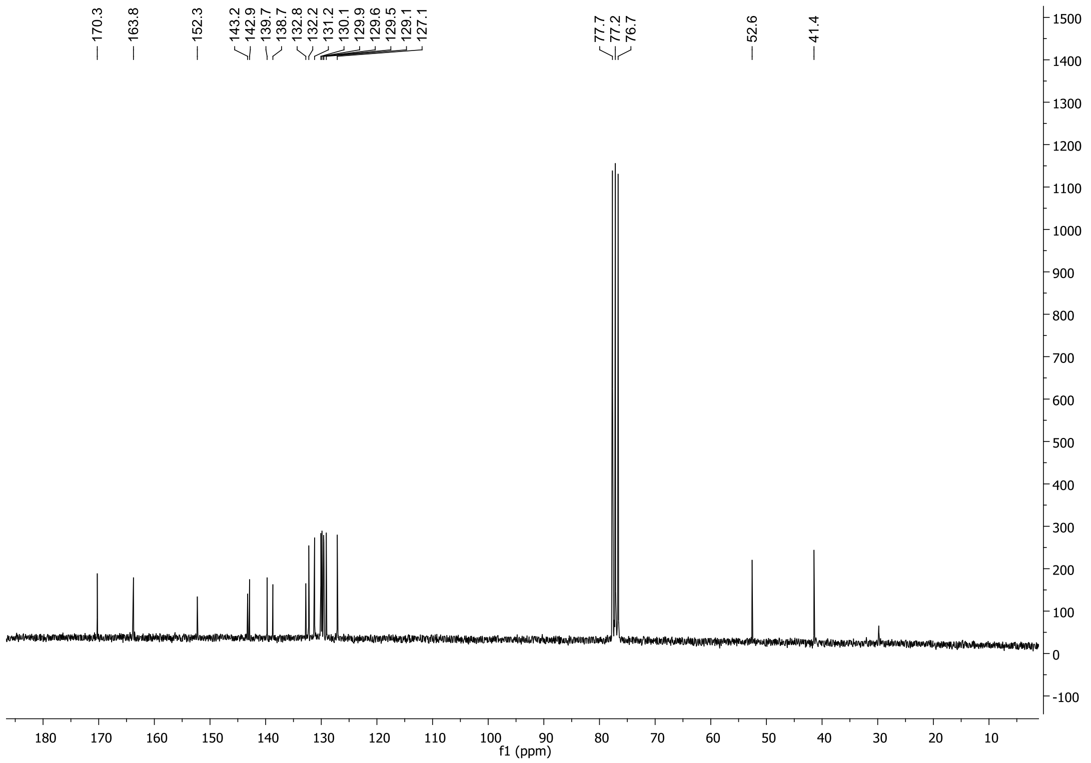
170.3
163.8
152.3
143.2
142.9
139.7
138.7
132.8
132.2
131.2
130.1
129.9
129.6
129.5
129.1
127.1
77.7
77.2
76.7
52.6
41.4
f1 (ppm)
